# Supplementary material for: Genome-Wide Analysis of Long Non-Coding RNAs Related to UV-B Radiation in the Antarctic Moss Pohlia nutans
Source: Int J Mol Sci. 2023 Mar 17;24(6):5757. doi: 10.3390/ijms24065757 (PMC10051584; doi:10.3390/ijms24065757)
Supplement: Supplementary file 1 [file ijms-24-05757-s001.zip › supplementary material.pdf]

International Journal of
*Molecular Sciences*

*Article*

# Genome-Wide Analysis of Long Non-Coding RNAs Related to UV-B Radiation in the Antarctic Moss *Pohlia nutans*

**Shuo Fang [1], Bailin Cong [1], Linlin Zhao [1,2], Chenlin Liu [1], Zhaohui Zhang [1,2] and Shenghao Liu [1,2,*]**

[1] Key Laboratory of Marine Eco-Environmental Science and Technology, First Institute of Oceanography, Ministry of Natural Resources, Qingdao 266061, China; fangshuo@fio.org.cn (S.F.); biolin@fio.org.cn (B.C.); zhaolinlin@fio.org.cn (L.Z.); chenlinliu@fio.org.cn (C.L.); zhang@fio.org.cn (Z.Z.)
[2] Laboratory for Marine Ecology and Environmental Science, Pilot National Laboratory for Marine Science and Technology (Qingdao), Qingdao 266061, China
* Correspondence: shliu@fio.org.cn

**Citation:** Fang, S.; Cong, B.; Zhao, L.; Liu, C.; Zhang, Z.; Liu, S. Genome-Wide Analysis of Long Non-Coding RNAs Related to UV-B Radiation in the Antarctic Moss *Pohlia nutans*. **2023**, *22*, x. https://doi.org/10.3390/xxxxx

Academic Editor(s):

Received: date
Accepted: date
Published: date

**Publisher's Note:** MDPI stays neutral with regard to jurisdictional claims in published maps and institutional affiliations.

**Copyright:** © 2023 by the authors. Submitted for possible open access publication under the terms and conditions of the Creative Commons Attribution (CC BY) license (https://creativecommons.org/licenses/by/4.0/).

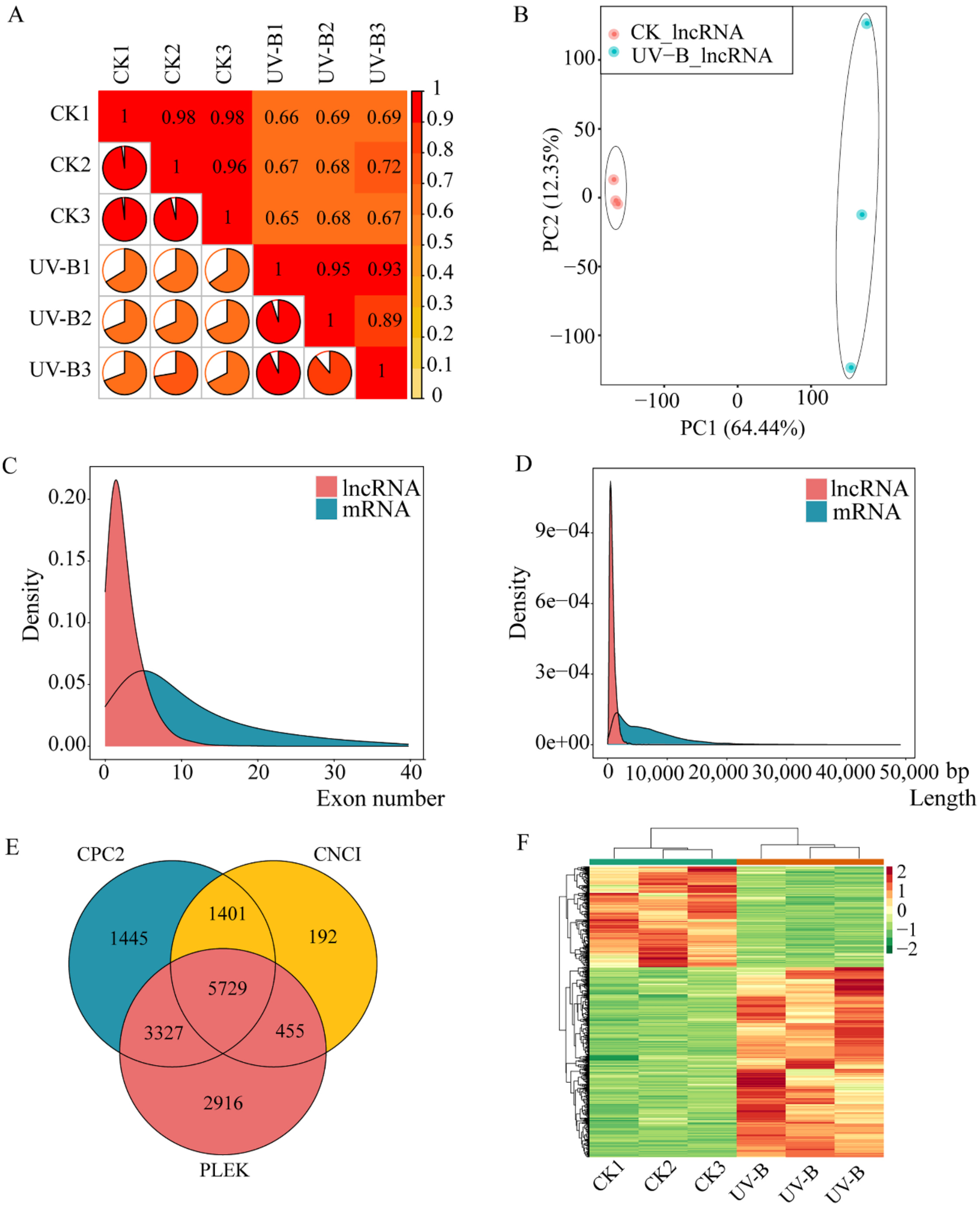


**Supplementary figure 1**: Overall assessment of data quality and changes of RNA-sequencing. (A) The Pearson correlation coefficient was used to calculate the predictive accuracy of model com-parison of RNA-sequencing data between different groups. The closer Pearson correlation coef-ficient approaches 1, the greater the degree of correlation. (B) Principal com-ponent analysis (PCA) of transcriptome data from different groups. CK was the control without UV-B radiation treatment. UV-B is the abbreviation of ultraviolet B radiation. Here, each group replicates three biological agents. (C) Exon number distribution of LncRNA and mRNA. (D) Transcript length distribution of LncRNA and mRNA. (E) Prediction results of coding potential of LncRNA by using three methods (CPC2, CNCI and PLEK). (F) Clustering heat map of differentially expressed lncRNA (DELs).

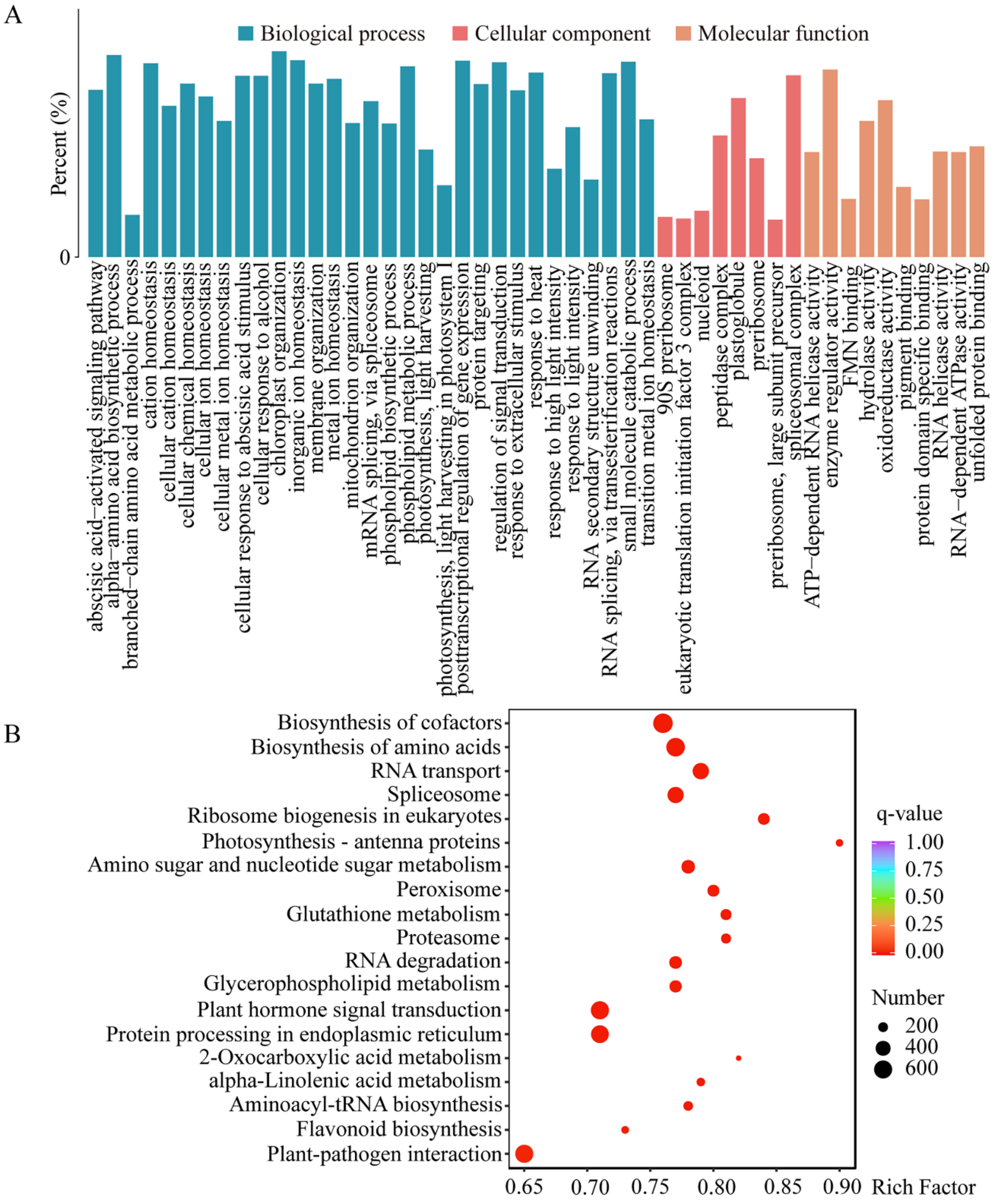


**Supplementary figure 2**: Functional annotation of the target gene of DELs. (A) GO enrichment analysis of the target gene of DELs. (B) KEGG pathway enrichment of the target gene of DELs. Rich factor represents the ratio of the number of the target gene of DELs to the total number of annotated genes in this pathway.

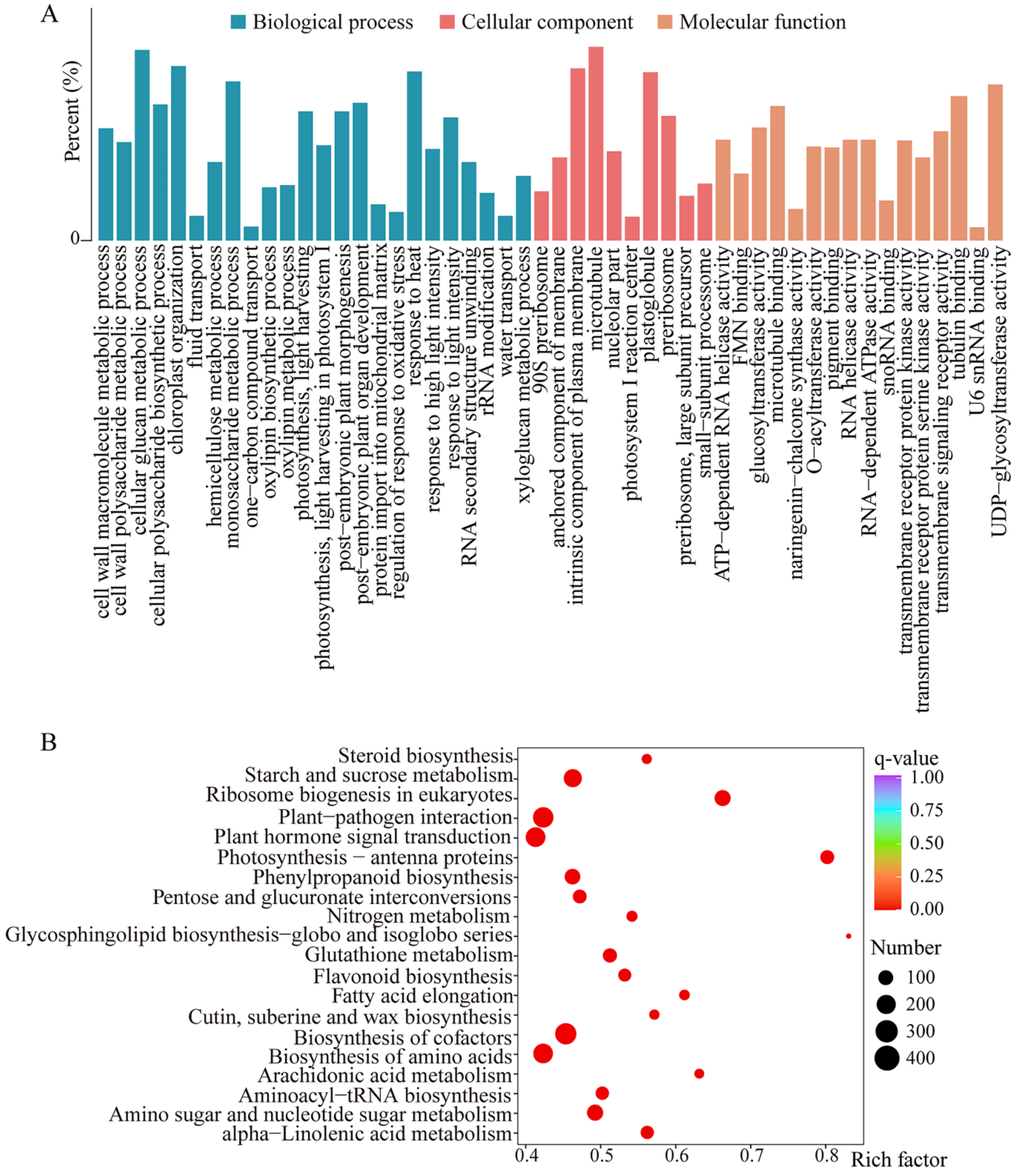


**Supplementary figure 3**: Statistical analysis of differentially expression genes (DEGs). (A) GO enrichment analysis of DEGs. (B) Statistics of KEGG enrichment for the DEGs.
