# Supplementary material for: Genome-Wide Analysis of Long Non-Coding RNAs Related to UV-B Radiation in the Antarctic Moss Pohlia nutans
Source: Int J Mol Sci. 2023 Mar 17;24(6):5757. doi: 10.3390/ijms24065757 (PMC10051584; doi:10.3390/ijms24065757)
Supplement: Supplementary file 1 [file ijms-24-05757-s001.zip › Supplementary Table1.pdf]

Supplementary Table 1. Summary of reads mapped to the Pohlia nutans reference genome.

**Sample**	**Total Reads**	**Reads mapped**	**Unique mapped**	**Multi mapped**	**'+' mapped**	**'-' mapped**
CK_1	79469444	40622188 (51.12%)	35697321 (44.92%)	12239811 (6.20%)	17839738 (22.45%)	17857583 (22.47%)
CK_2	98500292	48726498 (49.47%)	42411324 (43.06%)	16007637 (6.41%)	21193549 (21.52%)	21217775 (21.54%)
CK_3	88589850	46989655 (53.04%)	41394267 (46.73%)	14054358 (6.32%)	20689391 (23.35%)	20704876 (23.37%)
UV-B_1	86334606	52768543 (61.12%)	46515322 (53.88%)	16117273 (7.24%)	23245817 (26.93%)	23269505 (26.95%)
UV-B_2	83986726	49692638 (59.17%)	43189364 (51.42%)	17526197 (7.74%)	21580859 (25.70%)	21608505 (25.73%)
UV-B_3	95201204	60686612 (63.75%)	53654058 (56.36%)	17929434 (7.39%)	26812209 (28.16%)	26841849 (28.19%)

Supplementary Table 2. All detected lncRNAs in *Pohlia nutans* under UV-B Radiation

ID	CK_1_fpk	CK_2_fpk	CK_3_fpk	UV-B_1_fp	UV-B_2_fp	UV-B_3_fp	CK_1_cour	CK_2_coun	CK_3_coun	UV-B_1_co	UV-B_2_co	UV-B_3_co	lncRNA_Ge	Gene_Type	Chromoso	Start	End	Strand	Exon_num	Length
LNC_0039	1.518724	1.423788	0.836018	1.141652	0.587681	0.755117	34	37	22	34	16	26	XLOC_039	lncRNA	chr5	14947018	14948627	+	6	665
LNC_0039	5.335126	4.009301	4.624733	11.7692	13.27985	12.68952	138	124	139	407	423	501	XLOC_039	lncRNA	chr5	15935299	15936589	+	3	786
LNC_0054	105.0765	99.24392	14.70817	53.52027	50.74772	68.49148	3047	3429	495	2082	1818	3041	XLOC_054	lncRNA	ptg000922	47913	48797	-	1	885
LNC_0054	1.591761	2.308653	0.211081	2.729302	2.276848	3.071128	26	45	4	60	46	77	XLOC_054	lncRNA	ptg000886	20092	20589	-	1	498
LNC_0054	13.24949	17.56809	1.775345	3.983604	3.652421	3.480217	90	142	14	37	31	36	XLOC_054	lncRNA	ptg000886	3911	4116	-	1	206
LNC_0054	18.92402	24.04369	2.685933	7.268081	7.569364	14.67233	1499	2269	247	773	741	1779	XLOC_054	lncRNA	ptg000886	16464	18880	+	1	2417
LNC_0054	0.395138	0.965671	0.931635	1.748604	1.372575	2.001872	11	30	28	61	44	80	XLOC_054	lncRNA	ptg000890	41271	42057	-	1	787
LNC_0054	7.453538	6.252488	5.51354	4.868882	7.651684	3.487577	68	68	59	60	87	49	XLOC_054	lncRNA	ptg000890	334	611	-	1	278
LNC_0054	6.78915	7.546963	9.547803	7.805409	12.7113	6.133448	73	97	119	113	169	101	XLOC_054	lncRNA	ptg000890	38355	38682	+	1	328
LNC_0039	0.761457	0.782924	1.451672	1.167127	0.758328	0.692952	12	14	25	23	14	16	XLOC_039	lncRNA	chr5	14949249	14951197	+	5	441
LNC_0039	0.701213	1.302407	0.794752	0.727266	1.306806	0.426415	11	23	14	15	24	10	XLOC_039	lncRNA	chr5	14752319	14754269	+	5	452
LNC_0039	0.195037	0.712888	1.793454	0	0.168503	0	2	8	18	0	2	0	XLOC_039	lncRNA	chr5	14766381	14766644	+	1	264
LNC_0039	3.003627	3.46825	3.274016	13.70171	12.61605	15.21532	77	106	98	471	400	597	XLOC_039	lncRNA	chr5	16010010	16011296	+	3	782
LNC_0039	1.336244	0.740139	0.56966	0.064369	0	0.049071	31	21	16	2	0	2	XLOC_039	lncRNA	chr5	15204630	15205336	+	1	707
LNC_0039	2.591552	2.019353	1.601259	0.524319	0.934665	0.85411	129	120	93	35	58	66	XLOC_039	lncRNA	chr5	15755495	15759972	+	7	1518
LNC_0039	1.235821	1.322536	1.253929	1.239361	1.451467	1.600105	64	82	76	86	93	127	XLOC_039	lncRNA	chr5	16172956	16177565	+	11	1576
LNC_0039	297.0049	358.1787	255.5752	71.60098	95.37496	216.304	3299	4740	3292	1067	1309	3678	XLOC_039	lncRNA	chr5	16431322	16431660	+	1	339
LNC_0014	1.445089	0.13345	0.073207	0.193883	0	0	17	2	1	3	0	0	XLOC_015	lncRNA	chr15	12498836	12499420	+	2	338
LNC_0014	1.989432	1.254965	1.639746	1.596042	1.112519	1.332226	30	22	28	32	21	30	XLOC_015	lncRNA	chr15	12797958	12798402	+	1	445
LNC_0014	89.95779	113.1838	112.2362	38.94077	35.62756	89.89481	870	1304	1258	505	426	1331	XLOC_015	lncRNA	chr15	13150403	13150697	+	1	295
LNC_0014	2.443699	3.563028	3.205245	1.611528	1.556406	1.817638	109	189	166	97	86	124	XLOC_015	lncRNA	chr15	13457114	13461472	+	4	1357
LNC_0014	1.075731	0.858932	1.019619	0.811352	1.517241	1.268365	39	38	43	40	68	71	XLOC_015	lncRNA	chr15	13461507	13464126	+	4	1106
LNC_0014	0.290232	0.431412	1.020131	1.385046	0.139687	0.085534	3	4	9	15	2	1	XLOC_015	lncRNA	chr15	13467825	13468054	+	1	230
LNC_0014	1076.831	1392.928	441.5763	310.0965	287.0692	855.7864	12346	19032	5872	4770	4066	15023	XLOC_015	lncRNA	chr15	13669749	13670098	+	1	350
LNC_0014	2.442199	1.550926	0.659829	0.664708	1.305924	0.378167	43	33	14	16	28	11	XLOC_015	lncRNA	chr15	13863746	13864274	+	1	529
LNC_0014	0.286643	0.320719	0.493514	1.406804	1.122887	2.411975	7	8	12	40	30	78	XLOC_015	lncRNA	chr15	14296668	14297925	+	2	639
LNC_0044	1.838087	0.968719	0.795772	0.798868	0.811778	0.992724	69	44	35	40	38	57	XLOC_045	lncRNA	chr6	19132981	19138841	-	9	1138
LNC_0044	0.737616	0.262954	0	0.738095	1.680893	0.198991	21	9	0	28	58	9	XLOC_045	lncRNA	chr6	18527443	18529428	-	5	841
LNC_0044	28.56713	25.50884	19.77465	15.3215	13.57358	22.01092	580	617	466	417	340	684	XLOC_045	lncRNA	chr6	18913838	18915909	-	4	619
LNC_0044	1.245501	1.285574	1.028328	0.973388	0.512536	0.529628	63	78	61	67	32	41	XLOC_045	lncRNA	chr6	20176297	20177839	-	1	1543
LNC_0044	0.920299	1.553902	1.301954	5.79987	4.233806	4.175126	70	141	115	592	398	486	XLOC_045	lncRNA	chr6	20564002	20566321	-	1	2320
LNC_0044	4.63132	4.099241	6.378221	0.957401	1.561755	2.015557	171	180	272	48	71	114	XLOC_045	lncRNA	chr6	19945050	19947275	-	2	1121
LNC_0044	6.715195	4.154417	3.920151	7.850835	7.421054	12.39519	184	136	125	289	251	520	XLOC_045	lncRNA	chr6	20741730	20743935	-	5	835
LNC_0032	0	0.02738	0	1.982942	0.909133	1.657307	0	2	0	163	69	156	XLOC_033	lncRNA	chr3	31739292	31741156	+	1	1865
LNC_0032	1.394961	1.16669	1.266536	0.390074	0.585129	0.333069	16	16	17	7	9	6	XLOC_033	lncRNA	chr3	31660791	31661255	+	2	350
LNC_0032	1.047099	1.046496	0.994799	1.87563	1.754308	0.898516	51	61	56	122	105	67	XLOC_033	lncRNA	chr3	29602851	29604432	+	2	1479
LNC_0032	0.278513	0.400644	0.255134	0.491294	1.517295	0.420812	11	19	12	26	73	25	XLOC_033	lncRNA	chr3	29638492	29641285	+	6	1176
LNC_0032	0.339136	0.571808	0.067554	0.26942	1.326668	0.737392	13	27	3	14	63	44	XLOC_033	lncRNA	chr3	29635784	29637888	+	5	1169
LNC_0032	41.53693	44.22018	30.07686	20.93798	17.56373	30.70541	723	917	607	489	378	818	XLOC_033	lncRNA	chr3	30940663	30942950	+	4	531
LNC_0032	2.459536	2.101641	1.65468	0.789395	0.484628	0.818275	135	137	105	58	33	69	XLOC_033	lncRNA	chr3	30989724	30993401	+	7	1667
LNC_0007	0	0	0.402104	1.259324	0.628982	1.040596	0	0	8	27	13	26	XLOC_007	lncRNA	chr11	18441648	18443778	-	5	484
LNC_0007	1.223871	2.371481	1.512648	1.515277	2.231045	1.53117	48	109	68	79	107	91	XLOC_007	lncRNA	chr11	18519898	18521226	-	2	1175
LNC_0007	0.913995	0.428876	0.568863	0.753194	1.355836	1.006637	27	15	20	30	49	45	XLOC_007	lncRNA	chr11	19259953	19260842	-	1	890
LNC_0007	1.81716	0.653379	1.387023	1.147658	0.38492	1.200186	37	16	33	32	10	38	XLOC_007	lncRNA	chr11	19067739	19069752	-	4	618
LNC_0007	0.181685	0.274596	0.248384	0.772219	1.351455	1.507711	10	18	16	57	92	127	XLOC_008	lncRNA	chr11	20226920	20228597	-	1	1678
LNC_0007	1.821608	1.506333	1.306307	0.917658	2.464428	0.959763	55	54	46	37	91	44	XLOC_007	lncRNA	chr11	20091752	20094119	-	6	912
LNC_0007	1.744116	0	0.178006	0	0	0	16	0	2	0	0	0	XLOC_008	lncRNA	chr11	21048178	21048456	-	1	279
LNC_0007	0.905924	0.879762	0.271066	1.414459	1.155093	1.301217	11	13	4	24	18	25	XLOC_008	lncRNA	chr11	20814399	20816209	-	2	370
LNC_0031	5.040309	4.713145	3.958124	2.088627	2.526184	1.876426	246	274	224	137	152	140	XLOC_031	lncRNA	chr23	9340858	9342692	+	2	1485
LNC_0031	1.243372	0.982433	1.109115	0.808697	0.796297	1.787107	30	29	31	26	24	66	XLOC_031	lncRNA	chr23	2757807	2758934	+	3	731
LNC_0031	0.072255	0.121266	0.093456	0.269282	1.22721	0.376119	3	4	3	11	42	16	XLOC_031	lncRNA	chr23	1257607	1258451	+	1	845
LNC_0031	0.535352	0.973075	1.338483	0.425947	0.404231	0.71118	7	14	19	7	6	13	XLOC_031	lncRNA	chr23	203544	204285	+	3	354
LNC_0031	1.575189	1.236441	1.309699	0.739166	0.36478	0.661186	28	27	27	18	8	18	XLOC_031	lncRNA	chr23	12065690	12066347	+	2	542
LNC_0031	1.803922	1.914731	0	0	0	0	35	45	0	0	0	0	XLOC_031	lncRNA	chr23	11189726	11191635	+	3	591
LNC_0031	1.12108	0.050356	0.87605	0.651464	0.853634	0.258009	24	2	22	19	23	9	XLOC_031	lncRNA	chr23	11189688	11191633	+	3	646
LNC_0031	1.197691	1.512408	0.640291	1.387698	0.259717	2.141753	61	92	38	95	17	167	XLOC_031	lncRNA	chr23	9340913	9342707	+	3	1553
LNC_0031	1.25561	0.517266	0.839348	0	0.156665	0	26	13	21	0	5	0	XLOC_031	lncRNA	chr23	14638197	14638827	+	1	631
LNC_0031	8.834165	10.53592	9.091376	4.090747	4.389144	4.002204	62	87	73	38	38	43	XLOC_031	lncRNA	chr23	13577545	13577755	+	1	211
LNC_0032	4.122516	5.011596	3.877427	7.635861	3.909464	10.05841	106	153	115	262	124	394	XLOC_032	lncRNA	chr3	19027394	19030792	+	3	780
LNC_0024	0.295427	0.495545	0.112087	0.752116	1.514453	1.020726	7	12	3	21	38	32	XLOC_024	lncRNA	chr19	11028734	11029353	-	1	620
LNC_0024	2.185739	1.100101	0.806667	8.811376	6.416945	12.11986	31	18	13	163	109	255	XLOC_024	lncRNA	chr19	11068161	11068579	-	1	419
LNC_0024	1.150504	1.169006	0.949271	9.296012	7.113908	10.07784	40	49	39	433	305	535	XLOC_024	lncRNA	chr19	10642173	10644512	-	2	1058
LNC_0024	1.401795	1.073671	1.209397	0	0.239823	0	25	23	25	0	6	0	XLOC_024	lncRNA	chr19	10788172	10789322	-	2	544
LNC_0024	0.410786	0.551734	0.589846	1.649001	1.063252	0.998298	14	22	23	74	44	52	XLOC_024	lncRNA	chr19	10108126	10109350	-	2	1019
LNC_0024	3.952417	2.512479	2.151885	2.782473	1.592603	1.188593	71	54	45	67	35	33	XLOC_024	lncRNA	chr19	10636728	10639577	-	3	542
LNC_0024	1.359732	0.749021	0.546205	3.270243	2.586323	2.567919	26	17	12	84	61	75	XLOC_024	lncRNA	chr19	9657371	9659277	-	3	578
LNC_0024	0.539343	0	0	1.228182	2.077266	1.467195	12	0	0	34	53	47	XLOC_024	lncRNA	chr19	9657374	9659277	-	3	628
LNC_0024	2.450789	1.038004	1.281988	0.715136	0.93292	1.604581	25	13	16	10	12	25	XLOC_024	lncRNA	chr19	9257651	9257958	-	1	308
LNC_0024	1.022527	1.60836	1.863604	0.910437	0.485319	1.026153	58	109	123	70	34	89	XLOC_024	lncRNA	chr19	9350936	9352664	-	1	1729
LNC_0054	0.455694	0.689936	0.289624	0.935836	0.819087	0.173301	20	36	15	55	45	12	XLOC_054	lncRNA	ptg000886	17494	18823	-	1	1330
LNC_0054	5.236356	6.766366	5.292463	5.05697	6.780633	6.584747	120	184	140	155	191	230	XLOC_054	lncRNA	ptg000890	41324	42017	+	1	694
LNC_0011	0.233289	0.332616	0.110629	1.636358	0.835486	1.783588	7	12	4	66	31	82	XLOC_012	lncRNA	chr13	21601930	21602972	-	2	916
LNC_0011	3.646126	3.357231	2.386934	2.771577	3.0545	3.526112	171	188	130	174	177	253	XLOC_012	lncRNA	chr13	21232834	21235266	-	8	1428
LNC_0011	1.492304	0.449067	0.819084	1.072027	1.212522	0.522201	22	8	14	22	22	12	XLOC_012	lncRNA	chr13	21135676	21136232	-	2	448
LNC_0011	1.027626	1.524155	0.530036	0	0.095651	0.173631	24	41	14	0	3	7	XLOC_012	lncRNA	chr13	20622413	20623101	-	1	689
LNC_0011	1.443503	0.638171	0.81995	0.479507	1.8448	0.194854	25	14	17	12	40	6	XLOC_012	lncRNA	chr13	20488018	20488940	-	2	528
LNC_0011	0.272682	1.223215	0.316674	1.511024	1.019523	1.398598	6	32	8	45	28	47	XLOC_012	lncRNA	chr13	19643623	19645808	-	3	665
LNC_0011	0.427318	0.968988	0.346932	1.584431	1.241256	0.981709	17	46	17	85	61	60	XLOC_012	lncRNA	chr13	19638388	19640102	-	4	1214
LNC_0011	0.487392	0.405314	0.121306	0.57113	1.479413	0.411941	7	7	2	11	26	9	XLOC_012	lncRNA	chr13	19635198	19636422	-	3	434
LNC_0011	1.837669	0.686655	0.589472	1.211373	0.48913	1.844935	44	20	17	39	15	68	XLOC_012	lncRNA	chr13	19547939	19549072	-	3	727
LNC_0011	1.437695	1.449552	2.311748	0.098509	0.195307	0.157453	54	65	100	5	9	9	XLOC_012	lncRNA	chr13	18833941	18835078	-	1	1138
LNC_0043	3.061888	2.553119	3.230615	36.23507	22.06198	38.48264	137	136	168	2169	1216	2629	XLOC_044	lncRNA	chr6	26418554	26420008	+	2	1362
LNC_0014	0	0	0	1.306245	1.02856	0.378295	0	0	0	22	16	8	XLOC_015	lncRNA	chr15	14466057	14466439	+	1	383
LNC_0044	0.738168	0.676179	0.328778	1.637611	1.545906	2.160122	22	24	12	66	57	99	XLOC_045	lncRNA	chr6	21565457	21566837	-	4	909
LNC_0037	1.443273	1.807686	0.933764	0.672079	0.735915	1.002768	24	36	18	15	15	26	XLOC_037	lncRNA	chr4	10933540	10941420	-	2	503
LNC_0037	0	1.565923	1.394664	0	0	0.832623	0	31	27	0	0	21	XLOC_037	lncRNA	chr4	10933540	10941419	-	2	502
LNC_0037	2.164792	1.080913	1.27148	0.824785	0.797559	0.617307	80	48	55	41	37	35	XLOC_037	lncRNA	chr4	11714540	11717809	-	9	1121
LNC_0037	0	0.562444	1.462416	0.075679	0	0.237786	0	10	25	2	0	6	XLOC_037	lncRNA	chr4	11500476	11501621	-	3	450
LNC_0037	0.412261	0.182377	0.1932	1.04823	1.356464	1.107292	8	4	4	26	30	31	XLOC_037	lncRNA	chr4	11491902	11492446	-	1	545
LNC_0037	5.070927	6.614997	5.370199	1.473915	2.373514	4.081972	99	154	121	39	57	122	XLOC_037	lncRNA	chr4	10945356	10945948	-	1	593
LNC_0037	2.526359	2.260072	1.409304	0.554207	0.602737	1.219905	119	127	77	35	35	88	XLOC_037	lncRNA	chr4	12322810	12326643	-	10	1434
LNC_0037	0.762166	0.517835	0.129679	0.74477	1.449415	1.191777	13	11	3	17	30	30	XLOC_037	lncRNA	chr4	11880696	11882576	-	3	498
LNC_0053	15.42282	17.06688	12.02762	14.42633	13.12105	11.28382	352	464	319	442	370	394	XLOC_053	lncRNA	ptg000799	23778	24473	+	1	696
LNC_0053	2.754147	3.018294	2.34836	1.346964	1.182251	0.923774	78	101	77	51	41	40	XLOC_053	lncRNA	ptg000790	6541	7394	+	1	854
LNC_0053	4.297513	3.177352	0.213678	1.402302	1.502535	1.820527	85	75	5	38	37	55	XLOC_053	lncRNA	ptg000790	339	940	+	1	602
LNC_0053	0.480748	0.537002	0	1.03798	0.892552	1.568571	7	8	0	18	14	30	XLOC_053	lncRNA	ptg000790	5330	5710	+	1	381
LNC_0053	25.99735	24.03062	17.57759	11.82521	16.89464	15.01892	615	677	482	375	493	544	XLOC_053	lncRNA	ptg000778	23283	24003	-	1	721
LNC_0053	3.066044	4.448717	1.294569	2.125834	1.953096	1.578721	80	139	40	75	63	64	XLOC_053	lncRNA	ptg000804	881	1676	-	1	796
LNC_0053	15.54324	15.40279	6.105426	7.603823	13.36869	7.900477	232	273	106	152	246	180	XLOC_053	lncRNA	ptg000804	30142	30595	-	1	454
LNC_0056	2.44583	1.988054	0	1.227781	0.783657	0.915992	51	49	0	34	20	29	XLOC_055	lncRNA	ptg001536	27673	28298	+	1	626
LNC_0056	1.644299	2.817914	1.607288	2.415967	4.911081	2.542557	33	66	37	64	119	77	XLOC_055	lncRNA	ptg001537	23682	24279	+	1	598
LNC_0056	0.636365	2.018682	2.674372	4.57865	6.340201	1.813285	20	73	94	187	238	85	XLOC_055	lncRNA	ptg001521	5901	6825	-	1	925
LNC_0056	1.971377	2.399255	1.114118	1.970883	2.180299	1.578394	38	55	25	51	51	46	XLOC_055	lncRNA	ptg001531	44747	45324	-	1	578
LNC_0056	0.619518	0.171362	0.488992	0.592384	1.294055	0.619786	12	4	11	16	31	19	XLOC_055	lncRNA	ptg001534	1221	1810	+	1	590
LNC_0056	1.217296	1.795027	0.744055	0.870713	1.79931	1.804203	26	45	19	25	47	59	XLOC_055	lncRNA	ptg001534	25180	25821	+	1	642
LNC_0056	34.47648	43.39442	33.93943	17.89514	21.62926	22.25881	583	875	666	406	452	577	XLOC_055	lncRNA	ptg001534	25265	25780	-	1	516
LNC_0023	0.2333	0.893485	1.444582	0	0.228553	0	3	12	19	0	4	0	XLOC_024	lncRNA	chr19	1594361	1597403	-	2	328
LNC_0023	0.107309	0.314345	0.491128	53.0107	47.78191	48.6686	6	21	32	3973	3297	4162	XLOC_024	lncRNA	chr19	1797480	1800078	-	2	1705
LNC_0023	0.390426	0.061482	0.252144	1.601177	0.790315	2.002365	8	2	6	44	20	63	XLOC_024	lncRNA	chr19	1819060	1819684	-	1	625
LNC_0023	0.218454	0.258054	0.096278	1.900787	1.345279	2.314094	8	11	4	92	60	127	XLOC_024	lncRNA	chr19	1928108	1929318	-	2	1090
LNC_0023	0	0	0	3.619618	4.648298	4.268759	0	0	0	110	130	148	XLOC_024	lncRNA	chr19	2068346	2069036	-	1	691
LNC_0023	0.088357	0.593167	1.980928	1.020597	0.642822	0.172743	2	16	53	31	18	6	XLOC_024	lncRNA	chr19	2297457	2298147	-	1	691
LNC_0023	0.222422	0	0.095896	13.50781	11.4157	21.0177	4	0	2	326	254	579	XLOC_024	lncRNA	chr19	2384083	2384631	-	1	549
LNC_0023	0	0	0	1.674853	1.801525	1.667161	0	0	0	110	109	125	XLOC_024	lncRNA	chr19	2388502	2389993	-	1	1492
LNC_0045	1.411247	0.368575	0.25619	0.423345	0.116253	0.919147	20	6	4	8	2	19	XLOC_046	lncRNA	chr6	29321354	29321764	-	1	411
LNC_0045	0.865257	1.460566	2.565677	0.305498	0.644889	0.796984	12	23	39	6	11	16	XLOC_046	lncRNA	chr6	29241171	29241566	-	1	396
LNC_0045	1.455526	1.047428	1.388347	0.41218	0.243483	0.208686	28	24	31	11	6	7	XLOC_046	lncRNA	chr6	28136874	28137459	-	1	586
LNC_0045	1.071433	0.910322	0.815644	1.451073	0.995861	0.827222	20	20	17	35	23	23	XLOC_045	lncRNA	chr6	27366957	27367504	-	1	548
LNC_0045	0.583317	0.540914	0.377398	1.343693	1.208232	1.569518	16	18	13	50	41	66	XLOC_046	lncRNA	chr6	28918953	28921178	-	6	837
LNC_0045	1.360955	1.868927	1.003192	0.71162	0.65662	0.410466	46	75	39	32	28	22	XLOC_046	lncRNA	chr6	28165919	28169092	-	6	1023
LNC_0045	1.308695	1.316526	0.49265	1.095326	0.355978	0.371731	21	25	9	23	7	9	XLOC_046	lncRNA	chr6	28940170	28940763	-	2	467
LNC_0045	0.60146	2.046002	0.130638	0.508914	0.815209	1.926241	8	33	3	10	14	39	XLOC_046	lncRNA	chr6	28933676	28934078	-	1	403
LNC_0045	4.351704	4.169326	2.940092	0.381374	0.278198	0.467802	79	90	62	10	7	13	XLOC_046	lncRNA	chr6	29206337	29207038	-	2	552
LNC_0045	1.948328	0	0	0.21816	0.278335	0.572197	19	0	0	3	4	9	XLOC_046	lncRNA	chr6	29196428	29197860	-	2	291
LNC_0003	1.390333	1.168947	1.042181	3.96717	4.536606	4.340557	77	77	67	293	309	366	XLOC_003	lncRNA	chr1	39997638	40000617	-	7	1680
LNC_0032	0.222422	0.74628	1.00627	1.615599	1.575563	1.034578	4	16	21	39	35	29	XLOC_033	lncRNA	chr3	29313883	29314431	+	1	549
LNC_0005	2.560181	1.917138	2.17667	0.718666	1.452636	0.52532	75	67	74	28	52	24	XLOC_005	lncRNA	chr10	15712262	15715949	-	5	884
LNC_0005	1.878076	3.610705	3.572323	0	0	1.213354	59	134	129	0	0	58	XLOC_005	lncRNA	chr10	15712278	15715913	-	5	947
LNC_0005	0.457046	0.433813	1.408189	0	0.119861	0.097025	7	7	22	0	2	2	XLOC_005	lncRNA	chr10	15261823	15262233	-	1	411
LNC_0005	0	0.367241	0.444654	0.230106	1.41854	0.796645	0	9	11	6	34	24	XLOC_005	lncRNA	chr10	15513058	15515055	-	2	592
LNC_0005	0.144566	0.565996	0.124657	2.946869	4.544067	4.137845	6	28	6	165	233	263	XLOC_005	lncRNA	chr10	14920544	14921999	-	2	1267
LNC_0005	1.447165	0.350056	0.680522	0.292567	0.104379	0.313428	34	10	19	10	3	12	XLOC_005	lncRNA	chr10	14978301	14979100	-	2	704
LNC_0005	2.307681	1.95281	1.857393	1.677943	2.529353	1.90278	54	54	50	52	72	67	XLOC_005	lncRNA	chr10	14467051	14467752	-	1	702
LNC_0005	0.414468	0.398493	1.843846	0	0	0	6	7	31	0	0	0	XLOC_005	lncRNA	chr10	14853983	14854420	-	1	438
LNC_0005	4.247818	3.097521	1.468402	2.005204	1.149097	2.781817	60	52	24	38	20	60	XLOC_005	lncRNA	chr10	15799080	15799509	-	1	430
LNC_0005	580.6752	508.6187	413.1183	54.01249	35.43449	69.45803	16587	17314	13686	2070	1251	3038	XLOC_005	lncRNA	chr10	15881963	15882834	-	1	872
LNC_0032	0.109642	0.368645	0.473348	0.397688	0.597882	1.360613	2	8	10	10	14	38	XLOC_033	lncRNA	chr3	31213945	31214499	+	1	555
LNC_0007	1.268204	0.524004	0.203089	0.029914	0.301455	0.10768	14	7	3	1	5	2	XLOC_007	lncRNA	chr11	18690566	18690902	-	1	337
LNC_0050	0	0.226461	0.080747	0.297547	1.781266	0	0	3	2	5	24	0	XLOC_050	lncRNA	chr9	10936288	10986338	+	3	326
LNC_0002	0.113004	0.06905	0	0.12246	0	1.260806	3	3	0	4	0	47	XLOC_002	lncRNA	chr1	18350917	18351658	-	1	742
LNC_0009	0	0.231001	1.520575	0.263056	0	0	0	4	20	4	0	0	XLOC_010	lncRNA	chr12	28457238	28457583	-	1	346
LNC_0002	1.982216	2.270566	2.292592	4.83706	4.163274	5.007056	45	61	60	147	116	173	XLOC_002	lncRNA	chr1	19797609	19798849	-	2	688
LNC_0009	28.67278	30.4521	18.44934	20.72407	15.47313	32.5421	494	625	368	479	329	857	XLOC_010	lncRNA	chr12	25522480	25523907	-	5	525
LNC_0034	1.349281	0.324582	0.678206	0.573258	1.379969	0.564008	16	5	10	10	21	11	XLOC_034	lncRNA	chr3	19746435	19746796	-	1	362
LNC_0034	0.831713	0.768119	0.709301	2.207211	1.845755	2.403213	41	45	40	144	111	179	XLOC_034	lncRNA	chr3	20506678	20510408	-	11	1482
LNC_0034	0.284919	0.95313	1.41873	1.267914	1.175612	1.086462	6	24	34	35	30	35	XLOC_034	lncRNA	chr3	21350751	21354441	-	4	628
LNC_0034	0.62658	1.44171	0.426472	0.148838	0.058681	0.460681	12	33	10	4	2	14	XLOC_034	lncRNA	chr3	22218062	22218645	-	1	584
LNC_0034	0.907587	1.341457	0	0.648679	0.272802	1.332403	13	22	0	12	5	29	XLOC_034	lncRNA	chr3	22477318	22478545	-	3	420
LNC_0034	0.536294	0.622895	0.780219	1.856631	1.15654	1.562124	13	18	22	61	35	58	XLOC_034	lncRNA	chr3	23671750	23674118	-	4	740
LNC_0034	7.023053	6.015882	5.563806	2.214961	2.545272	3.679717	204	208	187	86	91	163	XLOC_034	lncRNA	chr3	24165561	24167181	-	2	883
LNC_0036	1.511838	1.985295	0.750006	1.883073	3.272468	3.454106	36	56	21	59	95	124	XLOC_036	lncRNA	chr4	28687565	28688884	+	3	711
LNC_0050	2.42786	2.063277	3.386147	4.73915	1.519889	1.341666	30	30	48	77	23	25	XLOC_050	lncRNA	chr9	16839450	16839818	+	1	369
LNC_0050	1.676947	0.219885	0.963684	0.075822	0.426336	0.073574	30	5	20	2	10	3	XLOC_050	lncRNA	chr9	16942490	16943188	+	2	542
LNC_0050	1.27358	1.182879	1.369404	0.28602	0.626199	0.610474	24	27	30	8	15	18	XLOC_050	lncRNA	chr9	15043089	15043660	+	1	572
LNC_0050	1.89277	0.993049	0.385741	0.081462	0.147501	0.239518	20	13	5	2	2	4	XLOC_050	lncRNA	chr9	15505795	15506887	+	2	313
LNC_0050	0.051307	0.065443	0.286535	1.380047	1.045457	1.269814	3	4	13	73	51	76	XLOC_050	lncRNA	chr9	16156548	16159125	+	3	1190
LNC_0050	0.733395	1.276832	0.758092	1.427086	1.166204	1.148311	28	58	34	73	55	67	XLOC_050	lncRNA	chr9	16299004	16301454	+	3	1163
LNC_0050	1.382159	0.282314	0.416639	0.119705	0.375471	0.277635	20	5	7	3	7	7	XLOC_050	lncRNA	chr9	16590154	16590594	+	1	441
LNC_0050	0.551054	1.260555	1.321482	1.399514	0.969951	0.610485	9	25	25	31	20	16	XLOC_050	lncRNA	chr9	16642418	16644329	+	3	496
LNC_0043	26.73281	24.68828	18.74195	28.35419	23.39754	37.10304	650	716	529	925	703	1381	XLOC_044	lncRNA	chr6	30162946	30164264	+	4	742
LNC_0043	1.969484	1.486057	0.749258	1.154053	1.255902	1.401166	94	84	42	74	74	102	XLOC_044	lncRNA	chr6	30066860	30070856	+	8	1447
LNC_0043	2.960753	1.910129	1.578768	3.657013	3.099784	4.223352	213	164	132	352	275	464	XLOC_044	lncRNA	chr6	28149057	28153654	+	10	2189
LNC_0043	1.321594	0	0.037605	0.141331	0	0	14	0	1	2	0	0	XLOC_044	lncRNA	chr6	27835160	27835481	+	1	322
LNC_0043	3.041294	2.066054	2.236262	0.88712	1.425025	1.521267	54	43	46	21	31	41	XLOC_044	lncRNA	chr6	28546999	28547531	+	1	533
LNC_0043	3.031681	1.112208	3.064352	0.763906	1.609725	0.165123	48	21	57	17	32	4	XLOC_044	lncRNA	chr6	29007149	29008078	+	2	483
LNC_0043	0.815135	0.748612	0.439993	1.161218	2.578433	1.391361	26	29	17	50	101	68	XLOC_044	lncRNA	chr6	28960865	28962482	+	5	965
LNC_0043	0.604759	0	1.284073	0.56485	0.623539	0.501977	5	0	12	7	7	7	XLOC_044	lncRNA	chr6	29911593	29912417	+	3	246
LNC_0043	0.310449	0.440561	0.286733	2.479065	2.091041	3.545011	7	11	7	65	50	105	XLOC_044	lncRNA	chr6	29812256	29812986	+	2	590
LNC_0018	1.739017	1.335628	0.58788	0.051019	0	0.340953	26	24	10	2	0	8	XLOC_018	lncRNA	chr17	3433729	3434174	+	1	446
LNC_0018	0.480141	0.370646	0.595539	0.441152	1.289893	0.62223	22	20	31	26	70	42	XLOC_018	lncRNA	chr17	3588650	3589988	+	1	1339
LNC_0018	0.727091	2.484427	0.851363	1.064431	0.448077	0.605792	8	29	10	14	6	9	XLOC_018	lncRNA	chr17	3764202	3764668	+	2	296
LNC_0018	3.460925	2.778969	2.556464	0.337317	0.119609	0.959394	88	84	76	12	4	38	XLOC_018	lncRNA	chr17	3840704	3842533	+	6	773
LNC_0018	2.415021	3.304248	1.658307	0.424926	2.853486	0.926008	113	183	90	27	164	66	XLOC_018	lncRNA	chr17	2250365	2252154	+	3	1416
LNC_0018	0.167602	0.332333	0.242479	1.381779	1.312456	1.108251	7	16	12	74	64	67	XLOC_018	lncRNA	chr17	3335802	3337004	+	1	1203
LNC_0018	0.23205	0.403253	0.601616	2.285747	2.111877	2.418226	10	19	28	119	102	144	XLOC_018	lncRNA	chr17	3338355	3340865	+	3	1184
LNC_0018	0.788919	1.472265	0.907707	0.170004	0.132091	0.408321	9	20	13	3	2	8	XLOC_018	lncRNA	chr17	3347472	3347819	+	1	348
LNC_0018	0.27879	0.467898	0	1.246813	0	0	2	4	0	12	0	0	XLOC_019	lncRNA	chr17	4109881	4110099	+	1	219
LNC_0008	1.179468	1.047171	0.3777	0.132521	0.59381	0.450194	26	27	10	4	16	15	XLOC_009	lncRNA	chr12	28202333	28203139	+	2	648
LNC_0008	16.03748	15.28574	11.34814	11.34057	9.103259	14.36805	586	666	481	556	411	804	XLOC_009	lncRNA	chr12	27360277	27361391	+	1	1115
LNC_0008	0.802236	0.956231	0.673263	1.336303	1.807494	1.966918	34	48	33	75	94	126	XLOC_009	lncRNA	chr12	26976832	26979019	+	4	1273
LNC_0008	20.124	23.94853	15.65763	7.086174	9.045443	18.57753	240	341	217	114	134	340	XLOC_009	lncRNA	chr12	25724700	25725326	+	2	364
LNC_0008	4.390695	19.03522	1.580233	1.183265	0.938539	1.20472	102	527	43	37	27	43	XLOC_009	lncRNA	chr12	25239480	25243288	+	3	709
LNC_0008	2.618825	1.765951	1.667418	3.57054	2.468991	4.196748	86	69	64	157	100	211	XLOC_009	lncRNA	chr12	23959010	23960701	+	3	999
LNC_0025	1.501352	1.328115	0.620328	1.707197	1.952798	1.978876	24	26	12	37	39	49	XLOC_026	lncRNA	chr2	24476492	24476979	+	1	488
LNC_0025	0.695693	0.726063	0.908158	1.525835	1.213003	1.497216	37	46	56	109	80	122	XLOC_026	lncRNA	chr2	24423777	24425399	+	1	1623
LNC_0025	4.518429	4.052008	4.31581	4.798077	3.463727	3.76992	335	358	371	477	317	428	XLOC_026	lncRNA	chr2	25439301	25441560	+	1	2260
LNC_0025	0.50269	0.745973	0.647031	2.165489	0.695392	1.132202	40	70	59	227	67	135	XLOC_026	lncRNA	chr2	25365111	25368389	+	5	2377
LNC_0025	0	0.152485	0.104458	0.469835	1.569132	0.830247	0	3	2	11	32	21	XLOC_026	lncRNA	chr2	25705083	25706147	+	2	504
LNC_0025	2.906732	1.825183	2.273392	0.7497	1.117042	0.810275	107	80	97	37	51	46	XLOC_026	lncRNA	chr2	26286837	26287958	+	1	1122
LNC_0025	1.246036	0.336915	0.707824	0.754911	1.211018	1.665496	33	11	22	27	40	68	XLOC_026	lncRNA	chr2	26213779	26214759	+	2	808
LNC_0025	0	0	0	1.459019	1.440559	5.290601	0	0	0	22	20	92	XLOC_026	lncRNA	chr2	26365628	26365970	+	1	343
LNC_0025	1.870308	1.761406	1.288432	0.73208	0.920105	1.854581	50	57	40	27	31	76	XLOC_026	lncRNA	chr2	26294530	26295345	+	1	816
LNC_0015	1.611209	0.873759	0.429216	0.508132	0.552943	1.18852	50	32	16	21	21	56	XLOC_016	lncRNA	chr15	23280848	23283475	-	3	938
LNC_0015	0.634709	0.308643	0.539861	1.27936	0.843314	1.074153	11	7	11	29	17	27	XLOC_016	lncRNA	chr15	23284509	23285006	-	1	498
LNC_0015	1.356778	0.816134	1.382471	0.82743	0.501932	0.892872	45	32	52	37	21	45	XLOC_016	lncRNA	chr15	22483266	22484255	-	1	990
LNC_0015	1.258866	0	0.129658	0.045874	0	0	12	0	2	1	0	0	XLOC_016	lncRNA	chr15	22495065	22496053	-	2	291
LNC_0015	2.602145	1.687034	1.466337	2.530057	3.706201	4.287294	125	97	82	163	220	315	XLOC_016	lncRNA	chr15	22622091	22624958	-	7	1462
LNC_0015	0.335982	0.316362	0.46385	1.322825	1.852085	1.14903	10	12	17	53	69	53	XLOC_016	lncRNA	chr15	22998352	23001296	-	4	908
LNC_0015	0	0.116252	1.293084	0	0	0.13992	0	2	14	0	0	3	XLOC_016	lncRNA	chr15	22230541	22230825	-	1	285
LNC_0015	1.338925	0.102619	0.173951	0	0.207394	0	11	1	2	0	2	0	XLOC_016	lncRNA	chr15	22329627	22330339	-	2	228
LNC_0015	2.202184	1.293856	1.485464	1.590124	0.398357	1.331893	16	11	13	16	4	15	XLOC_016	lncRNA	chr15	22377929	22378145	-	1	217
LNC_0041	1.253962	0.290282	0.389757	0.204982	0.294201	0.225933	15	5	6	4	5	5	XLOC_041	lncRNA	chr5	15932236	15933010	-	2	353
LNC_0010	1.283976	0.630038	0.521234	0.305873	0.523613	0.694684	44	26	21	14	23	37	XLOC_011	lncRNA	chr13	11490258	11491867	+	3	1039
LNC_0010	1.997519	1.749845	2.262834	0.611135	0.847432	0.991492	108	112	141	44	57	82	XLOC_011	lncRNA	chr13	11607543	11609180	+	1	1638
LNC_0010	0.781645	1.104902	0.264896	2.200004	2.353336	3.460397	33	55	13	123	121	221	XLOC_011	lncRNA	chr13	10908383	10910997	+	6	1268
LNC_0010	1.69397	1.893245	2.240833	0.583795	0.307637	0.235446	98	131	150	46	22	21	XLOC_011	lncRNA	chr13	11038480	11040703	+	2	1762
LNC_0010	0.020217	1.090295	1.394622	0.291838	0.163668	0.144368	1	13	17	4	3	3	XLOC_011	lncRNA	chr13	10865226	10865527	+	1	302
LNC_0010	2.06299	2.280513	1.126532	1.949672	0.90959	2.123514	44	58	28	56	24	70	XLOC_011	lncRNA	chr13	9904873	9906357	+	4	651
LNC_0010	1.129565	1.760818	1.181964	2.976174	3.015321	4.039604	32	59	38	111	104	172	XLOC_011	lncRNA	chr13	10089751	10091506	+	3	846
LNC_0010	0.311556	1.265057	0.054886	0.049941	0	0	3	12	1	1	0	0	XLOC_011	lncRNA	chr13	11764777	11765019	+	1	243

Supplementary Table 3. A list of differentially expressed lncRNAs in *Pohlia nutans* under UV-B Radiation

ID	log2FoldChange	pvalue	padj	regulated	lncRNA_Gene_ID
LNC_003172	8.594597974	3.77E-284	9.54E-281	up	XLOC_032346
LNC_000984	7.300310245	5.71E-173	2.12E-170	up	XLOC_010524
LNC_002392	7.374146372	3.16E-162	8.99E-160	up	XLOC_024439
LNC_003273	3.877550127	1.07E-147	2.29E-145	up	XLOC_033283
LNC_001806	3.241398339	9.01E-130	1.34E-127	up	XLOC_018695
LNC_000679	-3.040796995	7.71E-86	4.36E-84	down	XLOC_007287
LNC_001428	3.613905683	2.08E-82	1.08E-80	up	XLOC_015102
LNC_000665	3.945772204	9.00E-82	4.61E-80	up	XLOC_007168
LNC_003796	6.275938071	5.51E-80	2.69E-78	up	XLOC_038826
LNC_000439	-4.380744063	1.31E-78	6.14E-77	down	XLOC_004950
LNC_001454	-4.228537731	1.12E-75	4.86E-74	down	XLOC_015352
LNC_001186	4.690768461	8.19E-73	3.32E-71	up	XLOC_012509
LNC_001741	6.138489987	3.39E-71	1.30E-69	up	XLOC_018122
LNC_002506	3.97905679	6.63E-71	2.52E-69	up	XLOC_025532
LNC_004444	-4.243044702	2.14E-70	8.08E-69	down	XLOC_045296
LNC_002715	3.84986049	1.37E-63	4.29E-62	up	XLOC_027815
LNC_000715	6.051338797	1.17E-59	3.29E-58	up	XLOC_007662
LNC_002042	-8.812394823	6.30E-59	1.72E-57	down	XLOC_020958
LNC_004733	-3.011484934	1.01E-58	2.74E-57	down	XLOC_048015
LNC_003941	5.238030408	1.21E-58	3.26E-57	up	XLOC_040151
LNC_004514	4.78464046	8.55E-57	2.17E-55	up	XLOC_045932
LNC_001520	-3.8385692	4.49E-56	1.12E-54	down	XLOC_015971
LNC_002227	3.531597072	8.07E-55	1.92E-53	up	XLOC_022686
LNC_000718	5.886043786	1.61E-54	3.81E-53	up	XLOC_007667
LNC_002481	3.683704667	2.06E-54	4.85E-53	up	XLOC_025269
LNC_000466	5.00622334	8.39E-53	1.88E-51	up	XLOC_005167
LNC_003296	3.739834407	1.14E-51	2.48E-50	up	XLOC_033521
LNC_003601	5.742146793	2.70E-50	5.64E-49	up	XLOC_036648
LNC_004335	3.505597601	1.42E-48	2.82E-47	up	XLOC_044145
LNC_002281	-4.937043491	1.04E-47	2.01E-46	down	XLOC_023243
LNC_003352	7.552862111	1.10E-47	2.13E-46	up	XLOC_034114
LNC_003314	3.448624302	1.06E-45	1.94E-44	up	XLOC_033661
LNC_000780	6.577215684	1.23E-45	2.23E-44	up	XLOC_008359
LNC_004822	-2.973389278	1.80E-45	3.25E-44	down	XLOC_048817
LNC_000539	-3.180771367	7.92E-44	1.35E-42	down	XLOC_005785
LNC_001281	2.852368679	1.62E-42	2.64E-41	up	XLOC_013565
LNC_000709	-4.656591023	2.46E-42	3.98E-41	down	XLOC_007596
LNC_003321	3.26609732	5.09E-42	8.13E-41	up	XLOC_033738
LNC_002341	2.474319633	1.30E-40	1.98E-39	up	XLOC_023937
LNC_003257	4.166330426	3.64E-40	5.50E-39	up	XLOC_033133
LNC_002115	-3.338334193	5.01E-39	7.28E-38	down	XLOC_021594
LNC_000402	4.790027474	8.78E-39	1.27E-37	up	XLOC_004461
LNC_002592	-2.733944368	1.19E-37	1.66E-36	down	XLOC_026456
LNC_004881	2.14009494	2.74E-37	3.76E-36	up	XLOC_049352
LNC_003275	-4.875099534	3.66E-37	5.00E-36	down	XLOC_033294
LNC_001093	-3.38093421	1.58E-36	2.11E-35	down	XLOC_011759
LNC_004257	-4.489619315	3.87E-35	4.90E-34	down	XLOC_043294
LNC_003038	7.304966236	3.99E-34	4.87E-33	up	XLOC_031096
LNC_002472	5.115168229	5.11E-34	6.23E-33	up	XLOC_025203
LNC_002838	3.483796141	2.73E-33	3.24E-32	up	XLOC_029048
LNC_000797	-2.396628779	1.81E-32	2.08E-31	down	XLOC_008533
LNC_002787	5.095733014	2.23E-32	2.56E-31	up	XLOC_028560
LNC_002454	3.182125442	2.70E-32	3.09E-31	up	XLOC_025039
LNC_003794	-4.838310648	2.86E-32	3.27E-31	down	XLOC_038778
LNC_005087	5.046415023	5.99E-32	6.75E-31	up	XLOC_051270
LNC_003566	1.698097504	7.16E-32	8.05E-31	up	XLOC_036319
LNC_002802	4.681827516	2.31E-31	2.55E-30	up	XLOC_028698
LNC_004467	5.421367743	4.19E-31	4.56E-30	up	XLOC_045505
LNC_002117	-3.744598822	6.19E-31	6.69E-30	down	XLOC_021594
LNC_005350	-1.577188992	6.94E-31	7.49E-30	down	XLOC_053456
LNC_002426	3.062050802	1.52E-30	1.62E-29	up	XLOC_024786
LNC_001069	2.946094109	1.69E-30	1.80E-29	up	XLOC_011471
LNC_004096	3.15641308	3.27E-30	3.43E-29	up	XLOC_041696
LNC_005050	4.545949203	3.74E-30	3.92E-29	up	XLOC_050869
LNC_001495	2.770115497	6.06E-30	6.29E-29	up	XLOC_015798
LNC_000999	2.349107073	8.32E-30	8.57E-29	up	XLOC_010723
LNC_000272	4.139007525	1.39E-29	1.42E-28	up	XLOC_003129
LNC_004591	2.280759989	2.96E-29	2.98E-28	up	XLOC_046582
LNC_000302	3.521778986	3.64E-29	3.65E-28	up	XLOC_003370
LNC_001302	4.541552788	4.54E-29	4.55E-28	up	XLOC_013725
LNC_000016	-2.33065063	9.18E-29	9.07E-28	down	XLOC_000213
LNC_000522	-4.240367389	1.57E-28	1.53E-27	down	XLOC_005606
LNC_001215	-4.272310582	5.20E-28	4.97E-27	down	XLOC_012807
LNC_005041	-3.99437078	7.77E-28	7.37E-27	down	XLOC_050818
LNC_002398	7.310645326	2.17E-27	2.02E-26	up	XLOC_024454
LNC_004579	-2.176979452	2.48E-27	2.31E-26	down	XLOC_046489
LNC_001747	-3.287346188	3.22E-27	2.98E-26	down	XLOC_018143
LNC_000105	2.022085916	3.45E-27	3.19E-26	up	XLOC_001113
LNC_004934	2.86411343	2.46E-26	2.19E-25	up	XLOC_049750
LNC_003811	-2.244345307	2.56E-26	2.28E-25	down	XLOC_038993
LNC_003603	3.648744743	9.41E-26	8.15E-25	up	XLOC_036651
LNC_003246	2.201755704	1.37E-25	1.18E-24	up	XLOC_032942
LNC_004307	2.533432507	1.65E-25	1.41E-24	up	XLOC_043818
LNC_003262	-3.14688016	4.88E-25	4.09E-24	down	XLOC_033151
LNC_004246	2.942837893	6.59E-25	5.49E-24	up	XLOC_043182
LNC_003907	2.143436315	7.74E-25	6.43E-24	up	XLOC_039889
LNC_002151	3.90806525	1.91E-24	1.56E-23	up	XLOC_021904
LNC_001726	2.595991494	3.51E-24	2.83E-23	up	XLOC_017966
LNC_003058	3.995236422	4.03E-24	3.24E-23	up	XLOC_031263
LNC_000818	6.89734081	4.71E-24	3.78E-23	up	XLOC_008776
LNC_001661	1.925729899	4.81E-24	3.85E-23	up	XLOC_017434
LNC_002563	3.086720142	5.79E-24	4.62E-23	up	XLOC_026148
LNC_004713	1.620058417	1.15E-23	9.04E-23	up	XLOC_047872
LNC_002488	-2.209829135	1.84E-23	1.44E-22	down	XLOC_025368
LNC_001640	1.649871978	2.19E-23	1.71E-22	up	XLOC_017214
LNC_004659	3.009383684	2.56E-23	1.99E-22	up	XLOC_047143
LNC_003209	1.957454623	3.54E-23	2.73E-22	up	XLOC_032606
LNC_002869	-2.447516027	4.89E-23	3.75E-22	down	XLOC_029391
LNC_000246	2.803710408	6.10E-23	4.65E-22	up	XLOC_002792
LNC_004085	4.373430456	2.00E-22	1.49E-21	up	XLOC_041551
LNC_004113	5.244797824	2.20E-22	1.63E-21	up	XLOC_041822
LNC_004937	1.7322582	3.02E-22	2.23E-21	up	XLOC_049768
LNC_004947	3.849593176	3.43E-22	2.53E-21	up	XLOC_049860
LNC_005294	-1.226183629	4.11E-22	3.01E-21	down	XLOC_052996
LNC_001970	-5.040199597	4.84E-22	3.53E-21	down	XLOC_020313
LNC_005064	2.134479444	1.13E-21	8.09E-21	up	XLOC_051035
LNC_004519	2.802471619	1.95E-21	1.38E-20	up	XLOC_045975
LNC_002790	4.505111823	2.08E-21	1.47E-20	up	XLOC_028602
LNC_001385	5.147404681	2.40E-21	1.69E-20	up	XLOC_014689
LNC_003125	1.398002902	3.59E-21	2.51E-20	up	XLOC_031860
LNC_004294	-5.03817676	4.58E-21	3.18E-20	down	XLOC_043735
LNC_004581	1.199256273	7.70E-21	5.29E-20	up	XLOC_046498
LNC_003463	-1.980763661	7.85E-21	5.39E-20	down	XLOC_035391
LNC_003334	2.82986542	9.33E-21	6.38E-20	up	XLOC_033819
LNC_000694	4.743681522	9.64E-21	6.59E-20	up	XLOC_007423
LNC_000095	4.221509173	1.07E-20	7.30E-20	up	XLOC_001002
LNC_000003	-2.273496992	1.07E-20	7.32E-20	down	XLOC_000038
LNC_002318	4.103595151	1.20E-20	8.13E-20	up	XLOC_023611
LNC_004805	-3.020471618	1.36E-20	9.19E-20	down	XLOC_048627
LNC_004966	-3.779156007	1.45E-20	9.79E-20	down	XLOC_050026
LNC_000413	4.206901712	2.61E-20	1.74E-19	up	XLOC_004616
LNC_000840	2.805110583	3.56E-20	2.36E-19	up	XLOC_009008
LNC_002489	-2.423739177	3.97E-20	2.63E-19	down	XLOC_025369
LNC_004214	4.289490763	4.44E-20	2.93E-19	up	XLOC_042841
LNC_004277	3.798558733	5.04E-20	3.31E-19	up	XLOC_043533
LNC_000739	2.631566251	5.16E-20	3.39E-19	up	XLOC_007900
LNC_000582	3.782614663	5.26E-20	3.45E-19	up	XLOC_006263
LNC_002395	8.02808918	5.78E-20	3.79E-19	up	XLOC_024446
LNC_002093	2.723690291	8.48E-20	5.51E-19	up	XLOC_021420
LNC_000558	2.7231327	1.01E-19	6.53E-19	up	XLOC_006033
LNC_005072	4.222542171	1.05E-19	6.79E-19	up	XLOC_051109
LNC_000135	11.25489735	1.19E-19	7.69E-19	up	XLOC_001475
LNC_000809	2.394551153	4.24E-19	2.66E-18	up	XLOC_008683
LNC_001705	2.012809232	4.30E-19	2.70E-18	up	XLOC_017757
LNC_003873	3.086916405	4.55E-19	2.85E-18	up	XLOC_039473
LNC_002542	-5.866525957	4.77E-19	2.98E-18	down	XLOC_025940
LNC_002230	-2.999958011	5.75E-19	3.59E-18	down	XLOC_022713
LNC_000139	3.18081046	5.93E-19	3.69E-18	up	XLOC_001498
LNC_000336	-3.814823552	7.07E-19	4.39E-18	down	XLOC_003739
LNC_003701	10.69518759	7.09E-19	4.40E-18	up	XLOC_037752
LNC_003199	3.609301835	7.29E-19	4.52E-18	up	XLOC_032532
LNC_001596	-3.88662812	9.26E-19	5.71E-18	down	XLOC_016711
LNC_005116	6.354010584	1.03E-18	6.37E-18	up	XLOC_051515
LNC_004372	-2.272979294	1.09E-18	6.71E-18	down	XLOC_044611
LNC_004657	4.737714767	1.10E-18	6.77E-18	up	XLOC_047131
LNC_000776	4.749969632	1.41E-18	8.62E-18	up	XLOC_008306
LNC_002610	-4.743891329	2.32E-18	1.40E-17	down	XLOC_026580
LNC_002430	-1.450323139	2.58E-18	1.55E-17	down	XLOC_024813
LNC_004885	-2.660732834	2.83E-18	1.70E-17	down	XLOC_049388
LNC_003046	1.920431145	2.99E-18	1.79E-17	up	XLOC_031180
LNC_004039	4.72732738	3.16E-18	1.89E-17	up	XLOC_041163
LNC_002593	-2.114421996	3.61E-18	2.15E-17	down	XLOC_026457
LNC_005049	6.992295741	3.74E-18	2.23E-17	up	XLOC_050861
LNC_001122	-5.626813953	5.48E-18	3.24E-17	down	XLOC_011916
LNC_004917	-2.220213636	7.17E-18	4.21E-17	down	XLOC_049626
LNC_004289	3.589787673	7.61E-18	4.46E-17	up	XLOC_043663
LNC_002839	-6.913641549	9.62E-18	5.60E-17	down	XLOC_029057
LNC_002861	-3.099057947	1.02E-17	5.93E-17	down	XLOC_029246
LNC_003845	4.287409061	1.21E-17	6.99E-17	up	XLOC_039285
LNC_003034	-3.641854446	1.25E-17	7.21E-17	down	XLOC_031070
LNC_004041	4.308091927	1.30E-17	7.52E-17	up	XLOC_041174
LNC_002122	10.3758911	1.78E-17	1.02E-16	up	XLOC_021638
LNC_002146	-2.598482356	1.90E-17	1.09E-16	down	XLOC_021861
LNC_003251	-4.215866123	1.94E-17	1.11E-16	down	XLOC_032985
LNC_000417	1.739344435	2.41E-17	1.37E-16	up	XLOC_004667
LNC_004213	2.057467164	2.75E-17	1.56E-16	up	XLOC_042836
LNC_000608	-1.733757368	4.90E-17	2.74E-16	down	XLOC_006536
LNC_000386	-2.598533652	5.78E-17	3.21E-16	down	XLOC_004261
LNC_005454	-1.738176314	8.12E-17	4.47E-16	down	XLOC_054349
LNC_004398	5.036068736	1.00E-16	5.50E-16	up	XLOC_044842
LNC_004003	2.54553501	1.14E-16	6.24E-16	up	XLOC_040702
LNC_004260	2.464021211	1.43E-16	7.74E-16	up	XLOC_043334
LNC_002824	3.403753064	1.98E-16	1.07E-15	up	XLOC_028882
LNC_004391	-1.392762035	2.13E-16	1.14E-15	down	XLOC_044742
LNC_002711	-5.237752961	3.42E-16	1.81E-15	down	XLOC_027790
LNC_003036	7.393418135	3.55E-16	1.88E-15	up	XLOC_031090
LNC_004634	2.306320867	3.62E-16	1.91E-15	up	XLOC_046938
LNC_002665	-2.892812147	3.96E-16	2.09E-15	down	XLOC_027132
LNC_000571	-3.558887964	4.94E-16	2.59E-15	down	XLOC_006139
LNC_000311	4.785411137	7.58E-16	3.93E-15	up	XLOC_003484
LNC_004717	3.311229892	8.47E-16	4.38E-15	up	XLOC_047893
LNC_003785	3.225687669	8.70E-16	4.49E-15	up	XLOC_038675
LNC_002359	-3.066324895	9.45E-16	4.87E-15	down	XLOC_024039
LNC_000351	1.887469506	9.74E-16	5.02E-15	up	XLOC_003824
LNC_001921	-2.496387572	1.07E-15	5.50E-15	down	XLOC_019882
LNC_000987	3.248821668	1.12E-15	5.74E-15	up	XLOC_010571
LNC_000125	2.082328194	1.45E-15	7.41E-15	up	XLOC_001320
LNC_002759	2.884175925	1.55E-15	7.90E-15	up	XLOC_028229
LNC_002795	-1.846100955	1.58E-15	8.03E-15	down	XLOC_028629
LNC_004431	-1.857283361	1.64E-15	8.33E-15	down	XLOC_045169
LNC_001549	4.568711592	2.04E-15	1.03E-14	up	XLOC_016228
LNC_002033	4.026934322	2.25E-15	1.13E-14	up	XLOC_020861
LNC_002224	-5.310619114	2.27E-15	1.14E-14	down	XLOC_022646
LNC_001277	2.930718581	2.36E-15	1.19E-14	up	XLOC_013527
LNC_003476	2.358644866	2.45E-15	1.23E-14	up	XLOC_035471
LNC_004883	1.84273946	3.31E-15	1.65E-14	up	XLOC_049367
LNC_002775	-3.829848429	3.59E-15	1.79E-14	down	XLOC_028426
LNC_003984	3.646563194	3.63E-15	1.80E-14	up	XLOC_040539
LNC_004506	1.232012622	3.81E-15	1.89E-14	up	XLOC_045886
LNC_001283	-6.58283092	3.87E-15	1.92E-14	down	XLOC_013577
LNC_000666	9.845075405	4.07E-15	2.02E-14	up	XLOC_007168
LNC_003841	4.11267739	4.85E-15	2.39E-14	up	XLOC_039226
LNC_003814	-4.179697969	4.91E-15	2.42E-14	down	XLOC_039024
LNC_001792	-3.51087414	5.68E-15	2.79E-14	down	XLOC_018581
LNC_000350	-2.459743065	6.05E-15	2.96E-14	down	XLOC_003822
LNC_004053	3.199385952	7.32E-15	3.57E-14	up	XLOC_041290
LNC_004769	2.170644094	8.15E-15	3.96E-14	up	XLOC_048277
LNC_004873	2.149331687	9.34E-15	4.52E-14	up	XLOC_049251
LNC_000200	-2.353857841	9.69E-15	4.69E-14	down	XLOC_002207
LNC_003878	-2.723530748	1.04E-14	5.04E-14	down	XLOC_039556

Supplementary Table 4. Representative UV-B radiation-related genes of *Pohlia nutans* .

Flavonoid biosynthesis

lncRNA ID	og2FoldChange	Cis-Target Gene ID	og2FoldChang	gene name	NR	SWISS
LNC_000020	-1.499699239	Novel_000522	-2.47007881	ANS	REDICTED: probable 2-oxoglutarate/Fe(II)-dependent dioxygenase [Ziziphus jujub	-oxoglutarate-dependent dioxygenase ANS {ECO:0000305}; EC=1.14.11.- {ECO:0000305}; AltName: Fu
LNC_000020	-1.499699239	rna-Poh0344750.1	-3.310716341	ANS	REDICTED: probable 2-oxoglutarate/Fe(II)-dependent dioxygenase [Ziziphus jujub	-oxoglutarate-dependent dioxygenase ANS {ECO:0000305}; EC=1.14.11.- {ECO:0000305}; AltName: Fu
LNC_000020	-1.499699239	rna-Poh0344760.1	-1.864352269	ANS	REDICTED: probable 2-oxoglutarate/Fe(II)-dependent dioxygenase [Ziziphus jujub	-oxoglutarate-dependent dioxygenase ANS {ECO:0000305}; EC=1.14.11.- {ECO:0000305}; AltName: Fu
LNC_000155	1.463222107	rna-Poh0049790.1	#N/A	CHS	predicted protein [Physcomitrella patens]	7_PICMA RecName: Full=Chalcone synthase 7; EC=2.3.1.74; AltName: Full=Naregenin-chalcone syntha
LNC_000155	1.463222107	rna-Poh0049800.1	#N/A	CHS	predicted protein [Physcomitrella patens]	IPOBA RecName: Full=Chalcone synthase LF2; EC=2.3.1.74; AltName: Full=Naringenin-chalcone syntha
LNC_000244	1.854160615	Novel_000122	2.35223975	CHS	predicted protein [Physcomitrella patens]	5_SOYBN RecName: Full=Chalcone synthase 6; EC=2.3.1.74; AltName: Full=Naringenin-chalcone syntha
LNC_000244	1.854160615	Novel_000623	2.105214053	CHS	predicted protein [Physcomitrella patens]	5_SOYBN RecName: Full=Chalcone synthase 6; EC=2.3.1.74; AltName: Full=Naringenin-chalcone syntha
LNC_000244	1.854160615	rna-Poh0271480.1	1.941038573	CHS	predicted protein [Physcomitrella patens]	5_SOYBN RecName: Full=Chalcone synthase 6; EC=2.3.1.74; AltName: Full=Naringenin-chalcone syntha
LNC_000244	1.854160615	rna-Poh0271490.1	2.089060974	CHS	predicted protein [Physcomitrella patens]	5_SOYBN RecName: Full=Chalcone synthase 6; EC=2.3.1.74; AltName: Full=Naringenin-chalcone syntha
LNC_000251	1.03941637	rna-Poh0290050.1	-2.728607226	C3'H	hypothetical protein PHYPA_027326 [Physcomitrella patens]	C98A2_SOYBN RecName: Full=Cytochrome P450 98A2; EC=1.14.-.-;
LNC_000398	2.180686764	rna-Poh0074370.1	#N/A	C4H	predicted protein [Physcomitrella patens]	ıse; EC=1.14.13.11; AltName: Full=Cinnamic acid 4-hydroxylase; Short=C4H; Short=CA4H; AltName: F
LNC_000418	4.239131536	rna-Poh0078400.1	#N/A	CcoAOMT	predicted protein [Physcomitrella patens]	ɔyl-CoA O-methyltransferase; EC=2.1.1.104; AltName: Full=Trans-caffeoyl-CoA 3-O-methyltransferase; S
LNC_000529	-2.484535249	rna-Poh0080580.1	-1.792338844	F3'H	flavonoid 3'-hydroxylase [Pohlia nutans]	C7A12_PANGI RecName: Full=Cytochrome P450 CYP736A12; EC=1.14.-.-;
LNC_000529	-2.484535249	Novel_001091	#N/A	F3'H	flavonoid 3'-hydroxylase [Pohlia nutans]	C71DB_LOTJA RecName: Full=Cytochrome P450 71D11; EC=1.14.-.-; Flags: Fragment;
LNC_000622	3.812817347	rna-Poh0123620.1	2.487164472	C4H	hypothetical protein PHYPA_029802 [Physcomitrella patens]	ase; EC=1.14.13.11; AltName: Full=Cinnamic acid 4-hydroxylase; Short=C4H; Short=CA4H; AltName: F
LNC_000689	1.153835079	rna-Poh0315720.1	-0.929086226	CcoAOMT	predicted protein [Physcomitrella patens]	l-CoA O-methyltransferase 2; EC=2.1.1.104; AltName: Full=Trans-caffeoyl-CoA 3-O-methyltransferase 2;
LNC_000809	2.394551153	rna-Poh0143650.1	-0.050591531	CcoAOMT	predicted protein [Physcomitrella patens]	l-CoA O-methyltransferase 2; EC=2.1.1.104; AltName: Full=Trans-caffeoyl-CoA 3-O-methyltransferase 2;
LNC_000980	3.755980556	rna-Poh0230040.1	-0.761410306	C4H	hypothetical protein PHYPA_004588 [Physcomitrella patens]	ase; EC=1.14.13.11; AltName: Full=Cinnamic acid 4-hydroxylase; Short=C4H; Short=CA4H; AltName: F
LNC_001001	1.9759239	Novel_002147	1.9579021	C4H	hypothetical protein PHYPA_006574 [Physcomitrella patens]	ıse; EC=1.14.13.11; AltName: Full=Cinnamic acid 4-hydroxylase; Short=C4H; Short=CA4H; AltName: F
LNC_001001	1.9759239	rna-Poh0326440.1	1.760103233	C4H	hypothetical protein PHYPA_006574 [Physcomitrella patens]	ıse; EC=1.14.13.11; AltName: Full=Cinnamic acid 4-hydroxylase; Short=C4H; Short=CA4H; AltName: F
LNC_001069	2.946094109	rna-Poh0312170.1	-0.824379979	F3'H	flavonoid 3',5'-hydroxylase [Pohlia nutans]	ull=Flavonoid 3'-monooxygenase; EC=1.14.13.21; AltName: Full=Cytochrome P450 75B2; AltName: Ful
LNC_001260	-1.032671498	rna-Poh0063890.1	0.309661915	F3'H	flavonoid 3',5'-hydroxylase [Pohlia nutans]	ull=Flavonoid 3'-monooxygenase; EC=1.14.13.21; AltName: Full=Cytochrome P450 75B2; AltName: Ful
LNC_001316	3.160743661	rna-Poh0059250.1	-6.90790471	HCT	hypothetical protein PHYPA_030851 [Physcomitrella patens]	0000305}; Short=OsHCT1 {ECO:0000303|PubMed:22285622}; EC=2.3.1.- {ECO:0000305}; AltName: F
LNC_001316	3.160743661	Novel_003147	1.60142105	HCT	hypothetical protein PHYPA_030849 [Physcomitrella patens]	0000305}; Short=OsHCT2 {ECO:0000303|PubMed:22285622}; EC=2.3.1.- {ECO:0000305}; AltName: F
LNC_001316	3.160743661	rna-Poh0059230.1	1.564839917	HCT	hypothetical protein PHYPA_030849 [Physcomitrella patens]	0000305}; Short=OsHCT2 {ECO:0000303|PubMed:22285622}; EC=2.3.1.- {ECO:0000305}; AltName: F
LNC_001811	1.382143228	Novel_004227	1.172917909	C4H	predicted protein [Physcomitrella patens]	ıse; EC=1.14.13.11; AltName: Full=Cinnamic acid 4-hydroxylase; Short=C4H; Short=CA4H; AltName: F
LNC_002098	-1.226777525	rna-Poh0030800.1	-0.877614842	HCT	hypothetical protein PHYPA_003798 [Physcomitrella patens]	ansferase; EC=2.3.1.133; AltName: Full=Hydroxycinnamoyl transferase; AltName: Full=Hydroxycinnamo
LNC_002231	2.566531209	rna-Poh0032630.1	-0.12198502	FLS	predicted protein [Physcomitrella patens]	IOX1_ARATH RecName: Full=Probable 2-oxoglutarate-dependent dioxygenase At3g49630; EC=1.14.11.
LNC_002275	-1.935859751	rna-Poh0039230.1	-0.322883405	CHI	chalcone isomerase 3a, partial [Conocephalum conicum]	'atty-acid-binding protein 3, chloroplastic; Short=AtFAP3; AltName: Full=Chalcone-flavanone isomerase f
LNC_002393	2.681343788	rna-Poh0318490.1	#N/A	CHI	predicted protein [Physcomitrella patens]	: Full=Vacuolar-sorting receptor 1; AltName: Full=80 kDa proaleurein-binding protein; AltName: Full=BI
LNC_002405	-2.402030503	rna-Poh0319680.1	3.044396065	F3'H	flavonoid 3'-hydroxylase [Pohlia nutans]	'ubMed:18726614}; AltName: Full=Cytochrome P450 75B3 {ECO:0000303|PubMed:26082402}; AltNan
LNC_002411	5.337536121	rna-Poh0276430.1	-5.797778871	F3'H	flavonoid 3'-hydroxylase [Pohlia nutans]	3|PubMed:17496121}; EC=1.14.13.206 {ECO:0000269|PubMed:17496121}; AltName: Full=Protein DEF
LNC_002724	4.876288626	Novel_005758	-0.695784654	CHI	--	--
LNC_002724	4.876288626	Novel_006346	0.82924894	F3'H	flavonoid 3'-hydroxylase [Pohlia nutans]	EC=1.14.13.21; AltName: Full=Cytochrome P450 75B1; AltName: Full=Flavonoid 3'-hydroxylase; Short
LNC_002724	4.876288626	rna-Poh0155260.1	0.728847682	F3'H	flavonoid 3'-hydroxylase [Pohlia nutans]	EC=1.14.13.21; AltName: Full=Cytochrome P450 75B1; AltName: Full=Flavonoid 3'-hydroxylase; Short
LNC_002756	-2.214481342	Novel_005803	1.786542134	CHS	predicted protein [Physcomitrella patens]	5_SOYBN RecName: Full=Chalcone synthase 6; EC=2.3.1.74; AltName: Full=Naringenin-chalcone syntha
LNC_002756	-2.214481342	Novel_005804	1.367604935	CHS	predicted protein [Physcomitrella patens]	5_SOYBN RecName: Full=Chalcone synthase 6; EC=2.3.1.74; AltName: Full=Naringenin-chalcone syntha
LNC_002756	-2.214481342	rna-Poh0096230.1	1.171844252	CHS	predicted protein [Physcomitrella patens]	5_SOYBN RecName: Full=Chalcone synthase 6; EC=2.3.1.74; AltName: Full=Naringenin-chalcone syntha
LNC_002756	-2.214481342	Novel_005805	0.105668892	CHS	predicted protein [Physcomitrella patens]	SY_ARAAL RecName: Full=Chalcone synthase; EC=2.3.1.74; AltName: Full=Naringenin-chalcone synth
LNC_002765	1.738584745	rna-Poh0094960.1	-2.961633561	C3'H	hypothetical protein PHYPA_027326 [Physcomitrella patens]	C98A2_SOYBN RecName: Full=Cytochrome P450 98A2; EC=1.14.-.-;
LNC_002773	8.055508947	Novel_005859	7.36540597	F3'5'H	predicted protein [Physcomitrella patens]	ME RecName: Full=Flavonoid 3',5'-hydroxylase; Short=F3'5'H; EC=1.14.13.88; AltName: Full=Cytochron
LNC_002773	8.055508947	rna-Poh0093310.1	0.060563243	F3'H	hypothetical protein PHYPA_004982 [Physcomitrella patens]	EC=1.14.13.21; AltName: Full=Cytochrome P450 75B1; AltName: Full=Flavonoid 3'-hydroxylase; Short
LNC_002773	8.055508947	Novel_006435	6.484424572	F3'5'H	predicted protein [Physcomitrella patens]	ME RecName: Full=Flavonoid 3',5'-hydroxylase; Short=F3'5'H; EC=1.14.13.88; AltName: Full=Cytochron
LNC_002856	1.856045412	rna-Poh0218510.1	#N/A	CHS	predicted protein [Physcomitrella patens]	7_PICMA RecName: Full=Chalcone synthase 7; EC=2.3.1.74; AltName: Full=Naregenin-chalcone syntha
LNC_002856	1.856045412	rna-Poh0218520.1	#N/A	CHS	predicted protein [Physcomitrella patens]	SY_EQUAR RecName: Full=Chalcone synthase; EC=2.3.1.74; AltName: Full=Naringenin-chalcone synth
LNC_002871	-1.701567438	rna-Poh0215440.1	#N/A	F3'H	flavonoid 3',5'-hydroxylase [Pohlia nutans]	C93A2_SOYBN RecName: Full=Cytochrome P450 93A2; EC=1.14.-.-;
LNC_002984	3.196599152	rna-Poh0164690.1	0.849379061	DRF	tetraketide alpha-pyrone reductase 1 [Quercus suber]	'ubMed:14725861}; EC=1.3.1.77 {ECO:0000269|PubMed:12532018, ECO:0000269|PubMed:14725861};
LNC_003044	1.239175945	rna-Poh0357450.1	#N/A	F3'H	flavonoid 3'-hydroxylase [Pohlia nutans]	ull=Flavonoid 3'-monooxygenase; EC=1.14.13.21; AltName: Full=Cytochrome P450 75B2; AltName: Ful
LNC_003220	1.676914657	rna-Poh0109810.1	-0.603466964	CHS	hypothetical protein PHYPA_014791 [Physcomitrella patens]	SY_CATRO RecName: Full=Chalcone synthase; EC=2.3.1.74; AltName: Full=Naringenin-chalcone synth
LNC_003333	1.320435394	rna-Poh0029570.1	2.191947606	HCT	hypothetical protein PHYPA_010928 [Physcomitrella patens]	ansferase; EC=2.3.1.133; AltName: Full=Hydroxycinnamoyl transferase; AltName: Full=Hydroxycinnamo
LNC_003359	2.146619075	rna-Poh0114330.1	#N/A	F3'H	flavonoid 3',5'-hydroxylase [Pohlia nutans]	ERAE RecName: Full=Cytochrome P450 71A1; EC=1.14.-.-; AltName: Full=ARP-2; AltName: Full=CYP
LNC_003413	-2.961453943	Novel_007824	-1.294589409	CHI	hypothetical protein PHYPA_003648 [Physcomitrella patens]	: Full=Vacuolar-sorting receptor 1; AltName: Full=80 kDa proaleurein-binding protein; AltName: Full=BI
LNC_003413	-2.961453943	rna-Poh0018880.1	-1.401661653	CHI	hypothetical protein PHYPA_003648 [Physcomitrella patens]	: Full=Vacuolar-sorting receptor 1; AltName: Full=80 kDa proaleurein-binding protein; AltName: Full=BI
LNC_003436	-5.248476243	rna-Poh0023920.1	0.916500815	HCT	predicted protein [Physcomitrella patens]	ansferase; EC=2.3.1.133; AltName: Full=Hydroxycinnamoyl transferase; AltName: Full=Hydroxycinnamo
LNC_003464	2.661415322	Novel_007560	-0.305350627	F3'5'H	flavonoid 3',5'-hydroxylase [Pohlia nutans]	ll=Flavonoid 3',5'-hydroxylase 1; Short=F3'5'H; EC=1.14.13.88; AltName: Full=CYPLXXVA1; AltName:
LNC_003464	2.661415322	rna-Poh0027220.1	-2.478042237	HCT	hypothetical protein PHYPA_007405 [Physcomitrella patens]	ubMed:27354554}; Short=OsAHT1 {ECO:0000303|PubMed:27354554}; EC=2.3.1.- {ECO:0000305}; A'
LNC_003464	2.661415322	Novel_007970	-0.468931856	HCT	hypothetical protein PHYPA_007405 [Physcomitrella patens]	ubMed:27354554}; Short=OsAHT1 {ECO:0000303|PubMed:27354554}; EC=2.3.1.- {ECO:0000305}; A'
LNC_003464	2.661415322	Novel_007561	-1.606485223	F3'5'H	flavonoid 3',5'-hydroxylase [Pohlia nutans]	ll=Flavonoid 3',5'-hydroxylase 1; Short=F3'5'H; EC=1.14.13.88; AltName: Full=CYPLXXVA1; AltName:
LNC_003670	1.181956202	rna-Poh0365170.1	2.029139315	HCT	hypothetical protein PHYPA_010928 [Physcomitrella patens]	ansferase; EC=2.3.1.133; AltName: Full=Hydroxycinnamoyl transferase; AltName: Full=Hydroxycinnamo
LNC_003677	1.904324223	rna-Poh0015390.1	3.286885905	C4H	hypothetical protein PHYPA_029802 [Physcomitrella patens]	ıse; EC=1.14.13.11; AltName: Full=Cinnamic acid 4-hydroxylase; Short=C4H; Short=CA4H; AltName: F
LNC_003729	-1.560285951	rna-Poh0006720.1	#N/A	C4H	predicted protein [Physcomitrella patens]	ıse; EC=1.14.13.11; AltName: Full=Cinnamic acid 4-hydroxylase; Short=C4H; Short=CA4H; AltName: F
LNC_003738	1.414466247	rna-Poh0005680.1	#N/A	C4H	predicted protein [Physcomitrella patens]	ıse; EC=1.14.13.11; AltName: Full=Cinnamic acid 4-hydroxylase; Short=C4H; Short=CA4H; AltName: F
LNC_003794	-4.838310648	Novel_008301	1.712341439	HCT	hypothetical protein PHYPA_030849 [Physcomitrella patens]	ansferase; EC=2.3.1.133; AltName: Full=Hydroxycinnamoyl transferase; AltName: Full=Hydroxycinnamo
LNC_003794	-4.838310648	rna-Poh0281260.1	1.21236388	HCT	hypothetical protein PHYPA_030849 [Physcomitrella patens]	ansferase; EC=2.3.1.133; AltName: Full=Hydroxycinnamoyl transferase; AltName: Full=Hydroxycinnamo
LNC_003811	-2.244345307	rna-Poh0284650.1	#N/A	F3'H	flavonoid 3',5'-hydroxylase [Pohlia nutans]	ll=Flavonoid 3',5'-hydroxylase 1; Short=F3'5'H; EC=1.14.13.88; AltName: Full=CYPLXXVA1; AltName:
LNC_004002	4.866487645	Novel_009172	-0.688473962	CHS	hypothetical protein PHYPA_019382 [Physcomitrella patens]	--
LNC_004002	4.866487645	rna-Poh0182320.1	-0.380595599	CHS	hypothetical protein PHYPA_019382 [Physcomitrella patens]	--
LNC_004083	-2.840762389	rna-Poh0168260.1	-1.558198439	CHI	hypothetical protein PHYPA_014139 [Physcomitrella patens]	'atty-acid-binding protein 3, chloroplastic; Short=AtFAP3; AltName: Full=Chalcone-flavanone isomerase f
LNC_004098	3.948837617	Novel_008983	-1.605521941	C4H	cinnamate 4-hydroxylase [Physcomitrella patens]	ıse; EC=1.14.13.11; AltName: Full=Cinnamic acid 4-hydroxylase; Short=C4H; Short=CA4H; AltName: F
LNC_004098	3.948837617	rna-Poh0170780.1	-2.45934508	C4H	cinnamate 4-hydroxylase [Physcomitrella patens]	ıse; EC=1.14.13.11; AltName: Full=Cinnamic acid 4-hydroxylase; Short=C4H; Short=CA4H; AltName: F
LNC_004152	1.375907304	rna-Poh0188230.1	#N/A	F3'H	flavonoid 3'-hydroxylase [Pohlia nutans]	ull=Flavonoid 3'-monooxygenase; EC=1.14.13.21; AltName: Full=Cytochrome P450 75B2; AltName: Ful
LNC_004152	1.375907304	Novel_009092	1.079270401	HCT	predicted protein [Physcomitrella patens]	--
LNC_004152	1.375907304	rna-Poh0188170.1	0.658133915	HCT	predicted protein [Physcomitrella patens]	--
LNC_004260	2.464021211	rna-Poh0135180.1	-1.438589404	FLS	ɔICTED: probable 2-oxoglutarate-dependent dioxygenase At5g05600 [Elaeis guine	-oxoglutarate-dependent dioxygenase ANS {ECO:0000305}; EC=1.14.11.- {ECO:0000305}; AltName: Fu
LNC_004268	-1.852867345	rna-Poh0136220.1	-1.504432824	CHI	hypothetical protein PHYPA_014139 [Physcomitrella patens]	'atty-acid-binding protein 3, chloroplastic; Short=AtFAP3; AltName: Full=Chalcone-flavanone isomerase f
LNC_004359	4.390921308	Novel_010025	-0.945314225	CHS	hypothetical protein PHYPA_019382 [Physcomitrella patens]	--
LNC_004572	-1.595195795	Novel_010089	3.697522611	F3'H	flavonoid 3'-hydroxylase [Pohlia nutans]	ull=Flavonoid 3'-monooxygenase; EC=1.14.13.21; AltName: Full=Cytochrome P450 75B2; AltName: Ful
LNC_004572	-1.595195795	rna-Poh0248210.1	3.329889435	F3'H	flavonoid 3'-hydroxylase [Pohlia nutans]	ull=Flavonoid 3'-monooxygenase; EC=1.14.13.21; AltName: Full=Cytochrome P450 75B2; AltName: Ful
LNC_004589	3.584750052	rna-Poh0246110.1	#N/A	CHS	hypothetical protein PHYPA_014791 [Physcomitrella patens]	RLO RecName: Full=Phenylpropanoylacetyl-CoA synthase; EC=2.3.1.218; AltName: Full=Diketide CoA
LNC_004861	-1.341152491	Novel_011132	3.734066046	CHS	hypothetical protein PHYPA_029225 [Physcomitrella patens]	ISY_PINST RecName: Full=Chalcone synthase; EC=2.3.1.74; AltName: Full=Naringenin-chalcone syntha
LNC_004861	-1.341152491	Novel_011337	3.779916808	CHS	hypothetical protein PHYPA_029225 [Physcomitrella patens]	ISY_PINST RecName: Full=Chalcone synthase; EC=2.3.1.74; AltName: Full=Naringenin-chalcone syntha
LNC_004861	-1.341152491	rna-Poh0291180.1	3.667268632	CHS	hypothetical protein PHYPA_029225 [Physcomitrella patens]	ISY_PINST RecName: Full=Chalcone synthase; EC=2.3.1.74; AltName: Full=Naringenin-chalcone syntha
LNC_004933	-1.392334952	rna-Poh0234360.1	-0.497726432	CHS	chalcone synthase [Physcomitrella patens]	SY_ARAAL RecName: Full=Chalcone synthase; EC=2.3.1.74; AltName: Full=Naringenin-chalcone synth
LNC_004956	5.370366456	Novel_011108	2.719253587	HCT	hypothetical protein PHYPA_003798 [Physcomitrella patens]	ansferase; EC=2.3.1.133; AltName: Full=Hydroxycinnamoyl transferase; AltName: Full=Hydroxycinnamo
LNC_004956	5.370366456	rna-Poh0292400.1	3.170027845	HCT	hypothetical protein PHYPA_003798 [Physcomitrella patens]	ansferase; EC=2.3.1.133; AltName: Full=Hydroxycinnamoyl transferase; AltName: Full=Hydroxycinnamo
LNC_004956	5.370366456	Novel_011109	8.400686771	HCT	hypothetical protein PHYPA_003798 [Physcomitrella patens]	ansferase; EC=2.3.1.133; AltName: Full=Hydroxycinnamoyl transferase; AltName: Full=Hydroxycinnamo
LNC_004964	-3.008479234	Novel_011133	4.400687012	CHS	hypothetical protein PHYPA_029225 [Physcomitrella patens]	ISY_PINST RecName: Full=Chalcone synthase; EC=2.3.1.74; AltName: Full=Naringenin-chalcone syntha
LNC_004964	-3.008479234	Novel_011339	3.865268356	CHS	hypothetical protein PHYPA_029225 [Physcomitrella patens]	ISY_PINST RecName: Full=Chalcone synthase; EC=2.3.1.74; AltName: Full=Naringenin-chalcone syntha
LNC_004964	-3.008479234	rna-Poh0291130.1	3.5952411	CHS	hypothetical protein PHYPA_029225 [Physcomitrella patens]	ISY_PINST RecName: Full=Chalcone synthase; EC=2.3.1.74; AltName: Full=Naringenin-chalcone syntha
LNC_005116	6.354010584	rna-Poh0252400.1	#N/A	CcoAOMT	predicted protein [Physcomitrella patens]	ɔyl-CoA O-methyltransferase; EC=2.1.1.104; AltName: Full=Trans-caffeoyl-CoA 3-O-methyltransferase; S

Plant−pathogen interaction

lncRNA ID	og2FoldChange	Cis-Target Gene ID	og2FoldChang	gene name	NR	SWISS
LNC_000201	1.401712302	rna-Poh0359600.1	0.359444613	BAK1	hypothetical protein PHYPA_005406 [Physcomitrella patens]	5-receptor-like serine/threonine-protein kinase SD2-5; EC=2.7.11.1; AltName: Full=S-domain-2 (SD2) rec
LNC_001164	3.703930503	rna-Poh0313840.1	-0.927783405	BAK1	hypothetical protein PHYPA_004602 [Physcomitrella patens]	5-receptor-like serine/threonine-protein kinase SD2-5; EC=2.7.11.1; AltName: Full=S-domain-2 (SD2) rec
LNC_001199	2.702952403	rna-Poh0305260.1	0.852980841	BAK1	leucine-rich repeat receptor-like protein kinase [Pohlia nutans]	TH RecName: Full=Probable leucine-rich repeat receptor-like protein kinase At5g49770; EC=2.7.11.1; Fla
LNC_001307	-1.008970257	rna-Poh0056560.1	-0.496286586	BAK1	leucine-rich repeat receptor-like protein kinase [Pohlia nutans]	I RecName: Full=Probable LRR receptor-like serine/threonine-protein kinase At1g56140; EC=2.7.11.1; F
LNC_002151	3.90806525	rna-Poh0036870.1	-0.359691146	BAK1	hypothetical protein PHYPA_014366 [Physcomitrella patens]	:: Full=Light-sensor Protein kinase; Includes: RecName: Full=Phytochrome; Includes: RecName: Full=Pro
LNC_002369	1.420672037	rna-Poh0298440.1	#N/A	BAK1	hypothetical protein CUMW_039650, partial [Citrus unshiu]	r kinase IV.1; Short=Arabidopsis thaliana lectin-receptor kinase e; Short=AthlecRK-e; Short=LecRK-IV.1;
LNC_002704	1.320330603	rna-Poh0151440.1	-0.227582089	BAK1	hypothetical protein PHYPA_005406 [Physcomitrella patens]	5-receptor-like serine/threonine-protein kinase SD2-5; EC=2.7.11.1; AltName: Full=S-domain-2 (SD2) rec
LNC_002820	1.836328032	rna-Poh0086810.1	#N/A	BAK1	hypothetical protein PHYPA_001034 [Physcomitrella patens]	I RecName: Full=G-type lectin S-receptor-like serine/threonine-protein kinase At1g61500; EC=2.7.11.1; F
LNC_002933	1.104075759	rna-Poh0163010.1	#N/A	BAK1	leucine-rich repeat receptor-like protein kinase [Pohlia nutans]	I RecName: Full=Probable LRR receptor-like serine/threonine-protein kinase At1g56140; EC=2.7.11.1; F
LNC_003525	1.420156901	rna-Poh0010830.1	-0.836392275	BAK1	hypothetical protein PHYPA_029611 [Physcomitrella patens]	5-receptor-like serine/threonine-protein kinase SD2-5; EC=2.7.11.1; AltName: Full=S-domain-2 (SD2) rec
LNC_003828	5.578708272	Novel_008370	0.603181053	BAK1	hypothetical protein PHYPA_009450 [Physcomitrella patens]	ve protein kinase 1 {ECO:0000303|PubMed:28344081}; EC=2.7.11.1 {ECO:0000255|PROSITE-ProRule:
LNC_003828	5.578708272	rna-Poh0370200.1	0.464301718	BAK1	hypothetical protein PHYPA_030920 [Physcomitrella patens]	ve protein kinase 1 {ECO:0000303|PubMed:28344081}; EC=2.7.11.1 {ECO:0000255|PROSITE-ProRule:
LNC_003915	-1.946293471	rna-Poh0171840.1	#N/A	BAK1	predicted protein [Physcomitrella patens]	ARATH RecName: Full=LRR receptor-like serine/threonine-protein kinase FEI 2; EC=2.7.11.1; Flags: Pre
LNC_004136	1.038159241	rna-Poh0328830.1	#N/A	BAK1	hypothetical protein PHYPA_014906 [Physcomitrella patens]	I RecName: Full=Probable LRR receptor-like serine/threonine-protein kinase At1g56130; EC=2.7.11.1; F
LNC_004294	-5.03817676	rna-Poh0354510.1	#N/A	BAK1	hypothetical protein PHYPA_014781 [Physcomitrella patens]	omain containing receptor kinase VII.2; Short=Arabidopsis thaliana lectin-receptor kinase d; Short=Athlec
LNC_000044	1.513385057	rna-Poh0274020.1	-0.409106593	CaMCM	predicted protein [Physcomitrella patens]	: Full=Bcl-2-associated athanogene 6 {ECO:0000303|Ref.3}; AltName: Full=CaM-binding protein 1 {ECC
LNC_000176	1.58945572	Novel_000931	-0.645878091	CaMCM	hypothetical protein PHYPA_016881 [Physcomitrella patens]	3_ORYSJ RecName: Full=Probable calcium-binding protein CML28; AltName: Full=Calmodulin-like prot
LNC_000176	1.58945572	rna-Poh0046620.1	-0.317349972	CaMCM	hypothetical protein PHYPA_016881 [Physcomitrella patens]	3_ORYSJ RecName: Full=Probable calcium-binding protein CML28; AltName: Full=Calmodulin-like prot
LNC_000177	-2.776592765	Novel_000934	2.937628978	CaMCM	hypothetical protein PHYPA_016924 [Physcomitrella patens]	: Full=Bcl-2-associated athanogene 6 {ECO:0000303|Ref.3}; AltName: Full=CaM-binding protein 1 {ECC
LNC_000177	-2.776592765	rna-Poh0046470.1	2.852400193	CaMCM	hypothetical protein PHYPA_016924 [Physcomitrella patens]	: Full=Bcl-2-associated athanogene 6 {ECO:0000303|Ref.3}; AltName: Full=CaM-binding protein 1 {ECC
LNC_000177	-2.776592765	rna-Poh0046580.1	2.999369644	CaMCM	hypothetical protein PHYPA_016881 [Physcomitrella patens]	--
LNC_000265	3.869748872	rna-Poh0287580.1	-1.573563816	CaMCM	hypothetical protein PHYPA_024094 [Physcomitrella patens]	a(+)/Ca(2+)-exchange protein NCL2 {ECO:0000305}; AltName: Full=OsEFCAX2 {ECO:0000303|PubMe
LNC_000549	-2.289577802	Novel_001124	0.043647854	CaMCM	predicted protein [Physcomitrella patens]	CALM_MOUSC RecName: Full=Calmodulin; Short=CaM;
LNC_000549	-2.289577802	Novel_001323	0.055707268	CaMCM	predicted protein [Physcomitrella patens]	CALM_MOUSC RecName: Full=Calmodulin; Short=CaM;
LNC_000549	-2.289577802	rna-Poh0082780.1	-0.11281075	CaMCM	predicted protein [Physcomitrella patens]	CALM_MOUSC RecName: Full=Calmodulin; Short=CaM;
LNC_000549	-2.289577802	rna-Poh0082790.1	-0.128624127	CaMCM	predicted protein [Physcomitrella patens]	CALM_MOUSC RecName: Full=Calmodulin; Short=CaM;
LNC_000658	-2.15773892	Novel_001910	-0.825940814	CaMCM	hypothetical protein PHYPA_003997 [Physcomitrella patens]	a(+)/Ca(2+)-exchange protein NCL1 {ECO:0000305}; AltName: Full=OsEFCAX1 {ECO:0000303|PubMe
LNC_000658	-2.15773892	rna-Poh0346480.1	-0.696744646	CaMCM	hypothetical protein PHYPA_003997 [Physcomitrella patens]	a(+)/Ca(2+)-exchange protein NCL1 {ECO:0000305}; AltName: Full=OsEFCAX1 {ECO:0000303|PubMe
LNC_000700	3.620465214	rna-Poh0352020.1	#N/A	CaMCM	predicted protein [Physcomitrella patens]	3_ORYSJ RecName: Full=Probable calcium-binding protein CML13; AltName: Full=Calmodulin-like prot
LNC_000710	-4.238926523	rna-Poh0118680.1	-1.007301835	CaMCM	centrin, partial [Acetabularia acetabulum]	3_ORYSJ RecName: Full=Probable calcium-binding protein CML13; AltName: Full=Calmodulin-like prot
LNC_000823	4.191335793	Novel_001997	0.813391258	CaMCM	hypothetical protein PHYPA_012656 [Physcomitrella patens]	_ARATH RecName: Full=Probable calcium-binding protein CML49; AltName: Full=Calmodulin-like pro
LNC_000823	4.191335793	rna-Poh0145800.1	0.610590156	CaMCM	hypothetical protein PHYPA_012656 [Physcomitrella patens]	_ARATH RecName: Full=Probable calcium-binding protein CML49; AltName: Full=Calmodulin-like pro
LNC_001014	1.874567225	rna-Poh0225310.1	-0.650030216	CaMCM	predicted protein [Physcomitrella patens]	_ARATH RecName: Full=Probable calcium-binding protein CML21; AltName: Full=Calmodulin-like pro
LNC_001104	-2.118496989	Novel_002474	4.551128339	CaMCM	predicted protein [Physcomitrella patens]	CML2_ORYSJ RecName: Full=Putative calmodulin-like protein 2; Flags: Precursor;
LNC_001104	-2.118496989	rna-Poh0226150.1	4.291800247	CaMCM	predicted protein [Physcomitrella patens]	CML2_ORYSJ RecName: Full=Putative calmodulin-like protein 2; Flags: Precursor;
LNC_001137	1.046957103	rna-Poh0223240.1	0.788363325	CaMCM	hypothetical protein PHYPA_007512 [Physcomitrella patens]	_ARATH RecName: Full=Probable calcium-binding protein CML25; AltName: Full=Calmodulin-like pro
LNC_001141	3.109453463	Novel_002512	1.603651834	CaMCM	hypothetical protein PHYPA_020328 [Physcomitrella patens]	_ARATH RecName: Full=Probable calcium-binding protein CML49; AltName: Full=Calmodulin-like pro
LNC_001141	3.109453463	rna-Poh0222880.1	1.604539443	CaMCM	hypothetical protein PHYPA_020328 [Physcomitrella patens]	_ARATH RecName: Full=Probable calcium-binding protein CML49; AltName: Full=Calmodulin-like pro
LNC_001228	1.40794281	rna-Poh0058120.1	-0.609002833	CaMCM	hypothetical protein PHYPA_007512 [Physcomitrella patens]	_ARATH RecName: Full=Probable calcium-binding protein CML25; AltName: Full=Calmodulin-like pro
LNC_001286	-1.654414425	rna-Poh0067350.1	#N/A	CaMCM	predicted protein [Physcomitrella patens]	_ARATH RecName: Full=Probable calcium-binding protein CML25; AltName: Full=Calmodulin-like pro
LNC_001286	-1.654414425	rna-Poh0067360.1	-0.627202374	CaMCM	predicted protein [Physcomitrella patens]	_ARATH RecName: Full=Probable calcium-binding protein CML25; AltName: Full=Calmodulin-like pro
LNC_001302	4.541552788	Novel_003087	4.050063478	CaMCM	predicted protein [Physcomitrella patens]	--
LNC_001302	4.541552788	rna-Poh0055850.1	3.896057245	CaMCM	predicted protein [Physcomitrella patens]	--
LNC_001796	1.011596628	Novel_003981	-1.218274635	CaMCM	predicted protein [Physcomitrella patens]	CALM_MOUSC RecName: Full=Calmodulin; Short=CaM;
LNC_001796	1.011596628	rna-Poh0108040.1	-1.284652122	CaMCM	predicted protein [Physcomitrella patens]	CALM_MOUSC RecName: Full=Calmodulin; Short=CaM;
LNC_001956	1.789330221	rna-Poh0199990.1	#N/A	CaMCM	hypothetical protein DCAR_009097 [Daucus carota subsp. sativus]	CALM1_SOLTU RecName: Full=Calmodulin-1; Short=CaM-1;
LNC_002774	1.734801416	rna-Poh0093020.1	-1.762844409	CaMCM	hypothetical protein PHYPA_024094 [Physcomitrella patens]	a(+)/Ca(2+)-exchange protein NCL2 {ECO:0000305}; AltName: Full=OsEFCAX2 {ECO:0000303|PubMe
LNC_002793	-1.192155078	rna-Poh0090380.1	0.016662826	CaMCM	hypothetical protein PHYPA_027037 [Physcomitrella patens]	Y5486_ARATH RecName: Full=Uncharacterized protein At5g64816; Flags: Precursor;
LNC_003368	-2.837339675	Novel_007278	-0.000016	CaMCM	hypothetical protein PHYPA_009392 [Physcomitrella patens]	_ARATH RecName: Full=Probable calcium-binding protein CML27; AltName: Full=Calmodulin-like pro
LNC_003368	-2.837339675	Novel_007686	0.27643012	CaMCM	hypothetical protein PHYPA_009392 [Physcomitrella patens]	_ARATH RecName: Full=Probable calcium-binding protein CML27; AltName: Full=Calmodulin-like pro
LNC_003437	1.610205111	Novel_007919	1.805646768	CaMCM	hypothetical protein PHYPA_009390 [Physcomitrella patens]	_ARATH RecName: Full=Probable calcium-binding protein CML25; AltName: Full=Calmodulin-like pro
LNC_003437	1.610205111	rna-Poh0024250.1	2.05726106	CaMCM	hypothetical protein PHYPA_009390 [Physcomitrella patens]	_ARATH RecName: Full=Probable calcium-binding protein CML25; AltName: Full=Calmodulin-like pro
LNC_003677	1.904324223	Novel_008012	4.68780623	CaMCM	predicted protein [Physcomitrella patens]	_ARATH RecName: Full=Probable calcium-binding protein CML25; AltName: Full=Calmodulin-like pro
LNC_003677	1.904324223	rna-Poh0015400.1	2.424711933	CaMCM	predicted protein [Physcomitrella patens]	_ARATH RecName: Full=Probable calcium-binding protein CML25; AltName: Full=Calmodulin-like pro
LNC_003677	1.904324223	rna-Poh0015410.1	3.17549635	CaMCM	predicted protein [Physcomitrella patens]	_ARATH RecName: Full=Probable calcium-binding protein CML25; AltName: Full=Calmodulin-like pro
LNC_004059	1.132500303	rna-Poh0166140.1	-0.379472398	CaMCM	hypothetical protein PHYPA_023848 [Physcomitrella patens]	: Full=Bcl-2-associated athanogene 6 {ECO:0000303|Ref.3}; AltName: Full=CaM-binding protein 1 {ECC
LNC_004118	6.538173324	rna-Poh0172720.1	#N/A	CaMCM	hypothetical protein GPECTOR_13g762 [Gonium pectorale]	: Full=Bcl-2-associated athanogene 6 {ECO:0000303|Ref.3}; AltName: Full=CaM-binding protein 1 {ECC
LNC_004219	-3.717677605	rna-Poh0269750.1	0.66178496	CaMCM	hypothetical protein PHYPA_003661 [Physcomitrella patens]	--
LNC_004254	1.059242287	rna-Poh0134270.1	-0.008091142	CaMCM	hypothetical protein PHYPA_023848 [Physcomitrella patens]	: Full=Bcl-2-associated athanogene 6 {ECO:0000303|Ref.3}; AltName: Full=CaM-binding protein 1 {ECC
LNC_005004	-2.184926163	Novel_011590	3.623811724	CaMCM	hypothetical protein PHYPA_013169 [Physcomitrella patens]	_ARATH RecName: Full=Probable calcium-binding protein CML25; AltName: Full=Calmodulin-like pro
LNC_005004	-2.184926163	rna-Poh0256440.1	4.633509243	CaMCM	predicted protein [Physcomitrella patens]	_ARATH RecName: Full=Probable calcium-binding protein CML25; AltName: Full=Calmodulin-like pro
LNC_005004	-2.184926163	rna-Poh0256450.1	2.925542935	CaMCM	predicted protein [Physcomitrella patens]	_ARATH RecName: Full=Probable calcium-binding protein CML25; AltName: Full=Calmodulin-like pro
LNC_000290	2.470810348	rna-Poh0334460.1	-1.630193754	CDPK	predicted protein [Physcomitrella patens]	ʀecName: Full=Calcium and calcium/calmodulin-dependent serine/threonine-protein kinase; Short=LjCCaI
LNC_000694	4.743681522	Novel_001394	0.54253512	CDPK	calcium-dependent protein kinase [Physcomitrella patens]	nt protein kinase 2 {ECO:0000305}; Short=OsCDPK2 {ECO:0000305}; Short=OsCPK2 {ECO:0000303|F
LNC_000694	4.743681522	rna-Poh0314850.1	0.37100659	CDPK	calcium-dependent protein kinase [Physcomitrella patens]	nt protein kinase 2 {ECO:0000305}; Short=OsCDPK2 {ECO:0000305}; Short=OsCPK2 {ECO:0000303|F
LNC_000694	4.743681522	rna-Poh0314870.1	#N/A	CDPK	calcium-dependent protein kinase [Physcomitrella patens]	CDPKH_ARATH RecName: Full=Calcium-dependent protein kinase 17; EC=2.7.11.1;
LNC_000914	-5.843342609	Novel_002239	0.127278858	CDPK	calcium-dependent protein kinase [Physcomitrella patens]	CDPKH_ARATH RecName: Full=Calcium-dependent protein kinase 17; EC=2.7.11.1;
LNC_000914	-5.843342609	rna-Poh0142760.1	-0.078754387	CDPK	calcium-dependent protein kinase [Physcomitrella patens]	CDPKH_ARATH RecName: Full=Calcium-dependent protein kinase 17; EC=2.7.11.1;
LNC_000914	-5.843342609	rna-Poh0142800.1	#N/A	CDPK	calcium-dependent protein kinase [Physcomitrella patens]	CDPKH_ARATH RecName: Full=Calcium-dependent protein kinase 17; EC=2.7.11.1;
LNC_001492	2.836838588	rna-Poh0309690.1	-0.951021086	CDPK	predicted protein [Physcomitrella patens]	Y1322_ARATH RecName: Full=C2 domain-containing protein At1g63220;
LNC_002399	9.045994255	Novel_005257	-0.474463486	CDPK	hypothetical protein PHYPA_024688 [Physcomitrella patens]	--
LNC_002399	9.045994255	rna-Poh0318670.1	0.44613624	CDPK	hypothetical protein PHYPA_024688 [Physcomitrella patens]	PK13 {ECO:0000305}; Short=OsCPK13 {ECO:0000303|PubMed:15695435}; EC=2.7.11.1 {ECO:00003(
LNC_002399	9.045994255	rna-Poh0318680.1	-0.251526727	CDPK	hypothetical protein PHYPA_024688 [Physcomitrella patens]	PK13 {ECO:0000305}; Short=OsCPK13 {ECO:0000303|PubMed:15695435}; EC=2.7.11.1 {ECO:00003(
LNC_002802	4.681827516	Novel_006502	#N/A	CDPK	predicted protein [Physcomitrella patens]	ʀecName: Full=Calcium and calcium/calmodulin-dependent serine/threonine-protein kinase; Short=LlCCaM
LNC_002802	4.681827516	rna-Poh0089220.1	-1.210253918	CDPK	predicted protein [Physcomitrella patens]	ʀecName: Full=Calcium and calcium/calmodulin-dependent serine/threonine-protein kinase; Short=OsCCa
LNC_003039	-1.013201682	rna-Poh0366010.1	0.09262107	CDPK	hypothetical protein PHYPA_024688 [Physcomitrella patens]	PK13 {ECO:0000305}; Short=OsCPK13 {ECO:0000303|PubMed:15695435}; EC=2.7.11.1 {ECO:00003(
LNC_003039	-1.013201682	rna-Poh0366020.1	0.342580548	CDPK	hypothetical protein PHYPA_024688 [Physcomitrella patens]	PK13 {ECO:0000305}; Short=OsCPK13 {ECO:0000303|PubMed:15695435}; EC=2.7.11.1 {ECO:00003(
LNC_003347	4.207812187	Novel_007235	2.835630564	CDPK	cpk7 calcium-dependent protein kinase [Physcomitrella patens]	um-dependent protein kinase 1; Short=AtCDPK 1; Short=CDPK 1; EC=2.7.11.1; AltName: Full=Calcium
LNC_003347	4.207812187	rna-Poh0116670.1	2.599473658	CDPK	cpk7 calcium-dependent protein kinase [Physcomitrella patens]	um-dependent protein kinase 1; Short=AtCDPK 1; Short=CDPK 1; EC=2.7.11.1; AltName: Full=Calcium
LNC_003496	3.298743059	Novel_008035	2.181415859	CDPK	cpk7 calcium-dependent protein kinase [Physcomitrella patens]	um-dependent protein kinase 1; Short=AtCDPK 1; Short=CDPK 1; EC=2.7.11.1; AltName: Full=Calcium
LNC_003496	3.298743059	Novel_008036	1.756922538	CDPK	cpk7 calcium-dependent protein kinase [Physcomitrella patens]	um-dependent protein kinase 1; Short=AtCDPK 1; Short=CDPK 1; EC=2.7.11.1; AltName: Full=Calcium

Supplementary Table 5. Significantly changed metabolites in *Pohlia nutans* under UV-B Radiation

Index	Formula	Compounds	Class I	Class II
mws0024	C7H6O5	Gallic acid	Flavonoids	Flavanols
mad2085	C22H18O11	Caffeoyl-p-coumaroyl tartaric acid	henolic aci	henolic ac
pme0120	C5H11NO2	5-Aminovaleric acid	cids and de	cids and d
pmb0542	C24H23O14+	Cyanidin 3-O-(6''-Malonylglucoside)	Flavonoids	nthocyani
Hmyp002315	C15H10O7	,7,4'--Tetrahydroxy-Coumaronochrom	Others	Others
pme0434	C30H26O12	Procyanidin B2	Tannins	anthocyan
pme0436	C30H26O12	Procyanidin B3	Tannins	anthocyan
pme0048	C2H7NO2S	2-Aminoethanesulfinic acid	rganic aci	rganic aci
mws0371	C22H32O2	,10,13,16,19-Docosahexaenoic Acid(C	Lipids	ee fatty ac
pme2459	C21H20O11	Luteolin 7-O-glucoside(Cynaroside)	Flavonoids	Flavonoid
pmp000605	C21H20O11	nnone-2-O-β-D-Glucopyranoside from	Quinones	nthraquin
mws0062	C15H10O6	Orobol	Flavonoids	Isoflavone
mws0064	C15H12O6	Eriodictyol	Flavonoids	hydroflav
mws0005	C10H12N2	Tryptamine	Alkaloids	Plumeran
Hmbp002730	C15H10O6	Isoscutellarein	Flavonoids	Flavonoid
pmp000572	C15H10O6	Luteolin	Flavonoids	Flavonoid
Zmhn002422	C16H20O9	Feruloyl glucose	henolic aci	henolic ac
pme0253	C8H15NO3	N-Acetyl-L-leucine	cids and de	cids and d
mws0574	C4H8O3	α-Hydroxyisobutyric acid	rganic aci	rganic aci
mws0206	C4H8O3	(S)-2-Hydroxybutanoicacid	rganic aci	rganic aci
pme2019	C5H10O5	DL-Arabinose	Others	rides and A
pme3279	C15H10O6	2'-Hydroxygenistein	Flavonoids	Isoflavone
pmn001367	C13H16O9	Protocatechuic acid-4-glucoside	henolic aci	henolic ac
mws1401	C7H14N2O3	L-theanine	cids and de	cids and d
pmn001553	C14H20O8	Cimidahurinine	henolic aci	henolic ac
Hmtn001120	C14H20O8	-(2-Hydroxyethyl)-2-O-glucosylohen	henolic aci	henolic ac
pmb2871	C13H16O9	2,5-Dihydroxy benzoic acid O-hexside	henolic aci	henolic ac
mws0914	C15H12O5	Pinobanksin	Flavonoids	hydroflav
pme2049	C4H8O3	2-Hydroxybutanoic acid	rganic aci	rganic aci
pmb2591	C13H14N2O3	Acetyltryptophan	cids and de	cids and d
pme0170	C8H16N4O3	N-α-Acetyl-L-arginine	cids and de	cids and d
mws4176	C12H16N2O3	DL-Alanyl-DL-phenylalanine	cids and de	cids and d
mws0629	C13H16N2O5	Asp-phe	cids and de	cids and d
mws0344	C5H10O6	D-Xylonic acid	rganic aci	rganic aci
mws4134	C20H32N6O12S2	Oxidized Glutathione	cids and de	cids and d
Lmgn002843	C15H10O6	2'-Hydroxyisoflavone	Flavonoids	Isoflavone
pme1383	C8H11NO3	Pyridoxine	Others	Vitamin
pme3475	C15H12O5	Butin	Flavonoids	hydroflav
Hmln002199	C23H22O13	uercetin 3-O-(6″-O-acetyl)-galactosid	Flavonoids	Flavonols
mws1499	C5H10O5	D-(-)-Arabinose	Others	rides and A
pmd0160	C21H44NO7P	LysoPE 16:0(2n isomer)	Lipids	LPE
pme3388	C7H16N4O2	H-HomoArg-OH	cids and de	cids and d
mws0884	C10H12N5O6P	Cyclic AMP	ides and de	ides and d
mws4171	C8H8O5	Methyl 2,4,6-trihydroxybenzoate	henolic aci	henolic ac
mws0341	C6H12O3	(S)-(-)-2-Hydroxyisocaproic acid	rganic aci	rganic aci
mws0467	C9H10O3	3-(4-Hydroxyphenyl)-propionic acid	henolic aci	henolic ac
pme0376	C15H12O5	Naringenin	Flavonoids	hydroflav
mws0520	C11H13NO4	N-Acetyl-L-tyrosine	cids and de	cids and d
mws0736	C8H16N2O3	N-Glycyl-L-leucine	cids and de	cids and d
mws5042	C11H14N2O3	Glycylphenylalanine	cids and de	cids and d
pmn001526	C20H20O14	1,6-Bis-O-galloyl-β-D-glucose	henolic aci	henolic ac
Lmtn002565	C14H18O9	1'-O-Vanilloyl-β-D-glucoside	henolic aci	henolic ac
Hmpp003270	C21H20O11	Luteolin-4'-O-β-D-glucoside	Flavonoids	Flavonoid

Supplementary Table 6. A list of differentially expressed genes in *Pohlia nutans* under UV-B Radiation

ID	log2FoldCh	pvalue	padj	regulated	GeneID	GeneName	NR	Swissprot	Tremble	KOG	GO	TF-Family	TF-Categor	TF-Classific
rna-Poh02	10.36077	0	0	up	Poh02133	Poh02133	ELIP1 [Syntrichia caninervis]	ELI5_HORVU RecName: Full=High molecular mass early light-inducible protein HV58, chloroplastic; Short=ELI	A0A0F7LCR8_9BRYO Sub	--	GO:00319	--	--	--
rna-Poh03	4.348263	0	0	up	Poh03600	Poh03600	hypothetical protein PHYPA_005253 [Physcomitrella patens]	PPH_ARATH RecName: Full=Pheophytinase, chloroplastic; EC=3.1.1.-; AltName: Full=Pheophytin pheophorbi	A9S8D0_PHYPA SubNam	Predicted	GO:00095	--	--	--
rna-Poh01	-4.39307	0	0	down	Poh01371	Poh01371	hypothetical protein PHYPA_010385 [Physcomitrella patens]	CAHC_HORVU RecName: Full=Carbonic anhydrase, chloroplastic; EC=4.2.1.1; AltName: Full=Carbonate dehy	A9TMC2_PHYPA RecNam	Predicted	GO:00095	--	--	--
rna-Poh03	5.046577	0	0	up	Poh03299	Poh03299	predicted protein [Physcomitrella patens]	TIL_ARATH RecName: Full=Temperature-induced lipocalin-1 {ECO:0000303|PubMed:18671872}; Short=AtTIL	Q38JD1_PHYPA SubNam	Apolipopr	GO:00099	--	--	--
rna-Poh02	8.489575	0	0	up	Poh02646	Poh02646	predicted protein [Physcomitrella patens]	--	A9SCT6_PHYPA SubNam	--	GO:00048	--	--	--
rna-Poh00	-4.43218	0	0	down	Poh00434	Poh00434	predicted protein [Physcomitrella patens]	CB2_PHYPA RecName: Full=Chlorophyll a-b binding protein, chloroplastic; AltName: Full=LHCII type I CAB; Sh	A9T914_PHYPA RecNam	--	GO:00099	--	--	--
rna-Poh00	-5.18838	0	0	down	Poh00405	Poh00405	terpene synthase 3 [Pseudotaxiphyllum elegans]	--	A0A1J0CQ86_9BRYO Sub	--	--	--	--	--
rna-Poh01	8.761293	0	0	up	Poh01052	Poh01052	ELIP2 [Syntrichia caninervis]	ELIP1_ARATH RecName: Full=Early light-induced protein 1, chloroplastic {ECO:0000305}; Flags: Precursor;	A0A0F7LA41_9BRYO Sub	--	GO:00095	--	--	--
rna-Poh01	-5.77342	0	0	down	Poh01081	Poh01081	cellulose synthase-like A1, glycosyltransferase family 2 protein; m	CSLA9_ARATH RecName: Full=Glucomannan 4-beta-mannosyltransferase 9; EC=2.4.1.32; AltName: Full=Cellu	Q1ZZG0_PHYPA SubNam	--	GO:00001	--	--	--
rna-Poh02	4.279118	0	0	up	Poh02762	Poh02762	hypothetical protein PHYPA_029056 [Physcomitrella patens]	SAT5_ARATH RecName: Full=Serine acetyltransferase 5; Short=AtSAT-5; EC=2.3.1.30; AltName: Full=AtSERAT	A9U0K5_PHYPA SubNam	Serine O-a	GO:00058	--	--	--
rna-Poh03	6.420781	0	0	up	Poh03323	Poh03323	hypothetical protein PHYPA_024349 [Physcomitrella patens]	--	A9TRR3_PHYPA SubNam	--	--	--	--	--
rna-Poh01	-4.91167	1.37E-306	4.92E-303	down	Poh01547	Poh01547	hypothetical protein PHYPA_005116 [Physcomitrella patens]	DR100_ARATH RecName: Full=DNA damage-repair/toleration protein DRT100; Flags: Precursor;	A9T3S2_PHYPA SubNam	FOG: Leuci	GO:00095	--	--	--
rna-Poh01	-4.44027	5.35E-302	1.77E-298	down	Poh01638	Poh01638	PINB AtPIN5/6-like auxin efflux carrier protein [Physcomitrella pa	PIN3_ARATH RecName: Full=Auxin efflux carrier component 3 {ECO:0000303|PubMed:11845211}; Short=At	A9RN82_PHYPA RecNam	--	GO:00099	--	--	--
rna-Poh00	8.640445	8.52E-293	2.62E-289	up	Poh00840	Poh00840	predicted protein [Physcomitrella patens]	CATA1_ARATH RecName: Full=Catalase-1; EC=1.11.1.6;	A9TG35_PHYPA SubNam	Catalase	GO:00056	--	--	--
rna-Poh01	6.506536	2.03E-290	5.81E-287	up	Poh01746	Poh01746	hypothetical protein PHYPA_002304 [Physcomitrella patens]	PTC52_ARATH RecName: Full=Protochlorophyllide-dependent translocon component 52, chloroplastic; AltN	A9RGB0_PHYPA SubNam	--	GO:00095	--	--	--
rna-Poh00	3.847619	4.70E-287	1.26E-283	up	Poh00526	Poh00526	predicted protein [Physcomitrella patens]	ANXD5_ARATH RecName: Full=Annexin D5; AltName: Full=AnnAt5;	A9RZF7_PHYPA RecNam	Annexin	GO:00510	--	--	--
rna-Poh01	6.073806	3.73E-279	8.90E-276	up	Poh01054	Poh01054	hypothetical protein PHYPA_026811 [Physcomitrella patens]	VEP1_ARATH RecName: Full=3-oxo-Delta(4,5)-steroid 5-beta-reductase; EC=1.3.1.3 {ECO:0000269|PubMed:	A9RJ73_PHYPA SubNam	--	GO:00058	--	--	--
rna-Poh02	5.482094	2.83E-276	6.39E-273	up	Poh02004	Poh02004	predicted protein [Physcomitrella patens]	MYBF_ARATH RecName: Full=Putative Myb family transcription factor At1g14600;	A9SS29_PHYPA SubNam	--	GO:00056	GARP-G2-l	TF	GARP->GAI
rna-Poh02	-6.39499	4.89E-276	1.05E-272	down	Poh02222	Poh02222	hypothetical protein PHYPA_018301 [Physcomitrella patens]	--	A9S9N2_PHYPA SubNam	--	--	--	--	--
rna-Poh00	7.157275	7.17E-276	1.47E-272	up	Poh00137	Poh00137	--	--	--	--	--	--	--	--
rna-Poh00	-4.75637	8.95E-273	1.75E-269	down	Poh00979	Poh00979	hypothetical protein PHYPA_001919 [Physcomitrella patens]	EMS1_ARATH RecName: Full=Leucine-rich repeat receptor protein kinase EMS1 {ECO:0000305}; EC=2.7.11.1	A9SS32_PHYPA SubNam	--	GO:00160	--	--	--
rna-Poh01	9.057809	2.41E-272	4.50E-269	up	Poh01413	Poh01413	predicted protein [Physcomitrella patens]	--	A9TQE1_PHYPA SubNam	--	--	--	--	--
rna-Poh03	-5.25738	3.96E-272	7.10E-269	down	Poh03258	Poh03258	hypothetical protein PHYPA_017118 [Physcomitrella patens]	--	A9S6Q8_PHYPA SubNam	--	--	--	--	--
rna-Poh03	6.995672	4.03E-267	6.93E-264	up	Poh03402	Poh03402	predicted protein [Physcomitrella patens]	--	A9S9U9_PHYPA SubNam	--	GO:00160	--	--	--
rna-Poh02	4.696847	9.25E-265	1.53E-261	up	Poh02040	Poh02040	hypothetical protein PHYPA_013319 [Physcomitrella patens]	Y2104_ARATH RecName: Full=Uncharacterized methyltransferase At2g41040, chloroplastic; EC=2.1.1.-; Flags	A9TJJ2_PHYPA SubName	--	GO:00095	--	--	--
rna-Poh00	-4.40099	1.70E-263	2.70E-260	down	Poh00035	Poh00035	predicted protein [Physcomitrella patens]	LOX3_ARATH RecName: Full=Lipoxygenase 3, chloroplastic; Short=AtLOX3; EC=1.13.11.12; Flags: Precursor;	A9U1H4_PHYPA SubNam	--	GO:00095	--	--	--
rna-Poh01	5.268786	1.09E-261	1.67E-258	up	Poh01778	Poh01778	predicted protein [Physcomitrella patens]	CADH1_ORYSJ RecName: Full=Probable cinnamyl alcohol dehydrogenase 1 {ECO:0000305}; Short=OsCAD1 {E	A9SJC9_PHYPA SubName	Alcohol de	GO:00455	--	--	--
rna-Poh00	7.819602	2.46E-260	3.65E-257	up	Poh00727	Poh00727	predicted protein [Physcomitrella patens]	VITH1_ORYSJ RecName: Full=Vacuolar iron transporter homolog 1; AltName: Full=Protein NODULIN-LIKE 1;	A9T1R5_PHYPA SubNam	Uncharact	GO:00160	--	--	--
rna-Poh00	5.5952	4.66E-260	6.67E-257	up	Poh00007	Poh00007	--	--	--	--	--	--	--	--
Novel_008	8.927108	1.09E-253	1.50E-250	up	XLOC_035	--	predicted protein [Physcomitrella patens]	ADH1_PETHY RecName: Full=Alcohol dehydrogenase 1 {ECO:0000303|PubMed:1678286}; EC=1.1.1.1 {ECO:0	A9U4C5_PHYPA SubNam	--	GO:00057	--	--	--
rna-Poh00	-4.46532	5.23E-252	6.88E-249	down	Poh00174	Poh00174	predicted protein [Physcomitrella patens]	LOX3_ARATH RecName: Full=Lipoxygenase 3, chloroplastic; Short=AtLOX3; EC=1.13.11.12; Flags: Precursor;	A9U1H4_PHYPA SubNam	--	GO:00095	--	--	--
rna-Poh01	-7.32742	5.29E-252	6.88E-249	down	Poh01748	Poh01748	hypothetical protein PHYPA_018657 [Physcomitrella patens]	--	A9RG10_PHYPA SubNam	--	GO:00160	--	--	--
rna-Poh02	6.350133	4.15E-251	5.25E-248	up	Poh02409	Poh02409	predicted protein [Physcomitrella patens]	--	A9TFJ7_PHYPA SubName	--	--	--	--	--
Novel_007	9.163615	5.06E-249	6.21E-246	up	XLOC_034	--	--	--	--	--	--	--	--	--
Novel_001	10.6199	6.33E-249	7.56E-246	up	XLOC_005	--	--	--	--	--	--	--	--	--
rna-Poh03	-5.88376	1.58E-246	1.84E-243	down	Poh03386	Poh03386	cellulose synthase-like A1, glycosyltransferase family 2 protein; m	CSLA9_ARATH RecName: Full=Glucomannan 4-beta-mannosyltransferase 9; EC=2.4.1.32; AltName: Full=Cellu	Q1ZZG0_PHYPA SubNam	--	GO:00001	--	--	--
rna-Poh03	-4.66481	3.24E-245	3.66E-242	down	Poh03677	Poh03677	hypothetical protein PHYPA_013691 [Physcomitrella patens]	--	A0A176VWP8_MARPO S	--	--	--	--	--
rna-Poh01	6.802792	1.56E-244	1.72E-241	up	Poh01600	Poh01600	hypothetical protein PHYPA_027909 [Physcomitrella patens]	MYBF_ARATH RecName: Full=Putative Myb family transcription factor At1g14600;	A9SYH6_PHYPA SubNam	--	GO:00056	GARP-G2-l	TF	GARP->GAI
rna-Poh03	-4.55779	2.09E-244	2.25E-241	down	Poh03628	Poh03628	predicted protein [Physcomitrella patens]	CB2_PHYPA RecName: Full=Chlorophyll a-b binding protein, chloroplastic; AltName: Full=LHCII type I CAB; Sh	A9T914_PHYPA RecNam	--	GO:00099	--	--	--
rna-Poh02	-3.96793	4.92E-240	5.15E-237	down	Poh02722	Poh02722	unknown [Picea sitchensis]	PLA15_ARATH RecName: Full=Phospholipase A1-Igamma1, chloroplastic; EC=3.1.1.-; Flags: Precursor;	B8LKE3_PICSI SubName:	Predicted	GO:00095	--	--	--
rna-Poh03	-6.43188	1.16E-237	1.18E-234	down	Poh03677	Poh03677	cellulose synthase-like A1, glycosyltransferase family 2 protein; m	CSLA9_ARATH RecName: Full=Glucomannan 4-beta-mannosyltransferase 9; EC=2.4.1.32; AltName: Full=Cellu	Q1ZZG0_PHYPA SubNam	--	GO:00001	--	--	--
rna-Poh02	6.197046	3.26E-235	3.26E-232	up	Poh02130	Poh02130	adh_short domain-containing protein [Cephalotus follicularis]	TIC32_PEA RecName: Full=Short-chain dehydrogenase TIC 32, chloroplastic; EC=1.1.1.-; AltName: Full=Transl	A0A1Q3CUE9_CEPFO Sul	Dehydroge	GO:00097	--	--	--
rna-Poh03	4.112611	6.25E-235	6.11E-232	up	Poh03374	Poh03374	hypothetical protein PHYPA_021078 [Physcomitrella patens]	SPS2_ORYSJ RecName: Full=Solanesyl-diphosphate synthase 2, chloroplastic; Short=OsSPS2; EC=2.5.1.84; Alt	A9TB35_PHYPA SubNam	Geranylger	GO:00095	--	--	--
rna-Poh03	4.021183	1.61E-234	1.53E-231	up	Poh03009	Poh03009	hypothetical protein PHYPA_029056 [Physcomitrella patens]	SAT5_ARATH RecName: Full=Serine acetyltransferase 5; Short=AtSAT-5; EC=2.3.1.30; AltName: Full=AtSERAT	A9U0K5_PHYPA SubNam	Serine O-a	GO:00058	--	--	--
rna-Poh01	6.933951	3.20E-234	2.99E-231	up	Poh01022	Poh01022	hypothetical protein PHYPA_018406 [Physcomitrella patens]	SRC2_ARATH RecName: Full=Protein SRC2 homolog {ECO:0000305}; Short=AtSRC2 {ECO:0000303|PubMed:1	A9RZ05_PHYPA SubNam	--	GO:00057	--	--	--
rna-Poh01	-4.76903	8.98E-234	8.21E-231	down	Poh01212	Poh01212	hypothetical protein PHYPA_013843 [Physcomitrella patens]	PLP2_ORYSI RecName: Full=Patatin-like protein 2; EC=3.1.1.-;	A9SZF5_PHYPA RecName	Ca2+-indep	GO:00167	--	--	--
rna-Poh01	4.075527	5.45E-233	4.88E-230	up	Poh01522	Poh01522	hypothetical protein PHYPA_005253 [Physcomitrella patens]	PPH_ARATH RecName: Full=Pheophytinase, chloroplastic; EC=3.1.1.-; AltName: Full=Pheophytin pheophorbi	A9S8D0_PHYPA SubNam	Predicted	GO:00095	--	--	--
rna-Poh03	-6.47226	2.28E-230	2.00E-227	down	Poh03595	Poh03595	predicted protein [Physcomitrella patens]	PME53_ARATH RecName: Full=Probable pectinesterase 53; Short=PE 53; EC=3.1.1.11; AltName: Full=Pectin	A9SSB9_PHYPA RecNam	--	GO:00056	--	--	--
rna-Poh02	3.484215	8.76E-230	7.53E-227	up	Poh02824	Poh02824	predicted protein [Physcomitrella patens]	STEP2_ARATH RecName: Full=Stress enhanced protein 2, chloroplastic {ECO:0000305}; Flags: Precursor;	A9RL12_PHYPA SubNam	--	GO:00095	--	--	--
rna-Poh01	-4.42452	9.20E-226	7.75E-223	down	Poh01137	Poh01137	hypothetical protein PHYPA_008332 [Physcomitrella patens]	XTH_WHEAT RecName: Full=Probable xyloglucan endotransglucosylase/hydrolase; EC=2.4.1.207; Flags: Prec	A9TST9_PHYPA RecNam	--	GO:00480	--	--	--
rna-Poh01	5.871219	8.21E-225	6.79E-222	up	Poh01922	Poh01922	predicted protein [Physcomitrella patens]	MPCP1_ARATH RecName: Full=Mitochondrial phosphate carrier protein 1, mitochondrial; AltName: Full=Mit	A9SXT6_PHYPA SubNam	Mitochond	GO:00160	--	--	--
rna-Poh02	8.572098	1.40E-224	1.13E-221	up	Poh02625	Poh02625	--	--	--	--	--	--	--	--
rna-Poh03	-5.27569	1.19E-223	9.49E-221	down	Poh03404	Poh03404	--	--	--	--	--	--	--	--
rna-Poh03	6.067385	1.20E-222	9.38E-220	up	Poh03310	Poh03310	hypothetical protein PHYPA_023360 [Physcomitrella patens]	PDX2_ARATH RecName: Full=Probable pyridoxal 5'-phosphate synthase subunit PDX2; Short=AtPDX2; EC=4.3	A9RWY1_PHYPA SubNan	Imidazoleg	GO:00058	--	--	--
rna-Poh00	4.626784	2.35E-221	1.80E-218	up	Poh00338	Poh00338	hypothetical protein PHYPA_013319 [Physcomitrella patens]	Y2104_ARATH RecName: Full=Uncharacterized methyltransferase At2g41040, chloroplastic; EC=2.1.1.-; Flags	A9TJJ2_PHYPA SubName	--	GO:00095	--	--	--
rna-Poh02	-5.23077	6.08E-221	4.58E-218	down	Poh02526	Poh02526	hypothetical protein PHYPA_014250 [Physcomitrella patens]	--	A9RWF7_PHYPA SubNan	--	GO:00160	--	--	--
rna-Poh03	-5.29183	5.01E-219	3.71E-216	down	Poh03546	Poh03546	hypothetical protein PHYPA_022693 [Physcomitrella patens]	NEP2_NEPGR RecName: Full=Aspartic proteinase nepenthesin-2; EC=3.4.23.12; AltName: Full=Nepenthesin-I	A9RMH8_PHYPA SubNan	Aspartyl pr	GO:00055	--	--	--
Novel_005	-4.23332	3.65E-217	2.66E-214	down	XLOC_025	--	cellulose synthase catalytic subunit [Physcomitrella patens]	CESA5_ORYSI RecName: Full=Probable cellulose synthase A catalytic subunit 5 [UDP-forming]; EC=2.4.1.12; A	Q3Y4F6_PHYPA RecNam	--	GO:00160	--	--	--
rna-Poh03	-5.27943	3.89E-217	2.79E-214	down	Poh03258	Poh03258	hypothetical protein PHYPA_017118 [Physcomitrella patens]	--	A9S6Q8_PHYPA SubNam	--	--	--	--	--
rna-Poh00	-6.89998	3.06E-216	2.16E-213	down	Poh00359	Poh00359	hypothetical protein PHYPA_018657 [Physcomitrella patens]	--	A9RG10_PHYPA SubNam	--	GO:00160	--	--	--
rna-Poh00	-3.84575	3.01E-214	2.09E-211	down	Poh00631	Poh00631	hypothetical protein PHYPA_008310 [Physcomitrella patens]	ERG1_ORYSI RecName: Full=Elicitor-responsive protein 1; AltName: Full=17 kDa phloem protein; AltName: F	A0A0K9PV75_ZOSMR Sul	Predicted	GO:00057	--	--	--
Novel_000	5.632748	5.02E-213	3.43E-210	up	XLOC_002	--	hypothetical protein CFP56_69038 [Quercus suber]	--	--	--	--	--	--	--
rna-Poh02	-4.09715	5.79E-213	3.89E-210	down	Poh02646	Poh02646	hypothetical protein PHYPA_025198 [Physcomitrella patens]	COBL3_ORYSJ RecName: Full=COBRA-like protein 3; AltName: Full=Protein BRITTLE CULM1-like 4; Flags: Prec	D8SX60_SELML RecName	--	GO:00312	--	--	--
rna-Poh00	-3.18495	4.81E-212	3.18E-209	down	Poh00631	Poh00631	predicted protein [Physcomitrella patens]	TBA1_ORYSJ RecName: Full=Tubulin alpha-1 chain;	A9TEQ9_PHYPA RecNam	Alpha tubu	GO:00057	--	--	--
rna-Poh01	3.993516	5.27E-210	3.43E-207	up	Poh01328	Poh01328	hypothetical protein PHYPA_024349 [Physcomitrella patens]	--	A9U4K1_PHYPA SubNam	--	--	--	--	--
rna-Poh03	5.379343	3.78E-209	2.43E-206	up	Poh03684	Poh03684	hypothetical protein PHYPA_001889 [Physcomitrella patens]	PAO_ARATH RecName: Full=Pheophorbide a oxygenase, chloroplastic; Short=AtPaO; Short=Pheide a oxygen	A9SRZ4_PHYPA SubNam	--	GO:00095	--	--	--
rna-Poh00	3.985994	2.00E-208	1.26E-205	up	Poh00536	Poh00536	ferritin, chloroplast precursor [Physcomitrella patens]	FRI3_SOYBN RecName: Full=Ferritin-3, chloroplastic; EC=1.16.3.1; AltName: Full=SFerH-3; Flags: Precursor;	A9S6B6_PHYPA RecNam	Ferritin	GO:00095	--	--	--
rna-Poh03	-6.91672	2.91E-206	1.82E-203	down	Poh03312	Poh03312	predicted protein [Physcomitrella patens]	EXPA1_ARATH RecName: Full=Expansin-A1; Short=AtEXPA1; AltName: Full=Alpha-expansin-1; Short=At-EXP1	A9TKK3_PHYPA SubNam	--	GO:00056	--	--	--
rna-Poh00	6.746744	5.66E-206	3.48E-203	up	Poh00643	Poh00643	--	--	--	--	--	--	--	--
rna-Poh02	4.275122	6.20E-205	3.76E-202	up	Poh02867	Poh02867	photolyase-like protein [Physcomitrella patens]	CRYD_SOLLC RecName: Full=Cryptochrome DASH, chloroplastic/mitochondrial {ECO:0000303|PubMed:1687	A9U403_PHYPA SubNam	Deoxyribo	GO:00095	--	--	--
rna-Poh03	-5.62531	1.91E-204	1.14E-201	down	Poh03515	Poh03515	leucine-rich repeat receptor-like protein kinase [Pohlia nutans]	SRF8_ARATH RecName: Full=Protein STRUBBELIG-RECEPTOR FAMILY 8; AltName: Full=Leucine-rich repeat re	A0A1P8DYY8_9BRYO Sut	Serine/thr	GO:00160	--	--	--
rna-Poh03	-4.58408	5.49E-204	3.24E-201	down	Poh03251	Poh03251	hypothetical protein PHYPA_030633 [Physcomitrella patens]	IQD1_ARATH RecName: Full=Protein IQ-DOMAIN 1 {ECO:0000303|PubMed:15960618};	A9RF75_PHYPA SubNam	--	GO:00057	--	--	--
Novel_009	6.779293	4.70E-202	2.73E-199	up	XLOC_041	--	hypothetical protein PHYPA_024349 [Physcomitrella patens]	--	A9TRR3_PHYPA SubNam	--	--	--	--	--
rna-Poh01	-4.51	7.44E-202	4.26E-199	down	Poh01604	Poh01604	hypothetical protein PHYPA_028897 [Physcomitrella patens]	--	A9TI87_PHYPA SubName	--	--	--	--	--
Novel_007	6.189653	5.19E-201	2.93E-198	up	XLOC_032	--	--	--	--	--	--	--	--	--
rna-Poh03	3.425531	1.52E-200	8.47E-198	up	Poh03422	Poh03422	predicted protein [Physcomitrella patens]	HBPL1_ARATH RecName: Full=Heme-binding-like protein At3g10130, chloroplastic; Flags: Precursor;	A9RNA0_PHYPA SubNam	--	GO:00095	--	--	--
rna-Poh02	-4.07164	1.74E-198	9.57E-196	down	Poh02988	Poh02988	predicted protein [Physcomitrella patens]	--	A9TI87_PHYPA SubName	--	--	--	--	--
rna-Poh01	5.033166	3.58E-198	1.95E-195	up	Poh01276	Poh01276	hypothetical protein PHYPA_017287 [Physcomitrella patens]	FAD4_ARATH RecName: Full=Fatty acid desaturase 4, chloroplastic {ECO:0000303|PubMed:19682287}; EC=1	A9REU2_PHYPA SubNam	Ubiquitin-c	GO:00095	--	--	--
rna-Poh00	4.622513	7.31E-198	3.93E-195	up	Poh00724	Poh00724	hypothetical protein PHYPA_024891 [Physcomitrella patens]	Y1457_ARATH RecName: Full=Acyltransferase-like protein At1g54570, chloroplastic; EC=2.3.1.-; Flags: Precur	A9SUX8_PHYPA SubNam	--	GO:00095	--	--	--
rna-Poh02	4.152737	3.43E-197	1.82E-194	up	Poh02910	Poh02910	predicted protein [Physcomitrella patens]	Y5224_ARATH RecName: Full=Uncharacterized protein At5g02240;	A9TK47_PHYPA SubNam	Predicted	GO:00480	--	--	--
rna-Poh00	-6.11696	3.40E-196	1.78E-193	down	Poh00519	Poh00519	hypothetical protein PHYPA_017625 [Physcomitrella patens]	EXPA2_ORYSJ RecName: Full=Expansin-A2; AltName: Full=Alpha-expansin-2; AltName: Full=OsEXP2; AltName	A9TG12_PHYPA SubNam	--	GO:00056	--	--	--
rna-Poh00	-5.40388	1.72E-195	8.89E-193	down	Poh00518	Poh00518	predicted protein [Physcomitrella patens]	--	A9TF18_PHYPA SubNam	--	GO:00160	--	--	--
rna-Poh00	-5.49081	1.98E-195	1.01E-192	down	Poh00252	Poh00252	predicted protein [Physcomitrella patens]	CER3_ARATH RecName: Full=Protein ECERIFERUM 3; AltName: Full=Protein FACELESS POLLEN 1; AltName: F	A9RQD0_PHYPA SubNam	--	GO:00057	--	--	--
Novel_001	-4.78589	2.48E-195	1.25E-192	down	XLOC_007	--	hypothetical protein PHYPA_017047 [Physcomitrella patens]	IQD1_ARATH RecName: Full=Protein IQ-DOMAIN 1 {ECO:0000303|PubMed:15960618};	A9RF75_PHYPA SubNam	--	GO:00057	--	--	--
rna-Poh03	4.357184	5.22E-195	2.61E-192	up	Poh03016	Poh03016	ATP-binding cassette transporter, subfamily I, member 9, ABC co	AB20I_ARATH RecName: Full=ABC transporter I family member 20; Short=ABC transporter ABCI.20; Short=At	A9T4M7_PHYPA SubNam	Predicted	GO:00057	--	--	--
rna-Poh01	3.581447	6.67E-195	3.30E-192	up	Poh01522	Poh01522	hypothetical protein PHYPA_016601 [Physcomitrella patens]	HS905_ARATH RecName: Full=Heat shock protein 90-5, chloroplastic {ECO:0000305}; Short=AtHSP90.5 {ECO	A9TMP2_PHYPA SubNam	Molecular	GO:00095	--	--	--
rna-Poh02	-5.21802	2.69E-194	1.32E-191	down	Poh02117	Poh02117	predicted protein [Physcomitrella patens]	CER3_ARATH RecName: Full=Protein ECERIFERUM 3; AltName: Full=Protein FACELESS POLLEN 1; AltName: F	A9TT58_PHYPA SubNam	--	GO:00057	--	--	--
rna-Poh01	-6.0358	6.98E-193	3.37E-190	down	Poh01559	Poh01559	predicted protein [Physcomitrella patens]	PME53_ARATH RecName: Full=Probable pectinesterase 53; Short=PE 53; EC=3.1.1.11; AltName: Full=Pectin	A9SSB9_PHYPA RecNam	--	GO:00056	--	--	--
Novel_003	-5.52244	1.34E-192	6.39E-190	down	XLOC_015	--	--	--	--	--	--	--	--	--
rna-Poh03	-4.38027	2.40E-190	1.13E-187	down	Poh03605	Poh03605	hypothetical protein PHYPA_005648 [Physcomitrella patens]	TBA_PRUDU RecName: Full=Tubulin alpha chain;	Q8H932_PHYPA RecNam	Alpha tubu	GO:00057	--	--	--
rna-Poh00	-3.15903	5.18E-189	2.42E-186	down	Poh00630	Poh00630	predicted protein [Physcomitrella patens]	TBA1_ORYSJ RecName: Full=Tubulin alpha-1 chain;	A9TEQ9_PHYPA RecNam	Alpha tubu	GO:00057	--	--	--
rna-Poh00	5.423786	1.35E-188	6.24E-186	up	Poh00136	Poh00136	--	--	--	--	--	--	--	--
rna-Poh00	-4.56309	6.96E-188	3.18E-185	down	Poh00150	Poh00150	predicted protein [Physcomitrella patens]	HIP28_ARATH RecName: Full=Heavy metal-associated isoprenylated plant protein 28 {ECO:0000303|PubMe	A9S8U6_PHYPA SubNam	Copper ch	GO:00057	--	--	--
rna-Poh03	-4.70264	9.56E-188	4.33E-185	down	Poh03062	Poh03062	hypothetical protein PHYPA_020746 [Physcomitrella patens]	TBL25_ARATH RecName: Full=Protein trichome birefringence-like 25;	A9TUK8_PHYPA SubNam	--	GO:00057	--	--	--
rna-Poh02	5.508468	2.31E-186	1.04E-183	up	Poh02356	Poh02356	predicted protein [Physcomitrella patens]	--	A9TFJ7_PHYPA SubName	--	--	--	--	--
rna-Poh03	-5.59849	9.80E-185	4.34E-182	down	Poh03634	Poh03634	hypothetical protein PHYPA_011004 [Physcomitrella patens]	GDL72_ARATH RecName: Full=GDSL esterase/lipase At5g03810; EC=3.1.1.-; AltName: Full=Extracellular lipas	A9RXY9_PHYPA SubNam	--	GO:00055	--	--	--
rna-Poh01	6.649285	1.65E-184	7.22E-182	up	Poh01499	Poh01499	predicted protein [Physcomitrella patens]	AKR2_ORYSJ RecName: Full=Probable aldo-keto reductase 2; EC=1.1.1.-;	A9TGX3_PHYPA SubNam	Voltage-ga	GO:00164	--	--	--
rna-Poh00	-4.18924	6.25E-184	2.71E-181	down	Poh00491	Poh00491	predicted protein [Physcomitrella patens]	CB2_PHYPA RecName: Full=Chlorophyll a-b binding protein, chloroplastic; AltName: Full=LHCII type I CAB; Sh	A9SGM1_PHYPA RecNan	--	GO:00099	--	--	--
rna-Poh03	4.564043	1.47E-182	6.33E-180	up	Poh03081	Poh03081	hypothetical protein PHYPA_001889 [Physcomitrella patens]	PAO_ARATH RecName: Full=Pheophorbide a oxygenase, chloroplastic; Short=AtPaO; Short=Pheide a oxygen	A9SRZ4_PHYPA SubNam	--	GO:00095	--	--	--
rna-Poh02	-4.90468	3.47E-182	1.48E-179	down	Poh02365	Poh02365	hypothetical protein PHYPA_020091 [Physcomitrella patens]	PER5_VITVI RecName: Full=Peroxidase 5 {ECO:0000250|UniProtKB:P22195}; EC=1.11.1.7; Flags: Precursor;	A9SCM2_PHYPA RecNan	--	GO:00055	--	--	--
rna-Poh01	5.240578	4.67E-182	1.97E-179	up	Poh01946	Poh01946	predicted protein [Physcomitrella patens]	SRC2_ARATH RecName: Full=Protein SRC2 homolog {ECO:0000305}; Short=AtSRC2 {ECO:0000303|PubMed:1	A9RZ05_PHYPA SubNam	--	GO:00057	--	--	--
rna-Poh01	3.893723	9.50E-182	3.97E-179	up	Poh01944	Poh01944	hypothetical protein PHYPA_002596 [Physcomitrella patens]	AOX1A_ARATH RecName: Full=Ubiquinol oxidase 1a, mitochondrial; EC=1.10.3.11 {ECO:0000269|PubMed:9	A9T8C5_PHYPA RecNam	--	GO:00160	--	--	--
rna-Poh00	-6.07487	3.82E-180	1.58E-177	down	Poh00846	Poh00846	predicted protein [Physcomitrella patens]	EXPA2_ORYSJ RecName: Full=Expansin-A2; AltName: Full=Alpha-expansin-2; AltName: Full=OsEXP2; AltName	A9TG12_PHYPA SubNam	--	GO:00056	--	--	--
rna-Poh01	3.450176	2.91E-178	1.19E-175	up	Poh01022	Poh01022	hypothetical protein PHYPA_002596 [Physcomitrella patens]	AOX1A_ARATH RecName: Full=Ubiquinol oxidase 1a, mitochondrial; EC=1.10.3.11 {ECO:0000269|PubMed:9	A9T8C5_PHYPA RecNam	--	GO:00160	--	--	--
rna-Poh03	-5.96785	1.19E-177	4.84E-175	down	Poh03127	Poh03127	hypothetical protein PHYPA_007750 [Physcomitrella patens]	KLCR2_ARATH RecName: Full=Protein KINESIN LIGHT CHAIN-RELATED 2 {ECO:0000303|PubMed:23204523};	A9T5J6_PHYPA SubName	Kinesin ligh	GO:00058	--	--	--
rna-Poh01	-4.81367	1.26E-177	5.04E-175	down	Poh01335	Poh01335	predicted protein [Physcomitrella patens]	CATA1_NICPL RecName: Full=Catalase isozyme 1; EC=1.11.1.6; Flags: Fragment;	A9TLM9_PHYPA RecNam	Catalase	GO:00057	--	--	--
rna-Poh00	8.069908	1.39E-177	5.52E-175	up	Poh00109	Poh00109	hypothetical protein PHYPA_021543 [Physcomitrella patens]	HSP13_SOYBN RecName: Full=17.5 kDa class I heat shock protein; AltName: Full=HSP 17.5-M;	A9SGX6_PHYPA SubNam	Molecular	GO:00057	--	--	--
Novel_003	7.138581	2.34E-177	9.22E-175	up	XLOC_015	--	hypothetical protein PHYPA_025707 [Physcomitrella patens]	H41_WHEAT RecName: Full=Histone H4 variant TH011;	A9RT83_PHYPA SubNam	Histone H4	GO:00095	--	--	--
rna-Poh00	-5.05913	6.30E-177	2.46E-174	down	Poh00236	Poh00236	predicted protein [Physcomitrella patens]	--	A9T927_PHYPA SubNam	--	--	--	--	--
rna-Poh01	-3.40941	1.54E-175	5.96E-173	down	Poh01550	Poh01550	hypothetical protein PHYPA_016490 [Physcomitrella patens]	CB12_SOLLC RecName: Full=Chlorophyll a-b binding protein 7, chloroplastic; AltName: Full=LHCI type II CAB-	A9U5E4_PHYPA RecNam	--	GO:00099	--	--	--
rna-Poh02	-3.58125	3.10E-175	1.19E-172	down	Poh02756	Poh02756	hypothetical protein PHYPA_016490 [Physcomitrella patens]	CB12_SOLLC RecName: Full=Chlorophyll a-b binding protein 7, chloroplastic; AltName: Full=LHCI type II CAB-	A9U5E4_PHYPA RecNam	--	GO:00099	--	--	--
rna-Poh00	5.851817	1.22E-174	4.62E-172	up	Poh00770	Poh00770	hypothetical protein AXG93_960s1160 [Marchantia polymorpha	DIR20_ARATH RecName: Full=Dirigent protein 20; Short=AtDIR20; Flags: Precursor;	A0A176VGW5_MARPO R	--	GO:00480	--	--	--
rna-Poh01	4.831075	1.28E-174	4.84E-172	up	Poh01594	Poh01594	predicted protein [Physcomitrella patens]	FBX6_ARATH RecName: Full=F-box only protein 6;	A9SYF4_PHYPA SubName	--	GO:00048	--	--	--
Novel_005	7.953932	2.13E-174	7.97E-172	up	XLOC_023	--	--	--	--	--	--	--	--	--
rna-Poh02	-3.09403	2.40E-172	8.82E-170	down	Poh02192	Poh02192	hypothetical protein PHYPA_004347 [Physcomitrella patens]	CAB6_ARATH RecName: Full=Chlorophyll a-b binding protein 6, chloroplastic; AltName: Full=LHCI-730; AltNa	A9T399_PHYPA RecNam	--	GO:00095	--	--	--
rna-Poh03	-6.94203	2.56E-172	9.34E-170	down	Poh03408	Poh03408	hypothetical protein PHYPA_013257 [Physcomitrella patens]	FBK46_ARATH RecName: Full=F-box/kelch-repeat protein At2g44130;	A9SMQ8_PHYPA SubNan	FOG: Kelch	GO:00058	--	--	--
rna-Poh03	-3.48222	3.27E-172	1.18E-169	down	Poh03300	Poh03300	hypothetical protein PHYPA_024436 [Physcomitrella patens]	ORGRE_CUCME RecName: Full=Protein ORANGE-GREEN, chloroplastic {ECO:0000305}; Short=CmOr-green {E	A9TZ44_PHYPA SubNam	--	GO:00095	--	--	--
rna-Poh02	5.380988	6.47E-172	2.32E-169	up	Poh02040	Poh02040	hypothetical protein PHYPA_001218 [Physcomitrella patens]	DEGP1_ARATH RecName: Full=Protease Do-like 1, chloroplastic; EC=3.4.21.-; AltName: Full=Protein DEGRAD	A9SHE2_PHYPA SubNam	Serine prot	GO:00095	--	--	--
rna-Poh00	4.167798	1.15E-171	4.08E-169	up	Poh00381	Poh00381	hypothetical protein PHYPA_018304 [Physcomitrella patens]	PPDEX_ARATH RecName: Full=DeSI-like protein At4g17486; EC=3.4.-.-;	A9SH91_PHYPA SubNam	Uncharact	GO:00082	--	--	--
rna-Poh00	3.776461	2.23E-171	7.87E-169	up	Poh00201	Poh00201	predicted protein [Physcomitrella patens]	MD15A_ARATH RecName: Full=Mediator of RNA polymerase II transcription subunit 15a;	A9SHI4_PHYPA SubName	--	GO:00165	--	--	--
rna-Poh03	3.604465	3.61E-171	1.26E-168	up	Poh03731	Poh03731	predicted protein [Physcomitrella patens]	--	A9RMC3_PHYPA SubNan	--	--	--	--	--
rna-Poh00	4.127572	4.44E-171	1.54E-168	up	Poh00351	Poh00351	hypothetical protein PHYPA_022287 [Physcomitrella patens]	AB22G_ARATH RecName: Full=ABC transporter G family member 22; Short=ABC transporter ABCG.22; Short	A9SCA8_PHYPA SubNam	Transporte	GO:00160	--	--	--
rna-Poh01	-4.55108	6.45E-171	2.22E-168	down	Poh01454	Poh01454	predicted protein [Physcomitrella patens]	CSPL4_PHYPA RecName: Full=CASP-like protein 1U1; Short=PpCASPL1U1;	A0A176WBF3_MARPO R	--	GO:00160	--	--	--
Novel_009	4.800245	5.70E-170	1.94E-167	up	XLOC_044	--	hypothetical protein PHYPA_025939 [Physcomitrella patens]	--	A9TWY0_PHYPA SubNan	--	--	--	--	--
rna-Poh03	-4.54035	1.03E-169	3.50E-167	down	Poh03511	Poh03511	predicted protein [Physcomitrella patens]	CSPL4_PHYPA RecName: Full=CASP-like protein 1U1; Short=PpCASPL1U1;	A0A176WBF3_MARPO R	--	GO:00160	--	--	--
rna-Poh01	5.134795	1.80E-169	6.06E-167	up	Poh01415	Poh01415	hypothetical protein PHYPA_005820 [Physcomitrella patens]	HTH_ARATH RecName: Full=Protein HOTHEAD; AltName: Full=Protein ADHESION OF CALYX EDGES; Flags: Pre	A0A022RJE8_ERYGU Sub	Glucose de	GO:00055	--	--	--
rna-Poh02	-3.66934	3.34E-169	1.11E-166	down	Poh02161	Poh02161	predicted protein [Physcomitrella patens]	PEAM1_ARATH RecName: Full=Phosphoethanolamine N-methyltransferase 1; Short=AtNMT1; Short=PEAMT	A9SAH0_PHYPA SubNam	SAM-depe	GO:00057	--	--	--
rna-Poh02	-4.50055	4.06E-169	1.34E-166	down	Poh02390	Poh02390	hypothetical protein PHYPA_020145 [Physcomitrella patens]	OFT35_ARATH RecName: Full=O-fucosyltransferase 35 {ECO:0000305}; Short=O-FucT-35 {ECO:0000305}; EC=	A9SX26_PHYPA SubNam	--	GO:00095	--	--	--
rna-Poh00	3.664332	4.81E-169	1.58E-166	up	Poh00952	Poh00952	predicted protein [Physcomitrella patens]	HS22C_SOYBN RecName: Full=Small heat shock protein, chloroplastic; Flags: Fragment;	A9S018_PHYPA SubNam	Molecular	GO:00095	--	--	--
rna-Poh01	-4.5345	6.06E-169	1.97E-166	down	Poh01156	Poh01156	hypothetical protein PHYPA_029897 [Physcomitrella patens]	UCC1_ARATH RecName: Full=Uclacyanin 1 {ECO:0000303|PubMed:9761472}; Flags: Precursor;	A9S925_PHYPA SubNam	--	GO:00312	--	--	--
rna-Poh02	3.580121	1.19E-168	3.84E-166	up	Poh02729	Poh02729	hypothetical protein PHYPA_026935 [Physcomitrella patens]	AB31G_ARATH RecName: Full=ABC transporter G family member 31; Short=ABC transporter ABCG.31; Short	A9RIX0_PHYPA SubName	Pleiotropic	GO:00160	--	--	--
rna-Poh03	-5.81979	1.53E-168	4.90E-166	down	Poh03677	Poh03677	cellulose synthase-like A1, glycosyltransferase family 2 protein; m	CSLA9_ARATH RecName: Full=Glucomannan 4-beta-mannosyltransferase 9; EC=2.4.1.32; AltName: Full=Cellu	Q1ZZG0_PHYPA SubNam	--	GO:00001	--	--	--
rna-Poh01	5.972836	7.99E-168	2.54E-165	up	Poh01446	Poh01446	--	--	--	--	--	--	--	--
rna-Poh00	-3.97168	1.46E-167	4.60E-165	down	Poh00662	Poh00662	hypothetical protein PHYPA_020746 [Physcomitrella patens]	TBL23_ARATH RecName: Full=Protein trichome birefringence-like 23;	A9TUK8_PHYPA SubNam	--	GO:00057	--	--	--
rna-Poh03	3.282532	1.22E-166	3.83E-164	up	Poh03015	Poh03015	hypothetical protein PHYPA_010404 [Physcomitrella patens]	PYRP2_ARATH RecName: Full=5-amino-6-(5-phospho-D-ribitylamino)uracil phosphatase, chloroplastic {ECO:	A9SHZ9_PHYPA SubNam	Predicted	GO:00095	--	--	--
Novel_000	-3.76764	1.80E-166	5.61E-164	down	XLOC_001	--	--	--	--	--	--	--	--	--
rna-Poh00	4.264183	1.01E-165	3.12E-163	up	Poh00208	Poh00208	hypothetical protein PHYPA_021078 [Physcomitrella patens]	SPS2_ORYSJ RecName: Full=Solanesyl-diphosphate synthase 2, chloroplastic; Short=OsSPS2; EC=2.5.1.84; Alt	A9TB35_PHYPA SubNam	Geranylger	GO:00095	--	--	--
Novel_008	7.307377	2.50E-165	7.68E-163	up	XLOC_035	--	--	--	--	--	--	--	--	--
Novel_007	6.880909	9.45E-165	2.88E-162	up	XLOC_031	--	--	--	--	--	--	--	--	--
rna-Poh01	-4.80765	2.03E-164	6.13E-162	down	Poh01620	Poh01620	hypothetical protein PHYPA_028563 [Physcomitrella patens]	TC10B_ARATH RecName: Full=3-dehydrosphinganine reductase TSC10B; EC=1.1.1.102; AltName: Full=3-keto	A9RN63_PHYPA SubNam	Predicted	GO:00057	--	--	--
rna-Poh00	-3.26343	5.33E-164	1.60E-161	down	Poh00898	Poh00898	hypothetical protein PHYPA_016490 [Physcomitrella patens]	CB12_PETHY RecName: Full=Chlorophyll a-b binding protein, chloroplastic; AltName: Full=LHCI type II CAB; F	A9U5E4_PHYPA RecNam	--	GO:00095	--	--	--
Novel_007	8.164159	6.07E-164	1.81E-161	up	XLOC_032	--	--	--	--	--	--	--	--	--
rna-Poh00	-4.47233	4.40E-163	1.30E-160	down	Poh00055	Poh00055	hypothetical protein PHYPA_003432 [Physcomitrella patens]	C70A2_ARATH RecName: Full=Cytochrome P450 703A2 {ECO:0000303|PubMed:17496121}; EC=1.14.13.206	A9RQV1_PHYPA SubNam	Cytochrom	GO:00160	--	--	--
rna-Poh03	3.436755	7.17E-163	2.11E-160	up	Poh03018	Poh03018	hypothetical protein PHYPA_011819 [Physcomitrella patens]	Y1457_ARATH RecName: Full=Acyltransferase-like protein At1g54570, chloroplastic; EC=2.3.1.-; Flags: Precur	A9SUX8_PHYPA SubNam	--	GO:00095	--	--	--
Novel_002	-3.85352	1.08E-162	3.16E-160	down	XLOC_009	--	predicted protein [Physcomitrella patens]	CB2_PHYPA RecName: Full=Chlorophyll a-b binding protein, chloroplastic; AltName: Full=LHCII type I CAB; Sh	A9RNP2_PHYPA RecNam	--	GO:00099	--	--	--
rna-Poh02	-4.31807	1.35E-162	3.91E-160	down	Poh02683	Poh02683	hypothetical protein PHYPA_005511 [Physcomitrella patens]	--	A0A0D6QVW6_ARACU S	Cholestero	GO:00160	--	--	--
rna-Poh00	-4.82952	1.48E-162	4.28E-160	down	Poh00115	Poh00115	hypothetical protein PHYPA_008332 [Physcomitrella patens]	XTH5_ARATH RecName: Full=Probable xyloglucan endotransglucosylase/hydrolase protein 5; Short=At-XTH5	A9TST9_PHYPA RecName	--	GO:00480	--	--	--
Novel_006	-5.91149	1.59E-162	4.54E-160	down	XLOC_027	--	hypothetical protein PHYPA_005173 [Physcomitrella patens]	PME53_ARATH RecName: Full=Probable pectinesterase 53; Short=PE 53; EC=3.1.1.11; AltName: Full=Pectin	A9S853_PHYPA RecName	--	GO:00056	--	--	--
rna-Poh01	-5.07095	3.70E-162	1.05E-159	down	Poh01724	Poh01724	hypothetical protein PHYPA_022693 [Physcomitrella patens]	NEP2_NEPGR RecName: Full=Aspartic proteinase nepenthesin-2; EC=3.4.23.12; AltName: Full=Nepenthesin-I	A9RMH8_PHYPA SubNan	Aspartyl pr	GO:00055	--	--	--
Novel_002	5.516689	7.44E-162	2.09E-159	up	XLOC_010	--	predicted protein [Physcomitrella patens]	--	A9TJS6_PHYPA SubName	--	--	--	--	--
rna-Poh01	3.837811	8.70E-162	2.43E-159	up	Poh01483	Poh01483	hypothetical protein PHYPA_006026 [Physcomitrella patens]	NPC1_ARATH RecName: Full=Non-specific phospholipase C1; EC=3.1.-.-; Flags: Precursor;	A9TGR9_PHYPA SubNam	--	GO:00055	--	--	--
rna-Poh03	3.694381	1.16E-161	3.21E-159	up	Poh03397	Poh03397	hypothetical protein PHYPA_023474 [Physcomitrella patens]	SDEH4_ARATH RecName: Full=Senescence/dehydration-associated protein At4g35985, chloroplastic {ECO:0	A9U3E2_PHYPA SubNam	--	GO:00095	--	--	--
rna-Poh01	8.510098	1.99E-161	5.48E-159	up	Poh01135	Poh01135	hypothetical protein PHYPA_008459 [Physcomitrella patens]	HSP12_MEDSA RecName: Full=18.2 kDa class I heat shock protein;	A9RN10_PHYPA SubNam	Molecular	GO:00057	--	--	--
rna-Poh03	-3.29474	3.61E-161	9.89E-159	down	Poh03197	Poh03197	hypothetical protein PHYPA_024722 [Physcomitrella patens]	CUT1A_ARATH RecName: Full=Protein CURVATURE THYLAKOID 1A, chloroplastic; Flags: Precursor;	A9TE40_PHYPA SubNam	--	GO:00095	--	--	--
rna-Poh01	5.509321	4.16E-161	1.13E-158	up	Poh01047	Poh01047	hypothetical protein B456_009G069700 [Gossypium raimondii]	--	A0A0D2QAK6_GOSRA Su	--	--	--	--	--
rna-Poh01	3.136253	1.78E-160	4.82E-158	up	Poh01719	Poh01719	hypothetical protein PHYPA_026089 [Physcomitrella patens]	PDS_ARATH RecName: Full=15-cis-phytoene desaturase, chloroplastic/chromoplastic; EC=1.3.5.5; AltName: I	A9TY68_PHYPA SubNam	Amine oxi	GO:00095	--	--	--
rna-Poh02	-5.60181	8.65E-160	2.32E-157	down	Poh02746	Poh02746	predicted protein [Physcomitrella patens]	PME53_ARATH RecName: Full=Probable pectinesterase 53; Short=PE 53; EC=3.1.1.11; AltName: Full=Pectin	A9SSB9_PHYPA RecName	--	GO:00056	--	--	--
Novel_009	5.891944	5.12E-159	1.37E-156	up	XLOC_039	--	--	--	--	--	--	--	--	--
rna-Poh01	-4.78249	5.46E-159	1.45E-156	down	Poh01174	Poh01174	hypothetical protein PHYPA_008069 [Physcomitrella patens]	PME53_ARATH RecName: Full=Probable pectinesterase 53; Short=PE 53; EC=3.1.1.11; AltName: Full=Pectin	A9RRD1_PHYPA RecNam	--	GO:00056	--	--	--
rna-Poh03	4.098227	2.67E-158	7.05E-156	up	Poh03517	Poh03517	ATP-binding cassette transporter, subfamily I, member 4, ABC co	AB19I_ARATH RecName: Full=ABC transporter I family member 19; Short=ABC transporter ABCI.19; Short=At	A9RY91_PHYPA SubNam	Predicted	GO:00057	--	--	--
rna-Poh02	4.628972	9.79E-158	2.57E-155	up	Poh02865	Poh02865	PREDICTED: short-chain dehydrogenase TIC 32, chloroplastic-like	TIC32_PEA RecName: Full=Short-chain dehydrogenase TIC 32, chloroplastic; EC=1.1.1.-; AltName: Full=Transl	A0A1J6IPL4_NICAT SubN	Dehydroge	GO:00097	--	--	--
rna-Poh00	2.981015	1.79E-157	4.67E-155	up	Poh00917	Poh00917	photolyase-like protein [Physcomitrella patens]	CRYD_SOLLC RecName: Full=Cryptochrome DASH, chloroplastic/mitochondrial {ECO:0000303|PubMed:1687	A9U403_PHYPA SubNam	Deoxyribo	GO:00095	--	--	--
rna-Poh00	4.926659	1.89E-157	4.90E-155	up	Poh00487	Poh00487	hypothetical protein PHYPA_010951 [Physcomitrella patens]	SAP16_ORYSJ RecName: Full=Zinc finger AN1 and C2H2 domain-containing stress-associated protein 16; Sho	A9TUJ3_PHYPA SubNam	Predicted	GO:00036	--	--	--
rna-Poh03	-5.64529	9.78E-157	2.52E-154	down	Poh03506	Poh03506	predicted protein [Physcomitrella patens]	NDL2_ARATH RecName: Full=Protein NDL2 {ECO:0000305}; AltName: Full=Protein N-MYC DOWNREGULATEL	A9TFP6_PHYPA SubNam	Differentia	GO:00057	--	--	--
rna-Poh00	5.726435	1.35E-156	3.44E-154	up	Poh00248	Poh00248	hypothetical protein PHYPA_001574 [Physcomitrella patens]	--	--	--	--	--	--	--
rna-Poh02	4.48898	2.10E-156	5.35E-154	up	Poh02447	Poh02447	predicted protein [Physcomitrella patens]	Y5224_ARATH RecName: Full=Uncharacterized protein At5g02240;	A9TK47_PHYPA SubNam	Predicted	GO:00480	--	--	--
rna-Poh00	3.745518	7.73E-156	1.95E-153	up	Poh00863	Poh00863	ferritin, chloroplast precursor [Physcomitrella patens]	FRI3_SOYBN RecName: Full=Ferritin-3, chloroplastic; EC=1.16.3.1; AltName: Full=SFerH-3; Flags: Precursor;	A9S6B6_PHYPA RecNam	Ferritin	GO:00095	--	--	--
Novel_001	-4.85662	1.04E-155	2.61E-153	down	XLOC_006	--	hypothetical protein PHYPA_004611 [Physcomitrella patens]	--	--	--	--	--	--	--
Novel_007	-2.79764	1.15E-155	2.87E-153	down	XLOC_035	--	hypothetical protein PHYPA_030798 [Physcomitrella patens]	PSAL_ARATH RecName: Full=Photosystem I reaction center subunit XI, chloroplastic; Short=PSI-L; AltName: F	A9S1E1_PHYPA SubNam	--	GO:00095	--	--	--
Novel_000	-4.06087	5.01E-155	1.25E-152	down	XLOC_003	--	predicted protein [Physcomitrella patens]	CB2_PHYPA RecName: Full=Chlorophyll a-b binding protein, chloroplastic; AltName: Full=LHCII type I CAB; Sh	A9SGM1_PHYPA RecNan	--	GO:00099	--	--	--
rna-Poh01	-3.37734	1.19E-154	2.94E-152	down	Poh01832	Poh01832	predicted protein [Physcomitrella patens]	GLNA_PINSY RecName: Full=Glutamine synthetase cytosolic isozyme; EC=6.3.1.2; AltName: Full=GS1; AltNam	A9RP01_PHYPA RecNam	Glutamine	GO:00057	--	--	--
rna-Poh00	-5.32522	1.68E-154	4.12E-152	down	Poh00786	Poh00786	hypothetical protein PHYPA_014250 [Physcomitrella patens]	--	A9RWF7_PHYPA SubNan	--	GO:00160	--	--	--
rna-Poh03	2.967229	1.18E-153	2.87E-151	up	Poh03065	Poh03065	hypothetical protein PHYPA_029782 [Physcomitrella patens]	TIC32_ARATH RecName: Full=Short-chain dehydrogenase TIC 32, chloroplastic; EC=1.1.1.-; AltName: Full=Tra	A9S5N5_PHYPA SubNam	Dehydroge	GO:00095	--	--	--
rna-Poh01	3.313977	4.23E-153	1.03E-150	up	Poh01304	Poh01304	hypothetical protein PHYPA_011262 [Physcomitrella patens]	--	A9RGM8_PHYPA SubNan	Ubiquinol	GO:00057	--	--	--
rna-Poh01	3.762365	4.49E-153	1.08E-150	up	Poh01521	Poh01521	predicted protein [Physcomitrella patens]	--	A9SUI8_PHYPA SubName	--	GO:00160	--	--	--
rna-Poh03	-2.85814	7.30E-153	1.75E-150	down	Poh03130	Poh03130	predicted protein [Physcomitrella patens]	TBA1_ORYSJ RecName: Full=Tubulin alpha-1 chain;	A9TEQ9_PHYPA RecNam	Alpha tubu	GO:00057	--	--	--
rna-Poh01	-5.30356	1.19E-152	2.84E-150	down	Poh01509	Poh01509	hypothetical protein PHYPA_011004 [Physcomitrella patens]	GDL72_ARATH RecName: Full=GDSL esterase/lipase At5g03810; EC=3.1.1.-; AltName: Full=Extracellular lipas	A9RXY9_PHYPA SubNam	--	GO:00055	--	--	--
rna-Poh01	3.608106	2.01E-152	4.78E-150	up	Poh01051	Poh01051	hypothetical protein PHYPA_002577 [Physcomitrella patens]	HSP7M_PHAVU RecName: Full=Heat shock 70 kDa protein, mitochondrial; Flags: Precursor;	A9T8F3_PHYPA SubName	Molecular	GO:00057	--	--	--
rna-Poh03	6.829266	2.20E-152	5.19E-150	up	Poh03210	Poh03210	predicted protein [Physcomitrella patens]	--	A9S9U9_PHYPA SubNam	--	GO:00160	--	--	--
rna-Poh02	3.71967	3.28E-152	7.71E-150	up	Poh02970	Poh02970	hypothetical protein PHYPA_001686 [Physcomitrella patens]	GSHB_BRAJU RecName: Full=Glutathione synthetase, chloroplastic; Short=GSH synthetase; Short=GSH-S; Sho	A9RTT8_PHYPA RecNam	Glutathion	GO:00095	--	--	--
rna-Poh00	-5.25878	5.14E-152	1.20E-149	down	Poh00993	Poh00993	hypothetical protein PHYPA_002193 [Physcomitrella patens]	--	A9RG10_PHYPA SubNam	--	GO:00160	--	--	--
rna-Poh00	3.284131	5.31E-152	1.23E-149	up	Poh00586	Poh00586	predicted protein [Physcomitrella patens]	SYGM1_ARATH RecName: Full=Glycine--tRNA ligase, mitochondrial 1 {ECO:0000305}; EC=6.1.1.14 {ECO:0000	A9TSY1_PHYPA SubNam	Glycyl-tRN	GO:00058	--	--	--
rna-Poh01	6.949952	6.08E-152	1.40E-149	up	Poh01048	Poh01048	predicted protein [Physcomitrella patens]	MPCP1_ARATH RecName: Full=Mitochondrial phosphate carrier protein 1, mitochondrial; AltName: Full=Mit	A9SXT6_PHYPA SubNam	Mitochond	GO:00160	--	--	--
rna-Poh00	4.288263	7.03E-152	1.62E-149	up	Poh00380	Poh00380	hypothetical protein KFL_000060610 [Klebsormidium nitens]	--	A0A0U9HQL6_KLENI Sub	--	--	--	--	--
rna-Poh00	5.362383	2.58E-151	5.90E-149	up	Poh00165	Poh00165	predicted protein [Physcomitrella patens]	--	A9T5D5_PHYPA SubNam	Zn2+-bindi	GO:00164	--	--	--
rna-Poh00	-6.86113	4.40E-151	1.00E-148	down	Poh00633	Poh00633	hypothetical protein PHYPA_007750 [Physcomitrella patens]	KLCR2_ARATH RecName: Full=Protein KINESIN LIGHT CHAIN-RELATED 2 {ECO:0000303|PubMed:23204523};	A9T5J6_PHYPA SubName	Kinesin ligh	GO:00058	--	--	--
rna-Poh00	-3.25193	8.03E-151	1.82E-148	down	Poh00849	Poh00849	leucine-rich repeat receptor-like protein kinase [Pohlia nutans]	SERK2_ORYSJ RecName: Full=LRR receptor kinase SERK2 {ECO:0000305}; EC=2.7.11.1 {ECO:0000305}; AltNan	A0A1P8DYX8_9BRYO Sub	--	GO:00160	--	--	--
rna-Poh02	3.443878	2.27E-150	5.10E-148	up	Poh02716	Poh02716	hypothetical protein PHYPA_007298 [Physcomitrella patens]	AT18D_ARATH RecName: Full=Autophagy-related protein 18d; Short=AtATG18d;	C0PPX4_PICSI SubName:	Uncharact	GO:00058	--	--	--
rna-Poh02	-4.13471	3.93E-150	8.79E-148	down	Poh02374	Poh02374	predicted protein [Physcomitrella patens]	LOX1_ARATH RecName: Full=Linoleate 9S-lipoxygenase 1; EC=1.13.11.58; AltName: Full=Lipoxygenase 1; Sho	A9U1H4_PHYPA SubNam	--	GO:00095	--	--	--
rna-Poh01	-2.8203	5.09E-150	1.13E-147	down	Poh01179	Poh01179	hypothetical protein PHYPA_008408 [Physcomitrella patens]	--	A9SGT8_PHYPA SubNam	--	GO:00160	--	--	--
rna-Poh03	-3.11496	1.50E-149	3.33E-147	down	Poh03575	Poh03575	hypothetical protein PHYPA_011843 [Physcomitrella patens]	--	A9SPM0_PHYPA SubNan	--	--	--	--	--
rna-Poh03	-2.83895	3.67E-149	8.09E-147	down	Poh03047	Poh03047	hypothetical protein PHYPA_008405 [Physcomitrella patens]	MANA2_ARATH RecName: Full=Probable alpha-mannosidase At5g13980 {ECO:0000305}; EC=3.2.1.24 {ECO:0	A9SGT5_PHYPA RecNam	Glycosyl hy	GO:00480	--	--	--
rna-Poh00	3.578539	5.88E-149	1.29E-146	up	Poh00006	Poh00006	predicted protein [Physcomitrella patens]	MD15A_ARATH RecName: Full=Mediator of RNA polymerase II transcription subunit 15a;	A9SHI4_PHYPA SubName	--	GO:00165	--	--	--
rna-Poh01	-6.77754	6.59E-149	1.44E-146	down	Poh01317	Poh01317	predicted protein [Physcomitrella patens]	EXPA1_ARATH RecName: Full=Expansin-A1; Short=AtEXPA1; AltName: Full=Alpha-expansin-1; Short=At-EXP1	Q7XJA4_PHYPA SubNam	--	GO:00056	--	--	--
rna-Poh02	5.488533	2.54E-148	5.51E-146	up	Poh02161	Poh02161	hypothetical protein PHYPA_016924 [Physcomitrella patens]	BAG6_ARATH RecName: Full=BAG family molecular chaperone regulator 6 {ECO:0000303|Ref.3}; AltName: F	A9SR80_PHYPA SubNam	--	GO:00095	--	--	--
rna-Poh01	-4.08482	4.66E-148	1.01E-145	down	Poh01996	Poh01996	hypothetical protein PHYPA_022436 [Physcomitrella patens]	EMS1_ARATH RecName: Full=Leucine-rich repeat receptor protein kinase EMS1 {ECO:0000305}; EC=2.7.11.1	A9SDH6_PHYPA SubNam	--	GO:00160	--	--	--
rna-Poh00	-4.40315	1.56E-147	3.34E-145	down	Poh00180	Poh00180	predicted protein [Physcomitrella patens]	--	A9SSK9_PHYPA SubName	--	GO:00160	--	--	--
rna-Poh02	-3.73127	2.61E-147	5.56E-145	down	Poh02758	Poh02758	predicted protein [Physcomitrella patens]	DR100_ARATH RecName: Full=DNA damage-repair/toleration protein DRT100; Flags: Precursor;	A9T3S2_PHYPA SubName	FOG: Leuci	GO:00095	--	--	--
rna-Poh01	-4.6549	3.00E-147	6.34E-145	down	Poh01996	Poh01996	hypothetical protein PHYPA_001563 [Physcomitrella patens]	GDL28_ARATH RecName: Full=GDSL esterase/lipase At1g71250; EC=3.1.1.-; AltName: Full=Extracellular lipas	A9RU59_PHYPA SubNam	--	GO:00055	--	--	--
rna-Poh02	5.047224	3.74E-147	7.89E-145	up	Poh02523	Poh02523	--	--	--	--	--	--	--	--
rna-Poh02	-4.60922	1.75E-146	3.66E-144	down	Poh02000	Poh02000	hypothetical protein PHYPA_018546 [Physcomitrella patens]	UFOG5_MANES RecName: Full=Anthocyanidin 3-O-glucosyltransferase 5; EC=2.4.1.115; AltName: Full=Flavor	A9TDK9_PHYPA SubNam	UDP-glucu	GO:00472	--	--	--
rna-Poh01	5.394761	1.90E-146	3.96E-144	up	Poh01506	Poh01506	hypothetical protein CFP56_69038 [Quercus suber]	--	--	--	--	--	--	--
rna-Poh00	6.220553	2.64E-146	5.49E-144	up	Poh00113	Poh00113	hypothetical protein PHYPA_008459 [Physcomitrella patens]	HSP16_SOYBN RecName: Full=18.5 kDa class I heat shock protein; AltName: Full=HSP 18.5;	A9RN10_PHYPA SubNam	Molecular	GO:00057	--	--	--
rna-Poh01	3.933576	4.28E-146	8.86E-144	up	Poh01092	Poh01092	predicted protein [Physcomitrella patens]	C7101_ARATH RecName: Full=Cytochrome P450 710A1 {ECO:0000303|PubMed:16531502}; EC=1.14.19.41 {	A9RQL1_PHYPA SubNam	Cytochrom	GO:00160	--	--	--
rna-Poh03	-4.18746	5.79E-146	1.19E-143	down	Poh03358	Poh03358	leucine-rich repeat receptor-like protein kinase [Pohlia nutans]	BAME1_ARATH RecName: Full=Leucine-rich repeat receptor-like serine/threonine-protein kinase BAM1; EC=	A0A1P8DYU1_9BRYO Sub	--	GO:00160	--	--	--
rna-Poh03	7.067811	1.07E-145	2.19E-143	up	Poh03642	Poh03642	predicted protein [Physcomitrella patens]	AKR2_ORYSJ RecName: Full=Probable aldo-keto reductase 2; EC=1.1.1.-;	A9TGX3_PHYPA SubNam	Voltage-ga	GO:00164	--	--	--
rna-Poh02	4.460364	1.60E-145	3.26E-143	up	Poh02038	Poh02038	predicted protein [Physcomitrella patens]	U72B1_ARATH RecName: Full=UDP-glycosyltransferase 72B1; EC=2.4.1.-; AltName: Full=Arbutin synthase; Al	A9S3P2_PHYPA RecName	UDP-glucu	GO:00432	--	--	--
rna-Poh03	-3.95717	4.72E-145	9.58E-143	down	Poh03170	Poh03170	leucine-rich repeat receptor-like protein kinase [Pohlia nutans]	Y1154_ARATH RecName: Full=Probable serine/threonine-protein kinase At1g01540; EC=2.7.11.1;	A0A1P8DYZ9_9BRYO Sub	--	GO:00160	--	--	--
rna-Poh03	3.4923	5.06E-145	1.02E-142	up	Poh03322	Poh03322	hypothetical protein PHYPA_026005 [Physcomitrella patens]	RH26_ORYSJ RecName: Full=DEAD-box ATP-dependent RNA helicase 26; EC=3.6.4.13;	A9TI18_PHYPA SubName	ATP-depen	GO:00057	--	--	--
rna-Poh00	-3.98192	1.05E-144	2.10E-142	down	Poh00522	Poh00522	leucine-rich repeat receptor-like protein kinase [Pohlia nutans]	SERK2_ORYSJ RecName: Full=LRR receptor kinase SERK2 {ECO:0000305}; EC=2.7.11.1 {ECO:0000305}; AltNan	A0A1P8DYX8_9BRYO Sub	--	GO:00160	--	--	--

Table 7. DEGs related to flavonoids and plant-pathogen interaction under UV-B radiation of Pohlia nutans.

ID	log2FoldCha	pvalue	padj	regulated	NR	Swissprot
**Flavonoid synthesis pathway**						
rna-Poh0123	2.48716447	0.00035561	0.0006826	up	hypothetical protein PHYPA_029802 [Physcon	TCMO_POPTM RecName: Full=Trans-cinnamate 4-monooxygenase; EC=1.14.13.11; AltName: Full=Ci
rna-Poh0347	-2.1172826	7.8199E-21	5.3707E-20	down	hypothetical protein PHYPA_006574 [Physcon	TCMO_POPKI RecName: Full=Trans-cinnamate 4-monooxygenase; EC=1.14.13.11; AltName: Full=Cin
Novel_00214	1.9579021	2.9335E-13	1.2783E-12	up	hypothetical protein PHYPA_006574 [Physcon	TCMO_POPKI RecName: Full=Trans-cinnamate 4-monooxygenase; EC=1.14.13.11; AltName: Full=Cin
rna-Poh0229	3.18850396	5.0326E-38	7.0791E-37	up	hypothetical protein PHYPA_029802 [Physcon	TCMO_POPKI RecName: Full=Trans-cinnamate 4-monooxygenase; EC=1.14.13.11; AltName: Full=Cin
rna-Poh0325	1.1292693	8.2715E-16	4.2783E-15	up	predicted protein [Physcomitrella patens]	TCMO_POPKI RecName: Full=Trans-cinnamate 4-monooxygenase; EC=1.14.13.11; AltName: Full=Cin
rna-Poh0326	1.76010323	6.1574E-18	3.6244E-17	up	hypothetical protein PHYPA_006574 [Physcon	TCMO_POPKI RecName: Full=Trans-cinnamate 4-monooxygenase; EC=1.14.13.11; AltName: Full=Cin
Novel_00422	1.17291791	3.0366E-07	8.1695E-07	up	predicted protein [Physcomitrella patens]	TCMO_POPKI RecName: Full=Trans-cinnamate 4-monooxygenase; EC=1.14.13.11; AltName: Full=Cin
rna-Poh0117	2.31042892	1.63E-54	3.8649E-53	up	hypothetical protein PHYPA_029802 [Physcon	TCMO_POPKI RecName: Full=Trans-cinnamate 4-monooxygenase; EC=1.14.13.11; AltName: Full=Cin
rna-Poh0117	2.23056019	1.2322E-30	1.3147E-29	up	hypothetical protein PHYPA_008384 [Physcon	TCMO_POPKI RecName: Full=Trans-cinnamate 4-monooxygenase; EC=1.14.13.11; AltName: Full=Cin
rna-Poh0015	3.28688591	4.4782E-64	1.4218E-62	up	hypothetical protein PHYPA_029802 [Physcon	TCMO_POPKI RecName: Full=Trans-cinnamate 4-monooxygenase; EC=1.14.13.11; AltName: Full=Cin
Novel_00898	-1.6055219	5.9083E-06	1.3954E-05	down	cinnamate 4-hydroxylase [Physcomitrella paten	TCMO_POPKI RecName: Full=Trans-cinnamate 4-monooxygenase; EC=1.14.13.11; AltName: Full=Cin
rna-Poh0170	-2.4593451	2.9292E-63	9.1178E-62	down	cinnamate 4-hydroxylase [Physcomitrella paten	TCMO_POPKI RecName: Full=Trans-cinnamate 4-monooxygenase; EC=1.14.13.11; AltName: Full=Cin
Novel_00012	2.35223975	2.163E-07	5.9088E-07	up	predicted protein [Physcomitrella patens]	CHS6_SOYBN RecName: Full=Chalcone synthase 6; EC=2.3.1.74; AltName: Full=Naringenin-chalcone
Novel_00012	1.62604211	6.0701E-06	1.4316E-05	up	predicted protein [Physcomitrella patens]	CHS6_SOYBN RecName: Full=Chalcone synthase 6; EC=2.3.1.74; AltName: Full=Naringenin-chalcone
Novel_00014	2.93975079	2.047E-13	9.0214E-13	up	predicted protein [Physcomitrella patens]	CHS6_SOYBN RecName: Full=Chalcone synthase 6; EC=2.3.1.74; AltName: Full=Naringenin-chalcone
Novel_00062	2.10521405	3.5162E-08	1.0336E-07	up	predicted protein [Physcomitrella patens]	CHS6_SOYBN RecName: Full=Chalcone synthase 6; EC=2.3.1.74; AltName: Full=Naringenin-chalcone
rna-Poh0271	1.94103857	4.1044E-13	1.7668E-12	up	predicted protein [Physcomitrella patens]	CHS6_SOYBN RecName: Full=Chalcone synthase 6; EC=2.3.1.74; AltName: Full=Naringenin-chalcone
rna-Poh0271	2.08906097	1.0152E-17	5.8988E-17	up	predicted protein [Physcomitrella patens]	CHS6_SOYBN RecName: Full=Chalcone synthase 6; EC=2.3.1.74; AltName: Full=Naringenin-chalcone
rna-Poh0289	3.22579928	1.1836E-35	1.5289E-34	up	predicted protein [Physcomitrella patens]	CHS6_SOYBN RecName: Full=Chalcone synthase 6; EC=2.3.1.74; AltName: Full=Naringenin-chalcone
rna-Poh0289	2.78331295	2.2236E-33	2.642E-32	up	predicted protein [Physcomitrella patens]	CHS6_SOYBN RecName: Full=Chalcone synthase 6; EC=2.3.1.74; AltName: Full=Naringenin-chalcone
rna-Poh0222	4.2381559	1.2253E-11	4.7529E-11	up	hypothetical protein PHYPA_029225 [Physcon	CHSY_HYPAN RecName: Full=Chalcone synthase; EC=2.3.1.74; AltName: Full=Naringenin-chalcone s
Novel_00580	1.78654213	1.3703E-13	6.1147E-13	up	predicted protein [Physcomitrella patens]	CHS6_SOYBN RecName: Full=Chalcone synthase 6; EC=2.3.1.74; AltName: Full=Naringenin-chalcone
Novel_00580	1.36760493	7.217E-14	3.2871E-13	up	predicted protein [Physcomitrella patens]	CHS6_SOYBN RecName: Full=Chalcone synthase 6; EC=2.3.1.74; AltName: Full=Naringenin-chalcone
Novel_00583	3.95601581	1.1145E-33	1.3432E-32	up	predicted protein [Physcomitrella patens]	CHS6_SOYBN RecName: Full=Chalcone synthase 6; EC=2.3.1.74; AltName: Full=Naringenin-chalcone
Novel_00645	-3.8158597	7.2605E-14	3.3066E-13	down	predicted protein [Physcomitrella patens]	CHS6_SOYBN RecName: Full=Chalcone synthase 6; EC=2.3.1.74; AltName: Full=Naringenin-chalcone
rna-Poh0094	3.38271154	1.4005E-45	2.5435E-44	up	predicted protein [Physcomitrella patens]	CHS6_SOYBN RecName: Full=Chalcone synthase 6; EC=2.3.1.74; AltName: Full=Naringenin-chalcone
rna-Poh0094	3.35247482	2.6657E-39	3.911E-38	up	predicted protein [Physcomitrella patens]	CHS6_SOYBN RecName: Full=Chalcone synthase 6; EC=2.3.1.74; AltName: Full=Naringenin-chalcone
rna-Poh0096	1.17184425	5.8447E-14	2.6782E-13	up	predicted protein [Physcomitrella patens]	CHS6_SOYBN RecName: Full=Chalcone synthase 6; EC=2.3.1.74; AltName: Full=Naringenin-chalcone
Novel_00739	2.46770773	5.4327E-12	2.1588E-11	up	hypothetical protein PHYPA_029225 [Physcon	CHSY_PINST RecName: Full=Chalcone synthase; EC=2.3.1.74; AltName: Full=Naringenin-chalcone syı
rna-Poh0019	3.26170233	2.1752E-59	6.0326E-58	up	hypothetical protein PHYPA_029225 [Physcon	CHSY_PINST RecName: Full=Chalcone synthase; EC=2.3.1.74; AltName: Full=Naringenin-chalcone syı
Novel_00822	2.10968717	1.0584E-08	3.2601E-08	up	predicted protein [Physcomitrella patens]	CHSY_HYPAN RecName: Full=Chalcone synthase; EC=2.3.1.74; AltName: Full=Naringenin-chalcone s
Novel_00863	2.47505511	5.9689E-06	1.4089E-05	up	predicted protein [Physcomitrella patens]	CHSY_HYPAN RecName: Full=Chalcone synthase; EC=2.3.1.74; AltName: Full=Naringenin-chalcone s
rna-Poh0001	2.24941568	9.4436E-18	5.4955E-17	up	predicted protein [Physcomitrella patens]	CHSY_HYPAN RecName: Full=Chalcone synthase; EC=2.3.1.74; AltName: Full=Naringenin-chalcone s
rna-Poh0001	2.70188414	1.2879E-15	6.5863E-15	up	hypothetical protein PHYPA_029225 [Physcon	CHSY_HYPAN RecName: Full=Chalcone synthase; EC=2.3.1.74; AltName: Full=Naringenin-chalcone s
rna-Poh0001	3.49044959	2.5193E-58	6.7225E-57	up	hypothetical protein PHYPA_029225 [Physcon	CHSY_HYPAN RecName: Full=Chalcone synthase; EC=2.3.1.74; AltName: Full=Naringenin-chalcone s
Novel_00924	2.65008956	4.7991E-11	1.7825E-10	up	hypothetical protein PHYPA_014791 [Physcon	CHSY_EQUAR RecName: Full=Chalcone synthase; EC=2.3.1.74; AltName: Full=Naringenin-chalcone s
Novel_00925	11.1135436	3.1487E-11	1.186E-10	up	hypothetical protein PHYPA_014791 [Physcon	CHSY_EQUAR RecName: Full=Chalcone synthase; EC=2.3.1.74; AltName: Full=Naringenin-chalcone s
rna-Poh0270	3.40758598	1.1239E-81	5.7448E-80	up	hypothetical protein PHYPA_014791 [Physcon	CHSY_EQUAR RecName: Full=Chalcone synthase; EC=2.3.1.74; AltName: Full=Naringenin-chalcone s
Novel_01012	1.42578352	3.3474E-05	7.2637E-05	up	hypothetical protein PHYPA_014791 [Physcon	CHSY_EQUAR RecName: Full=Chalcone synthase; EC=2.3.1.74; AltName: Full=Naringenin-chalcone s
Novel_01012	2.42211279	0.00198192	0.00344647	up	hypothetical protein PHYPA_014791 [Physcon	CHSY_EQUAR RecName: Full=Chalcone synthase; EC=2.3.1.74; AltName: Full=Naringenin-chalcone s
rna-Poh0246	1.29171793	1.9102E-14	9.0614E-14	up	hypothetical protein PHYPA_014791 [Physcon	CHSY_EQUAR RecName: Full=Chalcone synthase; EC=2.3.1.74; AltName: Full=Naringenin-chalcone s
Novel_01062	2.83273745	4.1769E-46	7.7061E-45	up	chalcone synthase [Physcomitrella patens]	CHSY_ARAAL RecName: Full=Chalcone synthase; EC=2.3.1.74; AltName: Full=Naringenin-chalcone s
Novel_01093	5.84615916	7.4627E-18	4.3753E-17	up	hypothetical protein PHYPA_029225 [Physcon	CHSY_PINST RecName: Full=Chalcone synthase; EC=2.3.1.74; AltName: Full=Naringenin-chalcone syı
Novel_01093	3.93050461	0.00360355	0.00603146	up	hypothetical protein PHYPA_029225 [Physcon	CHSY_PINSY RecName: Full=Chalcone synthase; EC=2.3.1.74; AltName: Full=Naringenin-chalcone syı
rna-Poh0239	2.7279125	1.2702E-40	1.941E-39	up	chalcone synthase [Physcomitrella patens]	CHSY_ARAAL RecName: Full=Chalcone synthase; EC=2.3.1.74; AltName: Full=Naringenin-chalcone s
rna-Poh0244	5.90956657	6.7071E-81	3.3448E-79	up	hypothetical protein PHYPA_029225 [Physcon	CHSY_PINST RecName: Full=Chalcone synthase; EC=2.3.1.74; AltName: Full=Naringenin-chalcone syı
rna-Poh0244	8.19774703	0.00046865	0.00088576	up	hypothetical protein PHYPA_029225 [Physcon	CHSY_PINST RecName: Full=Chalcone synthase; EC=2.3.1.74; AltName: Full=Naringenin-chalcone syı
Novel_01113	3.73406605	5.8084E-12	2.3032E-11	up	hypothetical protein PHYPA_029225 [Physcon	CHSY_PINST RecName: Full=Chalcone synthase; EC=2.3.1.74; AltName: Full=Naringenin-chalcone syı
Novel_01113	4.40068701	4.4044E-13	1.892E-12	up	hypothetical protein PHYPA_029225 [Physcon	CHSY_PINST RecName: Full=Chalcone synthase; EC=2.3.1.74; AltName: Full=Naringenin-chalcone syı
Novel_01133	3.77991681	7.9801E-17	4.398E-16	up	hypothetical protein PHYPA_029225 [Physcon	CHSY_PINST RecName: Full=Chalcone synthase; EC=2.3.1.74; AltName: Full=Naringenin-chalcone syı
Novel_01133	3.86526836	6.2229E-17	3.4557E-16	up	hypothetical protein PHYPA_029225 [Physcon	CHSY_PINST RecName: Full=Chalcone synthase; EC=2.3.1.74; AltName: Full=Naringenin-chalcone syı
rna-Poh0291	3.5952411	1.6878E-41	2.648E-40	up	hypothetical protein PHYPA_029225 [Physcon	CHSY_PINST RecName: Full=Chalcone synthase; EC=2.3.1.74; AltName: Full=Naringenin-chalcone syı
rna-Poh0291	3.66726863	1.0888E-93	7.3131E-92	up	hypothetical protein PHYPA_029225 [Physcon	CHSY_PINST RecName: Full=Chalcone synthase; EC=2.3.1.74; AltName: Full=Naringenin-chalcone syı
Novel_00086	1.2847099	6.1946E-07	1.6174E-06	up	predicted protein [Physcomitrella patens]	CFI3_ARATH RecName: Full=Probable chalcone--flavonone isomerase 3; Short=Chalcone isomerase 3;
rna-Poh0050	1.07399963	8.5669E-15	4.1598E-14	up	predicted protein [Physcomitrella patens]	CFI3_ARATH RecName: Full=Probable chalcone--flavonone isomerase 3; Short=Chalcone isomerase 3;
Novel_00662	1.46421501	9.8435E-09	3.0394E-08	up	predicted protein [Physcomitrella patens]	CFI3_ARATH RecName: Full=Probable chalcone--flavonone isomerase 3; Short=Chalcone isomerase 3;
rna-Poh0218	1.48346455	1.3539E-14	6.4827E-14	up	predicted protein [Physcomitrella patens]	CFI3_ARATH RecName: Full=Probable chalcone--flavonone isomerase 3; Short=Chalcone isomerase 3;
Novel_00782	-1.2945894	3.1466E-06	7.6554E-06	down	hypothetical protein PHYPA_003648 [Physcon	VSR1_PEA RecName: Full=Vacuolar-sorting receptor 1; AltName: Full=80 kDa proaleurein-binding prot
rna-Poh0018	-1.4016617	2.2547E-24	1.8392E-23	down	hypothetical protein PHYPA_003648 [Physcon	VSR1_PEA RecName: Full=Vacuolar-sorting receptor 1; AltName: Full=80 kDa proaleurein-binding prot
rna-Poh0168	-1.5581984	1.7752E-22	1.3283E-21	down	hypothetical protein PHYPA_014139 [Physcon	FAP3_ARATH RecName: Full=Fatty-acid-binding protein 3, chloroplastic; Short=AtFAP3; AltName: Fu
rna-Poh0136	-1.5044328	9.2056E-19	5.6832E-18	down	hypothetical protein PHYPA_014139 [Physcon	FAP3_ARATH RecName: Full=Fatty-acid-binding protein 3, chloroplastic; Short=AtFAP3; AltName: Fu
rna-Poh0353	1.47692455	8.5768E-08	2.4341E-07	up	hypothetical protein PHYPA_014530 [Physcon	VSR1_PEA RecName: Full=Vacuolar-sorting receptor 1; AltName: Full=80 kDa proaleurein-binding prot
Novel_00052	-2.4700788	1.2986E-12	5.3996E-12	down	PREDICTED: probable 2-oxoglutarate/Fe(II)-d	DIOX4_ARATH RecName: Full=Probable 2-oxoglutarate-dependent dioxygenase ANS {ECO:0000305};
rna-Poh0344	-3.3107163	2.0096E-89	1.2323E-87	down	PREDICTED: probable 2-oxoglutarate/Fe(II)-d	DIOX4_ARATH RecName: Full=Probable 2-oxoglutarate-dependent dioxygenase ANS {ECO:0000305};
rna-Poh0344	-1.8643523	1.1169E-38	1.6036E-37	down	PREDICTED: probable 2-oxoglutarate/Fe(II)-d	DIOX4_ARATH RecName: Full=Probable 2-oxoglutarate-dependent dioxygenase ANS {ECO:0000305};
rna-Poh0305	-2.4391568	4.0564E-28	3.8966E-27	down	predicted protein [Physcomitrella patens]	DIOX2_ARATH RecName: Full=Probable 2-oxoglutarate-dependent dioxygenase At3g50210; EC=1.14.1
Novel_00629	-1.0034014	0.00310589	0.00525168	down	PREDICTED: leucoanthocyanidin dioxygenase	DIOX5_ARATH RecName: Full=Probable 2-oxoglutarate-dependent dioxygenase At5g05600 {ECO:000
rna-Poh0153	-1.3936337	5.4545E-16	2.8504E-15	down	PREDICTED: leucoanthocyanidin dioxygenase	DIOX5_ARATH RecName: Full=Probable 2-oxoglutarate-dependent dioxygenase At5g05600 {ECO:000
rna-Poh0135	-1.4385894	0.00529897	0.00864926	down	PREDICTED: probable 2-oxoglutarate-depend	DIOX4_ARATH RecName: Full=Probable 2-oxoglutarate-dependent dioxygenase ANS {ECO:0000305};
Novel_00008	4.95423236	2.03E-77	9.2244E-76	up	flavonoid 3',5'-hydroxylase [Pohlia nutans]	C75A1_PETHY RecName: Full=Flavonoid 3',5'-hydroxylase 1; Short=F3'5'H; EC=1.14.13.88; AltName:
Novel_00056	4.6836004	3.3507E-14	1.5622E-13	up	flavonoid 3'-hydroxylase [Pohlia nutans]	F3PH_ARATH RecName: Full=Flavonoid 3'-monooxygenase; EC=1.14.13.21; AltName: Full=Cytochror
Novel_00058	4.96366352	3.4129E-81	1.7179E-79	up	flavonoid 3',5'-hydroxylase [Pohlia nutans]	C75A1_PETHY RecName: Full=Flavonoid 3',5'-hydroxylase 1; Short=F3'5'H; EC=1.14.13.88; AltName:
rna-Poh0273	4.7279686	1.264E-122	1.667E-120	up	flavonoid 3',5'-hydroxylase [Pohlia nutans]	C75A1_PETHY RecName: Full=Flavonoid 3',5'-hydroxylase 1; Short=F3'5'H; EC=1.14.13.88; AltName:
rna-Poh0273	4.69933437	5.69E-128	8.18E-126	up	flavonoid 3',5'-hydroxylase [Pohlia nutans]	C75A1_PETHY RecName: Full=Flavonoid 3',5'-hydroxylase 1; Short=F3'5'H; EC=1.14.13.88; AltName:
rna-Poh0275	4.29198675	2.0962E-68	7.5155E-67	up	flavonoid 3'-hydroxylase [Pohlia nutans]	F3PH_ARATH RecName: Full=Flavonoid 3'-monooxygenase; EC=1.14.13.21; AltName: Full=Cytochror
rna-Poh0080	-1.7923388	4.6378E-23	3.5538E-22	down	flavonoid 3'-hydroxylase [Pohlia nutans]	C7A12_PANGI RecName: Full=Cytochrome P450 CYP736A12; EC=1.14.-.-;
rna-Poh0225	-3.2713216	1.3336E-19	8.5714E-19	down	hypothetical protein PHYPA_030965 [Physcon	F3PH_PETHY RecName: Full=Flavonoid 3'-monooxygenase; EC=1.14.13.21; AltName: Full=Cytochrom
rna-Poh0056	-1.4630151	0.00012437	0.00025273	down	hypothetical protein PHYPA_030965 [Physcon	F3PH_PETHY RecName: Full=Flavonoid 3'-monooxygenase; EC=1.14.13.21; AltName: Full=Cytochrom
Novel_00441	-2.615496	0.00334641	0.00562978	down	flavonoid 3'-hydroxylase [Pohlia nutans]	CYPH1_SALMI RecName: Full=Ferruginol synthase {ECO:0000303|PubMed:23812755}; EC=1.14.13.1
Novel_00466	-3.4930093	1.4147E-06	3.5669E-06	down	hypothetical protein PHYPA_005260 [Physcon	CYPH1_SALMI RecName: Full=Ferruginol synthase {ECO:0000303|PubMed:23812755}; EC=1.14.13.1
rna-Poh0038	1.28455445	1.039E-05	2.384E-05	up	predicted protein [Physcomitrella patens]	F3PH_ARATH RecName: Full=Flavonoid 3'-monooxygenase; EC=1.14.13.21; AltName: Full=Cytochror
rna-Poh0276	-5.7977789	1.4088E-56	3.5603E-55	down	flavonoid 3'-hydroxylase [Pohlia nutans]	C70A2_ARATH RecName: Full=Cytochrome P450 703A2 {ECO:0000303|PubMed:17496121}; EC=1.1
rna-Poh0319	3.04439606	1.2112E-06	3.0717E-06	up	flavonoid 3'-hydroxylase [Pohlia nutans]	C75B3_ORYSJ RecName: Full=Flavonoid 3'-monooxygenase CYP75B3 {ECO:0000305}; EC=1.14.13.2
Novel_00868	-2.7675872	1.3231E-36	1.7702E-35	down	predicted protein [Physcomitrella patens]	F3PH_PETHY RecName: Full=Flavonoid 3'-monooxygenase; EC=1.14.13.21; AltName: Full=Cytochrom
Novel_00921	1.39528453	4.7752E-07	1.2597E-06	up	flavonoid 3'-hydroxylase [Pohlia nutans]	F3PH_PETHY RecName: Full=Flavonoid 3'-monooxygenase; EC=1.14.13.21; AltName: Full=Cytochrom
rna-Poh0268	1.34570372	6.363E-19	3.9584E-18	up	flavonoid 3'-hydroxylase [Pohlia nutans]	F3PH_PETHY RecName: Full=Flavonoid 3'-monooxygenase; EC=1.14.13.21; AltName: Full=Cytochrom
Novel_01008	3.69752261	4.8273E-48	9.4496E-47	up	flavonoid 3'-hydroxylase [Pohlia nutans]	F3PH_PETHY RecName: Full=Flavonoid 3'-monooxygenase; EC=1.14.13.21; AltName: Full=Cytochrom
rna-Poh0248	3.32988944	2.199E-124	2.982E-122	up	flavonoid 3'-hydroxylase [Pohlia nutans]	F3PH_PETHY RecName: Full=Flavonoid 3'-monooxygenase; EC=1.14.13.21; AltName: Full=Cytochrom
rna-Poh0252	-1.4539427	8.2204E-15	3.996E-14	down	flavonoid 3'-hydroxylase [Pohlia nutans]	F3PH_PETHY RecName: Full=Flavonoid 3'-monooxygenase; EC=1.14.13.21; AltName: Full=Cytochrom
rna-Poh0290	-2.7286072	6.9799E-17	3.8636E-16	down	hypothetical protein PHYPA_027326 [Physcon	C98A2_SOYBN RecName: Full=Cytochrome P450 98A2; EC=1.14.-.-;
rna-Poh0094	-2.9616336	5.1108E-18	3.0215E-17	down	hypothetical protein PHYPA_027326 [Physcon	C98A2_SOYBN RecName: Full=Cytochrome P450 98A2; EC=1.14.-.-;
rna-Poh0016	2.25024841	1.7206E-36	2.2955E-35	up	hypothetical protein PHYPA_003131 [Physcon	C98A2_SOYBN RecName: Full=Cytochrome P450 98A2; EC=1.14.-.-;
rna-Poh0223	-2.4132509	7.9641E-07	2.0574E-06	down	hypothetical protein PHYPA_030851 [Physcon	HCT1_ORYSJ RecName: Full=Hydroxycinnamoyltransferase 1 {ECO:0000305}; Short=OsHCT1 {ECO:
Novel_00314	1.60142105	3.2266E-11	1.2135E-10	up	hypothetical protein PHYPA_030849 [Physcon	HCT2_ORYSJ RecName: Full=Hydroxycinnamoyltransferase 2 {ECO:0000305}; Short=OsHCT2 {ECO:
rna-Poh0059	1.56483992	1.7913E-26	1.6046E-25	up	hypothetical protein PHYPA_030849 [Physcon	HCT2_ORYSJ RecName: Full=Hydroxycinnamoyltransferase 2 {ECO:0000305}; Short=OsHCT2 {ECO:
rna-Poh0059	-6.9079047	9.8225E-10	3.2944E-09	down	hypothetical protein PHYPA_030851 [Physcon	HCT1_ORYSJ RecName: Full=Hydroxycinnamoyltransferase 1 {ECO:0000305}; Short=OsHCT1 {ECO:
rna-Poh0199	-1.4934991	4.555E-10	1.5668E-09	down	hypothetical protein PHYPA_003798 [Physcon	HST_ARATH RecName: Full=Shikimate O-hydroxycinnamoyltransferase; EC=2.3.1.133; AltName: Full=
Novel_00757	2.99705983	2.9094E-07	7.8445E-07	up	hypothetical protein PHYPA_010928 [Physcon	HCT2_ORYSJ RecName: Full=Hydroxycinnamoyltransferase 2 {ECO:0000305}; Short=OsHCT2 {ECO:
rna-Poh0017	-2.2033418	2.5824E-13	1.1293E-12	down	PpHCT1 [Physcomitrella patens]	HST_TOBAC RecName: Full=Shikimate O-hydroxycinnamoyltransferase; EC=2.3.1.133; AltName: Full=
rna-Poh0027	-2.4780422	1.078E-12	4.5063E-12	down	hypothetical protein PHYPA_007405 [Physcon	AHT1_ORYSJ RecName: Full=Agmatine hydroxycinnamoyltransferase 1 {ECO:0000303|PubMed:27354
rna-Poh0029	2.19194761	2.5755E-28	2.4975E-27	up	hypothetical protein PHYPA_010928 [Physcon	HST_ARATH RecName: Full=Shikimate O-hydroxycinnamoyltransferase; EC=2.3.1.133; AltName: Full=
Novel_00830	1.71234144	2.035E-06	5.0456E-06	up	hypothetical protein PHYPA_030849 [Physcon	HST_TOBAC RecName: Full=Shikimate O-hydroxycinnamoyltransferase; EC=2.3.1.133; AltName: Full=
rna-Poh0003	-4.1535014	8.2544E-60	2.3252E-58	down	PpHCT1 [Physcomitrella patens]	HST_TOBAC RecName: Full=Shikimate O-hydroxycinnamoyltransferase; EC=2.3.1.133; AltName: Full=
rna-Poh0281	1.21236388	1.484E-09	4.9094E-09	up	hypothetical protein PHYPA_030849 [Physcon	HST_TOBAC RecName: Full=Shikimate O-hydroxycinnamoyltransferase; EC=2.3.1.133; AltName: Full=
rna-Poh0365	2.02913931	4.1748E-26	3.6685E-25	up	hypothetical protein PHYPA_010928 [Physcon	HST_TOBAC RecName: Full=Shikimate O-hydroxycinnamoyltransferase; EC=2.3.1.133; AltName: Full=
Novel_00909	1.0792704	0.00016359	0.00032757	up	predicted protein [Physcomitrella patens]	--
Novel_00993	1.64884798	3.7669E-11	1.4092E-10	up	predicted protein [Physcomitrella patens]	--
rna-Poh0207	1.35003699	7.6606E-31	8.2512E-30	up	predicted protein [Physcomitrella patens]	--
Novel_01110	2.71925359	2.6203E-06	6.4235E-06	up	hypothetical protein PHYPA_003798 [Physcon	HST_ARATH RecName: Full=Shikimate O-hydroxycinnamoyltransferase; EC=2.3.1.133; AltName: Full=
Novel_01110	8.40068677	7.4167E-11	2.718E-10	up	hypothetical protein PHYPA_003798 [Physcon	HST_TOBAC RecName: Full=Shikimate O-hydroxycinnamoyltransferase; EC=2.3.1.133; AltName: Full=
rna-Poh0292	3.17002784	7.2169E-43	1.1905E-41	up	hypothetical protein PHYPA_003798 [Physcon	HST_ARATH RecName: Full=Shikimate O-hydroxycinnamoyltransferase; EC=2.3.1.133; AltName: Full=
Novel_00585	7.36540597	1.3746E-08	4.1891E-08	up	predicted protein [Physcomitrella patens]	C75A6_CAMME RecName: Full=Flavonoid 3',5'-hydroxylase; Short=F3'5'H; EC=1.14.13.88; AltName:
Novel_00643	6.48442457	1.354E-07	3.7667E-07	up	predicted protein [Physcomitrella patens]	C75A6_CAMME RecName: Full=Flavonoid 3',5'-hydroxylase; Short=F3'5'H; EC=1.14.13.88; AltName:
rna-Poh0182	-3.9727699	3.4411E-26	3.0349E-25	down	flavonoid 3',5'-hydroxylase [Pohlia nutans]	C71E1_SORBI RecName: Full=4-hydroxyphenylacetaldehyde oxime monooxygenase; EC=1.14.14.37 {E
Novel_00993	-1.0058316	0.00125434	0.00223699	down	flavonoid 3'-hydroxylase [Pohlia nutans]	F3PH_PETHY RecName: Full=Flavonoid 3'-monooxygenase; EC=1.14.13.21; AltName: Full=Cytochrom
rna-Poh0207	-1.3313336	6.2195E-16	3.2427E-15	down	flavonoid 3'-hydroxylase [Pohlia nutans]	F3PH_PETHY RecName: Full=Flavonoid 3'-monooxygenase; EC=1.14.13.21; AltName: Full=Cytochrom
rna-Poh0226	4.05152768	4.0302E-30	4.2121E-29	up	predicted protein [Physcomitrella patens]	88F1G_MALDO RecName: Full=Phloretin 2'-O-glucosyltransferase {ECO:0000305}; EC=2.4.1.- {ECO:
**Plant-pathogen interaction**						
rna-Poh0026	-1.2416304	9.0816E-13	3.812E-12	down	predicted protein [Physcomitrella patens]	GLPK_ARATH RecName: Full=Glycerol kinase; Short=Glycerokinase; EC=2.7.1.30; AltName: Full=AT
rna-Poh0284	-2.1067475	8.1348E-43	1.3398E-41	down	predicted protein [Physcomitrella patens]	GLPK_ARATH RecName: Full=Glycerol kinase; Short=Glycerokinase; EC=2.7.1.30; AltName: Full=AT
Novel_00247	4.55112834	3.639E-36	4.8013E-35	up	predicted protein [Physcomitrella patens]	CML2_ORYSJ RecName: Full=Putative calmodulin-like protein 2; Flags: Precursor;
rna-Poh0226	4.29180025	2.5615E-61	7.5471E-60	up	predicted protein [Physcomitrella patens]	CML2_ORYSJ RecName: Full=Putative calmodulin-like protein 2; Flags: Precursor;
Novel_00308	4.05006348	1.6532E-65	5.4332E-64	up	predicted protein [Physcomitrella patens]	--
rna-Poh0055	3.89605725	7.008E-143	1.351E-140	up	predicted protein [Physcomitrella patens]	--
Novel_00348	-1.1715106	3.1782E-05	6.9138E-05	down	predicted protein [Physcomitrella patens]	CALM_MOUSC RecName: Full=Calmodulin; Short=CaM;
rna-Poh0372	-1.1968967	1.8776E-11	7.1841E-11	down	predicted protein [Physcomitrella patens]	CALM_MOUSC RecName: Full=Calmodulin; Short=CaM;
Novel_00398	-1.2182746	0.00019597	0.00038861	down	predicted protein [Physcomitrella patens]	CALM_MOUSC RecName: Full=Calmodulin; Short=CaM;
rna-Poh0108	-1.2846521	1.5781E-10	5.6377E-10	down	predicted protein [Physcomitrella patens]	CALM_MOUSC RecName: Full=Calmodulin; Short=CaM;
rna-Poh0169	-1.104789	0.00388981	0.00647503	down	hypothetical protein PHYPA_003661 [Physcon	--
rna-Poh0285	1.37212685	1.0916E-19	7.0469E-19	up	hypothetical protein PHYPA_025254 [Physcon	EFTM_ARATH RecName: Full=Elongation factor Tu, mitochondrial; Flags: Precursor;
Novel_00331	2.17901586	2.5291E-35	3.227E-34	up	hypothetical protein PHYPA_001786 [Physcon	EFTUA_NICSY RecName: Full=Elongation factor TuA, chloroplastic; Short=EF-TuA; Flags: Precursor;
rna-Poh0308	1.92321585	2.0344E-44	3.5306E-43	up	hypothetical protein PHYPA_001786 [Physcon	EFTUA_NICSY RecName: Full=Elongation factor TuA, chloroplastic; Short=EF-TuA; Flags: Precursor;
Novel_00382	3.03015841	9.5266E-48	1.8497E-46	up	hypothetical protein PHYPA_001786 [Physcon	EFTUB_NICSY RecName: Full=Elongation factor TuB, chloroplastic; Short=EF-TuB; Flags: Precursor;
rna-Poh0097	2.78778154	2.9064E-71	1.1205E-69	up	hypothetical protein PHYPA_001786 [Physcon	EFTUB_NICSY RecName: Full=Elongation factor TuB, chloroplastic; Short=EF-TuB; Flags: Precursor;
rna-Poh0090	-2.3419972	4.1423E-86	2.3679E-84	down	hypothetical protein PHYPA_025872 [Physcon	--
rna-Poh0090	1.45338211	1.9025E-18	1.1538E-17	up	hypothetical protein PHYPA_025254 [Physcon	EFTM_ARATH RecName: Full=Elongation factor Tu, mitochondrial; Flags: Precursor;
rna-Poh0331	1.70098994	9.021E-24	7.1442E-23	up	hypothetical protein PHYPA_027649 [Physcon	EFTM_ARATH RecName: Full=Elongation factor Tu, mitochondrial; Flags: Precursor;
rna-Poh0131	1.41249888	7.6875E-20	5.0085E-19	up	hypothetical protein PHYPA_027649 [Physcon	EFTM_ARATH RecName: Full=Elongation factor Tu, mitochondrial; Flags: Precursor;
Novel_01087	1.46256881	1.7265E-10	6.1485E-10	up	predicted protein [Physcomitrella patens]	HS903_ARATH RecName: Full=Heat shock protein 90-3 {ECO:0000305}; Short=AtHSP90.3 {ECO:000
rna-Poh0240	1.21390963	7.5322E-31	8.115E-30	up	predicted protein [Physcomitrella patens]	HS903_ARATH RecName: Full=Heat shock protein 90-3 {ECO:0000305}; Short=AtHSP90.3 {ECO:000
Novel_01106	1.73686101	1.7261E-15	8.7478E-15	up	predicted protein [Physcomitrella patens]	HS903_ARATH RecName: Full=Heat shock protein 90-3 {ECO:0000305}; Short=AtHSP90.3 {ECO:000
rna-Poh0235	1.49016134	1.175E-44	2.0591E-43	up	predicted protein [Physcomitrella patens]	HS903_ARATH RecName: Full=Heat shock protein 90-3 {ECO:0000305}; Short=AtHSP90.3 {ECO:000
rna-Poh0312	-1.7020382	6.3819E-08	1.8309E-07	down	hypothetical protein PHYPA_019626 [Physcon	CNGC2_ARATH RecName: Full=Cyclic nucleotide-gated ion channel 2; Short=AtCNGC2; AltName: Fu
rna-Poh0063	-1.3114857	0.01420172	0.02172174	down	hypothetical protein PHYPA_019626 [Physcon	CNGC2_ARATH RecName: Full=Cyclic nucleotide-gated ion channel 2; Short=AtCNGC2; AltName: Fu
rna-Poh0198	-1.3805136	0.00058577	0.00109182	down	hypothetical protein PHYPA_022464 [Physcon	CN15C_MEDTR RecName: Full=Protein CNGC15c {ECO:0000303|PubMed:27230377}; AltName: Full
rna-Poh0295	-1.1188161	0.00561525	0.00913011	down	hypothetical protein PHYPA_022464 [Physcon	CNGC7_ARATH RecName: Full=Putative cyclic nucleotide-gated ion channel 7; AltName: Full=Cyclic r
rna-Poh0300	-1.3363463	7.0393E-13	2.9804E-12	down	hypothetical protein PHYPA_020028 [Physcon	DRM1L_ARATH RecName: Full=DNA (cytosine-5)-methyltransferase DRM1; EC=2.1.1.37; AltName: F
rna-Poh0158	-1.4492685	4.4631E-17	2.504E-16	down	hypothetical protein PHYPA_020028 [Physcon	DRM1L_ARATH RecName: Full=DNA (cytosine-5)-methyltransferase DRM1; EC=2.1.1.37; AltName: F
rna-Poh0129	-1.3353219	3.6039E-28	3.4713E-27	down	hypothetical protein PHYPA_019738 [Physcon	DRM2_ARATH RecName: Full=DNA (cytosine-5)-methyltransferase DRM2; EC=2.1.1.37; AltName: Fu
Novel_01092	1.74647824	1.2593E-14	6.0425E-14	up	predicted protein [Physcomitrella patens]	CNGC5_ARATH RecName: Full=Probable cyclic nucleotide-gated ion channel 5; Short=AtCNGC5; AltN
Novel_01132	1.2798323	1.1621E-10	4.1954E-10	up	predicted protein [Physcomitrella patens]	--
rna-Poh0348	-2.3262595	5.3773E-23	4.1087E-22	down	hypothetical protein PHYPA_017732 [Physcon	CNG15_ARATH RecName: Full=Putative cyclic nucleotide-gated ion channel 15; AltName: Full=Cyclic
Novel_00050	3.41002962	1.1368E-50	2.4074E-49	up	hypothetical protein PHYPA_016601 [Physcon	HS905_ARATH RecName: Full=Heat shock protein 90-5, chloroplastic {ECO:0000305}; Short=AtHSP9
rna-Poh0360	2.93694349	4.595E-133	7.342E-131	up	hypothetical protein PHYPA_016601 [Physcon	HS905_ARATH RecName: Full=Heat shock protein 90-5, chloroplastic {ECO:0000305}; Short=AtHSP9
rna-Poh0081	-4.3598375	1.639E-16	8.8512E-16	down	predicted protein [Physcomitrella patens]	ENPL_CATRO RecName: Full=Endoplasmin homolog; AltName: Full=Glucose-regulated protein 94 hon
Novel_00526	-2.2953665	1.4918E-07	4.1343E-07	down	hypothetical protein AXG93_1217s1120 [Marc	HS906_ARATH RecName: Full=Heat shock protein 90-6, mitochondrial {ECO:0000305}; Short=AtHSP
Novel_00526	-1.310296	1.8861E-12	7.7429E-12	down	hypothetical protein AXG93_1217s1120 [Marc	HS906_ARATH RecName: Full=Heat shock protein 90-6, mitochondrial {ECO:0000305}; Short=AtHSP
rna-Poh0319	-1.5479773	1.4698E-31	1.6326E-30	down	hypothetical protein AXG93_1217s1120 [Marc	HS906_ARATH RecName: Full=Heat shock protein 90-6, mitochondrial {ECO:0000305}; Short=AtHSP
Novel_00627	3.66217901	1.3787E-95	9.861E-94	up	hypothetical protein PHYPA_016601 [Physcon	HS905_ARATH RecName: Full=Heat shock protein 90-5, chloroplastic {ECO:0000305}; Short=AtHSP9
rna-Poh0152	3.58144712	6.672E-195	3.297E-192	up	hypothetical protein PHYPA_016601 [Physcon	HS905_ARATH RecName: Full=Heat shock protein 90-5, chloroplastic {ECO:0000305}; Short=AtHSP9
rna-Poh0182	1.04780211	1.8031E-08	5.44E-08	up	hypothetical protein PHYPA_024148 [Physcon	HS906_ARATH RecName: Full=Heat shock protein 90-6, mitochondrial {ECO:0000305}; Short=AtHSP
rna-Poh0250	1.02030233	2.494E-07	6.7695E-07	up	hypothetical protein PHYPA_024148 [Physcon	HS906_ARATH RecName: Full=Heat shock protein 90-6, mitochondrial {ECO:0000305}; Short=AtHSP
rna-Poh0264	-2.0352457	6.3117E-13	2.6866E-12	down	predicted protein [Physcomitrella patens]	ENPL_CATRO RecName: Full=Endoplasmin homolog; AltName: Full=Glucose-regulated protein 94 hon
rna-Poh0110	-1.0171875	1.1236E-06	2.8599E-06	down	hypothetical protein PHYPA_027059 [Physcon	SGT1_ORYSJ RecName: Full=Protein SGT1 homolog {ECO:0000250|UniProtKB:Q08446}; Short=OsS
rna-Poh0257	-1.355053	6.178E-11	2.2788E-10	down	predicted protein [Physcomitrella patens]	SGT1_ORYSJ RecName: Full=Protein SGT1 homolog {ECO:0000250|UniProtKB:Q08446}; Short=OsS
rna-Poh0334	-1.6301938	1.6636E-12	6.8613E-12	down	predicted protein [Physcomitrella patens]	CCAMK_LOTJA RecName: Full=Calcium and calcium/calmodulin-dependent serine/threonine-protein ki
rna-Poh0335	-2.6403944	2.7545E-15	1.3788E-14	down	hypothetical protein PHYPA_008310 [Physcon	ERG3_ORYSJ RecName: Full=Elicitor-responsive protein 3; AltName: Full=16 kDa phloem protein; Alt

Supplementary Table 1 Primers used in quantitative RT-PCR analysis.

Gene ID	Gene symbol	Primer name	Primer sequence (5’-3’)
LNC_000155	PnCHS-1	PnCHS-1-F	ACGCCTTGGGATTCCGCTCT[illegible]
		PnCHS-1-R	GGGAAGGTGAGGACGTTTA[illegible]
LNC_000244	PnCHS-2	PnCHS-2-F	CGTCGCCTTGGTCTTGCACT[illegible]
		PnCHS-2-R	ACATGAAGGTGGCAGACAG[illegible]
LNC_000398	PnC4H-1	PnC4H-1-F	CAAGAAGCACGCACACGGA[illegible]
		PnC4H-1-R	CCAACATCACCACCGACTG[illegible]
LNC_000418	PnC4H-2	PnC4H-2-F	TGCGATGGCTCTTCTTCACG[illegible]
		PnC4H-2-R	TGGCTATGGACCTGAGTAC[illegible]
LNC_000980	PnC4H-3	PnC4H-3-F	TGAGAGTGCTGGCGGAGAT[illegible]
		PnC4H-3-R	GGCAGAGGCAATAAATCCA[illegible]
LNC_001069	PnF3'5'H-1	PnF3'5'H-1-F	CCAGGATTGCGGTGTTGAA[illegible]
		PnF3'5'H-1-R	CGCTTGCTCACTGGTGTTA[illegible]
LNC_002231	PnFLS-1	PnFLS-1-F	AGTACCTCCGCATACCACCT[illegible]
		PnFLS-1-R	CACACCCACCGACTTACGC[illegible]
LNC_002393	PnCHI-1	PnCHI-1-F	GCTATGGCTGGACAAGGCA[illegible]
		PnCHI-1-R	TGAGTTGGTCGTGGATGGG[illegible]
LNC_002411	PnF3'H-1	PnF3'H-1-F	TCCTAAACGCCGCTGAGAC[illegible]
		PnF3'H-1-R	GCATCACCAAACAAAGCCC[illegible]
LNC_002724	PnCHI-2	PnCHI-2-F	GCAGCGACCACATTACAGC[illegible]
		PnCHI-2-R	ACGCAAAGTGAGGCTCGCA[illegible]
LNC_002773	PnF3'5'H-2	PnF3'5'H-2-F	GCCATCTCCAACGCCTTGTT[illegible]

Supplementary Table 1. Summary of reads mapped to the Pohlia nutans reference genome.

Sample	Total Reads	Reads mapped	Unique mapped	Multi mapped	'+' mapped	'-' mapped
CK_1	79469444	40622188 (51.12%)	35697321 (44.92%)	12239811 (6.20%)	17839738 (22.45%)	17857583 (22.47%)
CK_2	98500292	48726498 (49.47%)	42411324 (43.06%)	16007637 (6.41%)	21193549 (21.52%)	21217775 (21.54%)
CK_3	88589850	46989655 (53.04%)	41394267 (46.73%)	14054358 (6.32%)	20689391 (23.35%)	20704876 (23.37%)
UV-B_1	86334606	52768543 (61.12%)	46515322 (53.88%)	16117273 (7.24%)	23245817 (26.93%)	23269505 (26.95%)
UV-B_2	83986726	49692638 (59.17%)	43189364 (51.42%)	17526197 (7.74%)	21580859 (25.70%)	21608505 (25.73%)
UV-B_3	95201204	60686612 (63.75%)	53654058 (56.36%)	17929434 (7.39%)	26812209 (28.16%)	26841849 (28.19%)
