# Supplementary material for: Genome-Wide Analysis of Long Non-Coding RNAs Related to UV-B Radiation in the Antarctic Moss Pohlia nutans
Source: Int J Mol Sci. 2023 Mar 17;24(6):5757. doi: 10.3390/ijms24065757 (PMC10051584; doi:10.3390/ijms24065757)
Supplement: Supplementary file 1 [file ijms-24-05757-s001.zip › Supplementary Table8.pdf]

Supplementary Table 1 Primers used in quantitative RT-PCR analysis.

Gene ID	Gene symbol	Primer name	Primer sequence (5'-3')	Melting temperature (°C)	Product length (bp)
LNC_000155	PnCHS-1	PnCHS-1-F	ACGCCTTGGGATTCCGCTCTAA	60.1	140
		PnCHS-1-R	GGGAAGGTGAGGACGTTTAGGG	58.4	
LNC_000244	PnCHS-2	PnCHS-2-F	CGTCGCCTTGGTCTTGCACTAC	60.1	150
		PnCHS-2-R	ACATGAAGGTGGCAGACAGAGT	57.6	
LNC_000398	PnC4H-1	PnC4H-1-F	CAAGAAGCACGCACACGGACAC	60.1	150
		PnC4H-1-R	CCAACATCACCACCGACTGCAA	59.7	
LNC_000418	PnC4H-2	PnC4H-2-F	TGCGATGGCTCTTCTTCACGAT	58.8	134
		PnC4H-2-R	TGGCTATGGACCTGAGTACGAT	56.5	
LNC_000980	PnC4H-3	PnC4H-3-F	TGAGAGTGCTGGCGGAGATGAT	59.5	187
		PnC4H-3-R	GGCAGAGGCAATAAATCCACCC	57.7	
LNC_001069	PnF3'5'H-1	PnF3'5'H-1-F	CCAGGATTGCGGTGTTGAAGTA	57	199
		PnF3'5'H-1-R	CGCTTGCTCACTGGTGTTACTT	57.5	
LNC_002231	PnFLS-1	PnFLS-1-F	AGTACCTCCGCATACCACCTCA	58.6	187
		PnFLS-1-R	CACACCCACCGACTTACGCAAA	59.7	
LNC_002393	PnCHI-1	PnCHI-1-F	GCTATGGCTGGACAAGGCAACA	59.5	185
		PnCHI-1-R	TGAGTTGGTCGTGGATGGGATT	57.7	
LNC_002411	PnF3'H-1	PnF3'H-1-F	TCCTAAACGCCGCTGAGACAAA	58.6	121
		PnF3'H-1-R	GCATCACCAAACAAAGCCCTTC	57	
LNC_002724	PnCHI-2	PnCHI-2-F	GCAGCGACCACATTACAGCATC	58.6	154
		PnCHI-2-R	ACGCAAAGTGAGGCTCGCAAAT	60	
LNC_002773	PnF3'5'H-2	PnF3'5'H-2-F	GCCATCTCCAACGCCTTGTTCT	59.5	154

		PnF3'5'H-2-R	CCCGAAACCATGAGTCCGTCAA	58.9	
LNC_002856	PnCHI-3	PnCHI-3-F	CCTCTCGACTCCGTCAGCCATA	59.4	176
		PnCHI-3-R	TGCTGTGCCTGTTGCTCATTCT	59.1	
LNC_002984	PnDRF	PnDRF-F	CCTCGTTCTCACACACGCACTT	59.4	197
		PnDRF-R	TGAGCGTTGTGTCCACCTTTCG	59.7	
LNC_003044	PnF3'H-2	PnF3'H-2-F	GCTGCTTGAGTTCCCTCTCTGA	58.1	134
		PnF3'H-2-R	GCCGCAAGAGTCCGAAGCTATA	58.6	
LNC_003359	PnF3'5'H-3	PnF3'5'H-3-F	CCACGAAGTACAGGACGCACAT	59	120
		PnF3'5'H-3-R	GGATGAACACACGAAACGGGAG	57.9	
LNC_003464	PnF3'5'H-4	PnF3'5'H-4-F	AACGTGCCAAGTCCTCCATTGA	58.5	122
		PnF3'5'H-4-R	CAGGATGACCAGTGCGTTGACA	59.2	
LNC_003677	PnC4H-4	PnC4H-4-F	CGCAGCAATGCCTTGACACCAT	60.6	153
		PnC4H-4-R	GGACTCGGACCTCAGCACTGAT	59.8	
LNC_004098	PnC4H-5	PnC4H-5-F	ACGGATCTACAGGAGCGCCATA	59.1	188
		PnC4H-5-R	AATCGCCATCGCCAGCCACTTA	61	
LNC_004152	PnF3'H-3	PnF3'H-3-F	AATGGCAGCGAAGATCAGGAAG	57.1	143
		PnF3'H-3-R	TGCTCCTCGTGCTTACTGTTGA	58	
LNC_004260	PnFLS-2	PnFLS-2-F	GCACTACCGCCACTACAAGCAA	59.5	170
		PnFLS-2-R	ACGAGCGTTGAACTGCACTTGA	59.4	
LNC_000165	PnWRKY25-1	PnWRKY25-1-F	TGGAACTCGACTGGTGCTTCTT	57.9	147
		PnWRKY25-1-R	CAGCCGTGGAGGTTCAGGTATC	58.8	
LNC_000344	PnRPS2-1	PnRPS2-1-F	TCCTGGTCAGCCTGAAGTCCTA	58.2	195
		PnRPS2-1-R	AAGCATCGCAGTTGTGAGACAG	57.6	
LNC_000458	PnKCS-1	PnKCS-1-F	TCCGCATCTCCTACCTCCTCTT	58.1	198
		PnKCS-1-R	GCGAGAACGAGACAGAAGGACT	58.2	
LNC_000490	PnRPS2-2	PnRPS2-2-F	TCGCCGTTCTCGTTGGTGTAAG	59.3	135

		PnRPS2-2-R	GCAATCTCCGTAACAGCGTTCA	57.7	
LNC_000595	PnPR1-1	PnPR1-1-F	CCACTAACCAGCACTTCCACAA	56.8	132
		PnPR1-1-R	TGCCTCGTAATCCCTGAATCCT	57	
LNC_001246	PnPR1-2	PnPR1-2-F	TGAGCGTCCAGAGTTCCGAGAT	59.2	159
		PnPR1-2-R	CCAGTCGATGGTGCAGGTAGAT	57.9	
LNC_001287	PnRPS2-3	PnRPS2-3-F	AGCGGTTACTGGTCACTCTACA	56.9	187
		PnRPS2-3-R	AACGGGTTCTATTTCCGACTGA	55.5	
LNC_001473	PnKCS-2	PnKCS-2-F	CCTTCTCCCTCCTCCTCCGAAT	58.7	180
		PnKCS-2-R	AGGTGCCGCTTGACGAAGATAC	59.1	
LNC_001572	PnRPM1-1	PnRPM1-1-F	GCGTCCAATCCAGGTGTCCAAA	59.4	168
		PnRPM1-1-R	GCGTCTAGTGCTTCCTGCCAAA	59.5	
LNC_002009	PnRPM1-2	PnRPM1-2-F	TCCGTCCAGCGTAACAGATCCA	59.5	130
		PnRPM1-2-R	GCCATGTCGTGTCGTCTTGTGT	59.8	
LNC_002147	PnRPM1-3	PnRPM1-3-F	GCCATAGCCACCACACCATCCA	60.9	174
		PnRPM1-3-R	ACGGCAGACGGTTCACAAGGAA	60.7	
LNC_002202	PnPR1-3	PnPR1-3-F	CACTCCGAGAACGCAGCCATAG	59.4	155
		PnPR1-3-R	GAGTTGTTGCAGCAGCGATGTC	59.1	
LNC_002323	PnRPS2-4	PnRPS2-4-F	TCGCTCAGTGTCGGCAAGAACT	60.5	120
		PnRPS2-4-R	GCAGGAAGATGGAAGCGGAGAC	59.4	
LNC_002877	PnWRKY25-2	PnWRKY25-2-F	TTCGTCGCAGAGGACAAGTGTA	57.8	193
		PnWRKY25-2-R	GCTTGGACCGTGAATGGATTTG	56.6	
LNC_003241	PnNOS-1	PnNOS-1-F	CCCGCCCTTTCCATCTTCCTCA	60.3	147
		PnNOS-1-R	GCCACGAAAGCAGCACACAAGA	60.5	
LNC_003258	PnPR1-4	PnPR1-4-F	TCCAGTGCGGCATACAGAAGGT	60	178
		PnPR1-4-R	CACATCTGCAAGCTCGCCAAGA	59.8	
LNC_003575	PnNOS-2	PnNOS-2-F	ACTCCTTCACACTTCGGCAGAA	57.9	144

		PnNOS-2-R	ATTACCACACTTCCGCCCGTTT	58.8	
LNC_003707	PnEDS1	PnEDS1-F	TACCGTCACTGTTCGCAATGCT	58.9	168
		PnEDS1-R	AGGGTGTGCCAAACGAGACTTT	58.7	
LNC_003749	PnWRKY1	PnWRKY1-F	GCTGCCAATACAAAGCCATGTG	57.2	194
		PnWRKY1-R	GTTACTGCTTGCCATCTTGACA	55.3	
LNC_004214	PnRPM1-4	PnRPM1-4-F	GCTCGCATCAAGACTGTGAACC	58.3	162
		PnRPM1-4-R	GCTCCAAGTTGAGTCGCCTACA	58.7	
